# Chiral Water-Soluble Molecular Capsules With Amphiphilic Interiors

**DOI:** 10.3389/fchem.2022.883093

**Published:** 2022-04-14

**Authors:** Arkadiusz Marek Sakowicz, Agnieszka Szumna

**Affiliations:** Institute of Organic Chemistry, Polish Academy of Sciences, Warsaw, Poland

**Keywords:** host–guest system, salt bridge, self-assembly, supramolecular chemistry, water chemistry

## Abstract

We present the synthesis of new chiral water-soluble dimeric capsules by the multicomponent Mannich reaction between charged amino acids (glutamic acid or arginine), resorcinarene, and formaldehyde and by subsequent self-assembly. The zwitterionic character of the backbones enables electrostatic interactions between arms and induces self-assembly of dimeric capsules, namely, (L-**ArgR**)_2_ and (L-**GluR**)_2_, in water with a wide range of pH, as demonstrated by NMR, diffusion coefficient measurement, and circular dichroism. The assembly/disassembly processes are fast on the NMR timescale. This mode of dimerization leaves side chains available for additional interactions and creates chiral cavities of mixed hydrophobic/hydrophilic character. According to this characteristic, capsules do not bind fully nonpolar or fully polar guests but effectively encapsulate a variety of chiral molecules with mixed polar/apolar characters (aliphatic and aromatic aldehydes, epoxides, alcohols, carboxylic acids, amines, and amino acids) with moderate strength. We also demonstrate the formation of heterocapsules (**GluR**) (**ArgR**) (homo- and heterochiral) that utilize additional interactions between charged acidic and basic side chains and have better encapsulation properties than those of the homodimers.

## 1 Introduction

Water has been chosen by nature as an environment for all biologically relevant processes including chemical reactions, molecular recognition, and signaling (Sheldon, 2008; Butler and Coyne, 2010; Simon and Li, 2012; Gawande et al., 2013; Lipshutz and Ghorai, 2014; Sheldon, 2016; Sheldon, 2017). However, most of these processes do not occur in the bulk water environment but within isolated nanocompartments that shield interacting partners from competitive external conditions. To mimic this compartmentalization concept, chemists aim at the creation of artificial molecular container molecules (capsules and cages) that operate under various conditions. For obtaining water-soluble and water-stable capsules, numerous covalent and noncovalent strategies have been tested, involving self-assembly by coordination bonds (Yi et al., 2012; Yamashina et al., 2014; Cullen et al., 2015; Taylor et al., 2019; Percástegui et al., 2020), electrostatic interactions (Corbellini et al., 2005; Martin et al., 2011; Zadmard et al., 2013), or hydrophobic clustering (Gibb and Gibb, 2004; Hiraoka et al., 2010; Kondo et al., 2013; Suzuki et al., 2013; Jordan and Gibb, 2015; Suzuki et al., 2016; Kondo et al., 2017; Zhan et al., 2018; Hanafusa et al., 2020; Ashbaugh et al., 2021). Additional stabilization coming from interactions with guests (templation approach) is often beneficial or even crucial for stability (Zhang et al., 2013; Ayhan et al., 2015; Tiefenbacher et al., 2015; Rahman et al., 2020), which, however, blocks the cavity for further applications. Although the self-assembly of container molecules in water is now quite well developed, the rational design of the cavity surface with properly spatially arranged binding sites (Adriaenssens and Ballester, 2013), preferably in a chiral way, remains challenging. A functional chiral cavity is indispensable for the effective separation of enantiomers or catalytic purposes. While a set of chiral capsules that are soluble in organic solvents is quite large (Tokunaga and Rebek, 1998; Rivera et al., 1998; Rivera et al., 2001; Mateos-Timoneda et al., 2004; Sansone et al., 2004; Seeber et al., 2006; Katagiri et al., 2007; Castilla et al., 2014; Brown et al., 2015; Liu et al., 2015; Beaudoin et al., 2016; Chen et al., 2017; Gropp et al., 2018; Kohlhaas et al., 2019; Guo et al., 2020; Nie et al., 2020; Ning et al., 2020), there is only a limited number of examples of capsules that are water-soluble and chiral. The examples include cages based on covalent bonds and coordination bonds (Bolliger et al., 2013; He et al., 2018) or cages obtained by internal functionalization with a chiral appendage (Watfa et al., 2019).

Alongside these important advances, we describe the construction of water-soluble molecular capsules using natural chiral building blocks, amino acids. We demonstrate that electrostatic and hydrophobic interactions are effective in inducing their self-assembly in water. We also demonstrate that such self-assembled capsules have cavities that are capable of encapsulation of chiral molecules with a variety of polar functional groups (epoxides, alcohols, acids, and amines) in a competitive aqueous environment.

## 2 Materials and Methods

### 2.1 Synthesis

#### 2.1.1 Synthesis of (L-GluR)_2_

Resorcin[4]arene **R** (Gibb et al., 1996) (2.16 g, 3 mmol), L-glutamic acid (2.21 g 15 mmol), and formaldehyde (40% aqueous solution, 0.36 ml, 12 mmol) were added to the mixture of DMF and water (1:3, 80 ml). The solution was heated at 60°C and stirred at that temperature for 3 days. After cooling, the reaction was evaporated, and the precipitate was washed with water, acetonitrile, again with water, and dried to get the product in a yield of 50%.

#### 2.1.2 Synthesis of (L-ArgR)_2_

L-Arginine monohydrochloride (0.44 g 2.01 mmol) was dissolved in water (pH 3.5, 5 ml). Further, methanol (5 ml), resorcin [4] arene **R** (0.30 g, 0.41 mmol), and formaldehyde (40% aqueous solution, 0.12 ml, 1.64 mmol) were added. The solution was heated at 60°C and stirred at that temperature for 1 day. After cooling, the reaction was evaporated to dryness, and the product was purified on the Sephadex LH-20 column to get the product in a yield of 47%.

#### 2.1.3 Synthesis of (D-GluR + L-ArgR)

To an aqueous solution of (D-**GluR**)_2_ (5 mg, 2 μmol, in 2 ml of water at pH 5.0), an aqueous solution of (L-**ArgR**)_2_ (5 mg, 1.5 µmol, in 2 ml of water at pH 5.0) was added. The precipitate formed was washed with water (2 × 5 ml) and dried. The product was obtained with a yield of 82%.

#### 2.1.4 Synthesis of (L-GluR + L-ArgR)

To an aqueous solution of (D-**GluR**)_2_ (5 mg, 2 μmol, in 2 ml of water at pH 5.0), an aqueous solution of (L-**ArgR**)_2_ (5 mg, 1.5 µmol, in 2 ml of water at pH 5.0) was added. The precipitate formed was washed with water (2 × 5 ml) and dried. The product was obtained with a yield of 80%.

For experimental details and full characterization see Supplementary Data.

### 2.2 NMR Experiments

^1^H and ^13^C NMR spectra were recorded at 303 K on Bruker 400 MHz and at 298 K on Varian VNMRS 600 MHz instruments with a residual solvent signal as an internal standard. The^13^C NMR data in water were recorded without standards.

All 2D NMR spectra were recorded at 298 K on a Varian 600 MHz instrument. The use of deuterated solvent did not influence the properties of the investigated capsules.

^1^H DOSY experiments were performed on a Varian VNMRS-600 spectrometer at 298 K equipped with a 5-mm PFG AutoXID (^1^H/X = ^15^N-^31^P) probe. DOSY experiments were run with the DPFGDSTE (with convection compensation) pulse sequence for measurements in THF-d8 and benzene-d6 solutions. The gradient strengths were incremented as a square dependence in the range from 6 to 55 G/cm. A total of 16 transients (with an interleave option) were recorded for each increment with 3.2 s acquisition time and 1 s relaxation delay (overall experiment time *ca.* 18–20 min). The duration of magnetic field gradients (δ) was 1.5–2 ms, whereas a diffusion delay (Δ) was chosen as 50–150 ms. Other parameters included the following: a sweep width of 12,000 Hz, and 32 K data points. The data were processed using Varian DOSY software. The hydrodynamic diameters d_H_ (d_H_ = 2r_H_) of the species are calculated using the Einstein–Stokes equation as follows:

$$r_{H}=\frac{k_{b}T}{6\mathrm{\pi \eta D}}$$

Where

$$k_{b}-\mathrm{Boltzmann}\;\mathrm{constant,}$$

T−temperature,

η−viscosity coefficient, and

D−diffusion coeffcient.

The pH of the samples was set by NaOD and DCl solutions and measured using a FiveEasy Plus pH meter FP20. Complexation constants were calculated using HypNMR 2008 (Frassineti et al., 1995; Frassineti et al., 2003).

### 2.3 Density Functional Theory Calculations

All calculations were performed using the density functional theory (DFT) approach using Gaussian 09 program suite (Frisch et al., 2016). Geometry was optimized with the B3LYP functional and by employing the 6-31+G(d) basis set. Solvent effects were considered within the SCRF theory and using the polarized continuum model (PCM) approach to model the interaction with the solvent. Excited electronic states were determined at the wb97xd/6-31+G(d) level by means of the time-dependent DFT (TD DFT) approach (200 excited states in each case). The ECD spectra were simulated by overlapping Gaussian functions for each transition in which the width of the band is 1/e, height is fixed at 0.16 eV, and the resulting intensities of the combined spectra were scaled to the experimental values (using UV–VIS spectra as references). Model compounds, without any additional chromophores derived from amino acids, were used for theoretical calculations. Aliphatic chains at the lower rim were shortened since their lengths have negligible influence on UV–VIS and ECD spectra.

## 3 Original Research

### 3.1 Results and Discussion

#### 3.1.1 Design and Synthesis

**FIGURE 1:**
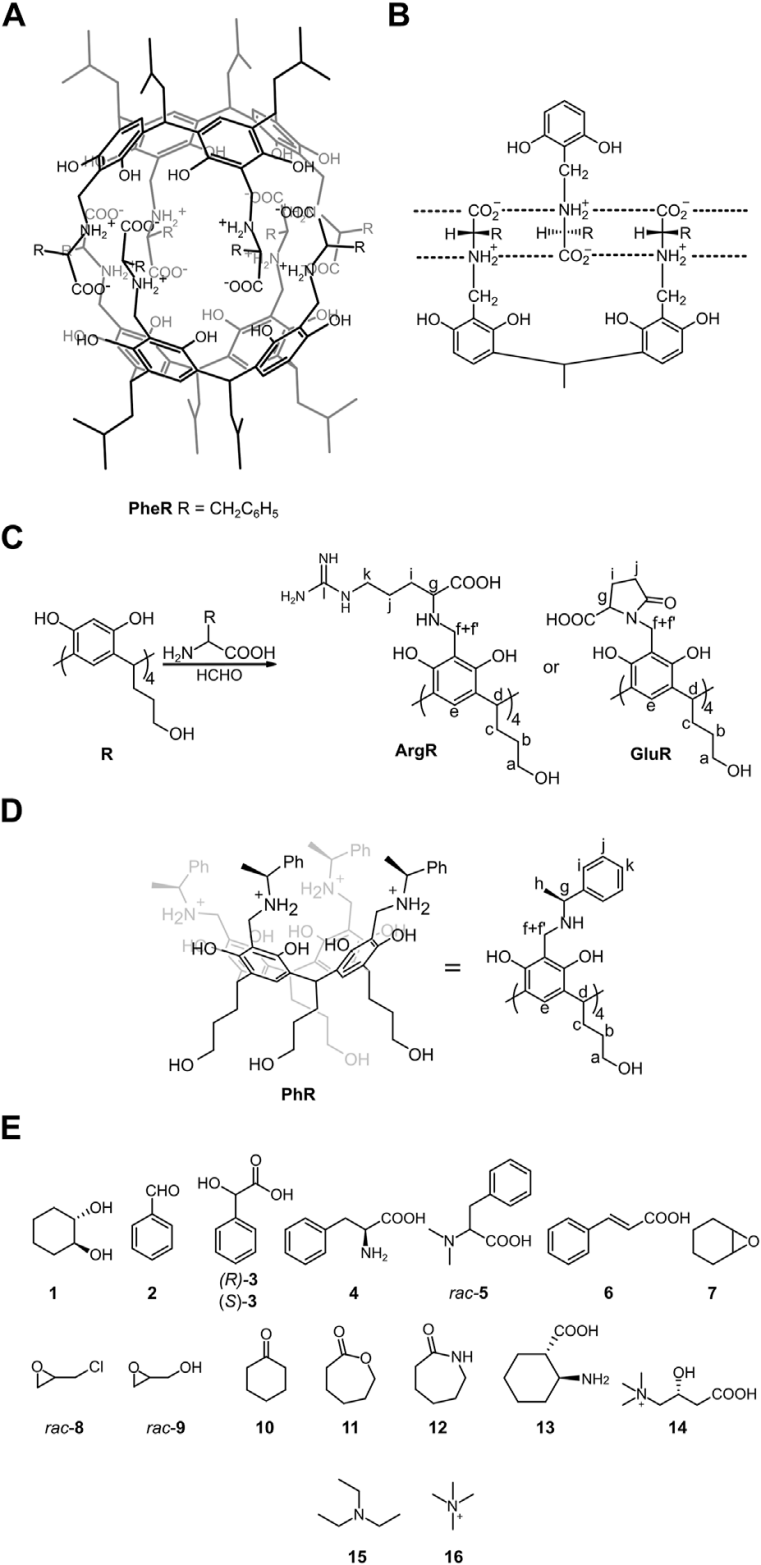
Structures of the compounds used in this study along with notation of the NMR signals: **(A,B)** hydrophobic capsule (L-**PheR**)_2_ and its binding motif (ref. Kuberski and Szumna, 2009); **(C)** synthesis of water-soluble cavitands; **(D)** structure of reference cavitand **PhR** (ref. Setner and Szumna, 2019); and **(E)** guest molecules.

We have previously presented a hydrophobic chiral cavitand made of phenylalanine attached to a resorcin [4] arene skeleton (L-**PheR**, Figure 1A) (Kuberski and Szumna, 2009). Cavitand L-**PheR** has a zwitterionic character, and in a non-polar environment it quantitatively self-assembles to dimeric capsules (L-**PheR**)_2_ using complementary, charged hydrogen bonding interactions (Figure 1B). (L-**PheR**)_2_ resembles a molecular “reversed micelle” with a hydrophobic outside core and a polar internal cavity (Szumna, 2009a; Szumna 2009b). The binding motif that is based on electrostatic interactions can be also effective in water (Beyeh et al., 2018). Therefore, we attempted to obtain capsules using polar amino acids (Glu, Asp, Arg, and His) and resorcinarenes with hydrophilic feet (Figure 1C), which, as we assumed, would render the cavitands/capsules water-soluble and water-stable characters.

Synthesis involves the Mannich reaction between polar amino acids resorcin[4]arene **R** and formaldehyde (Figure 1C). Among the amino acids tested (Glu, Asp, Arg, and His), only glutamic acid and arginine gave the cavitands (L-**GluR**, D-**GluR**, and L-**ArgR**). Nevertheless, the ESI MS spectrum of L-**GluR** shows the signal of (M-4H2O – H]^–^ (Supplementary Figure S66), and in the HMBC spectrum, we found an additional low-intensity correlation between H_g_ and C_k_ (Supplementary Figure S4), which suggests that L-**GluR**, in contrary to L-**ArgR**, underwent intramolecular cyclization (Figure 1C). L-**GluR** was obtained with 50% yield by the reaction in DMF/water in a ratio of 1:3 and purification performed by a washing procedure. L-**ArgR** was obtained with 47% yield by the reaction in MeOH/water in a ratio of 1:1 at pH 3.5 and purification performed by SEC chromatography. Cavitand **PhR** (Setner and Szumna, 2019), which is based on 2-phenylethylamine and lacks a complementary hydrogen-bonding pattern, was also synthesized as a reference.

In contrast to glutamic acid and arginine, aspartic acid and histidine gave complex mixtures of products in the Mannich reaction with resorcin[4]arene **R** and formaldehyde under various tested conditions (methanol, DMF, water, and their mixtures, with or without the addition of acetic acid). The formation of such mixtures was rationalized by the complex reactivity patterns of functional amino acids with formaldehyde (Kamps et al., 2019), which involved reversible (e.g., *N*-substitutions with hydroxymethyl groups) and irreversible (e.g., CH-substitution in the imidazole ring of histidine) modifications of side chains and/or lack of self-assembly, that is, crucial “directing force” for the distribution of the products (Wierzbicki and Szumna, 2013). It should be noted that under acidic conditions partial or complete racemization was observed.

### 3.1.2 Self-Assembly of Homocapsules

The cavitands are expected to self-assemble to dimeric capsules using electrostatic interactions between zwitterionic structures involving their “backbone groups” (for L-**ArgR**, Figure 1B; Supplementary Figure S69) or by hydrophobic clustering (for L-**GluR**, Supplementary Figure S68). Thus, self-assembly is expected to be crucially dependent on the solvent, concentration, and pH.

**FIGURE 2:**
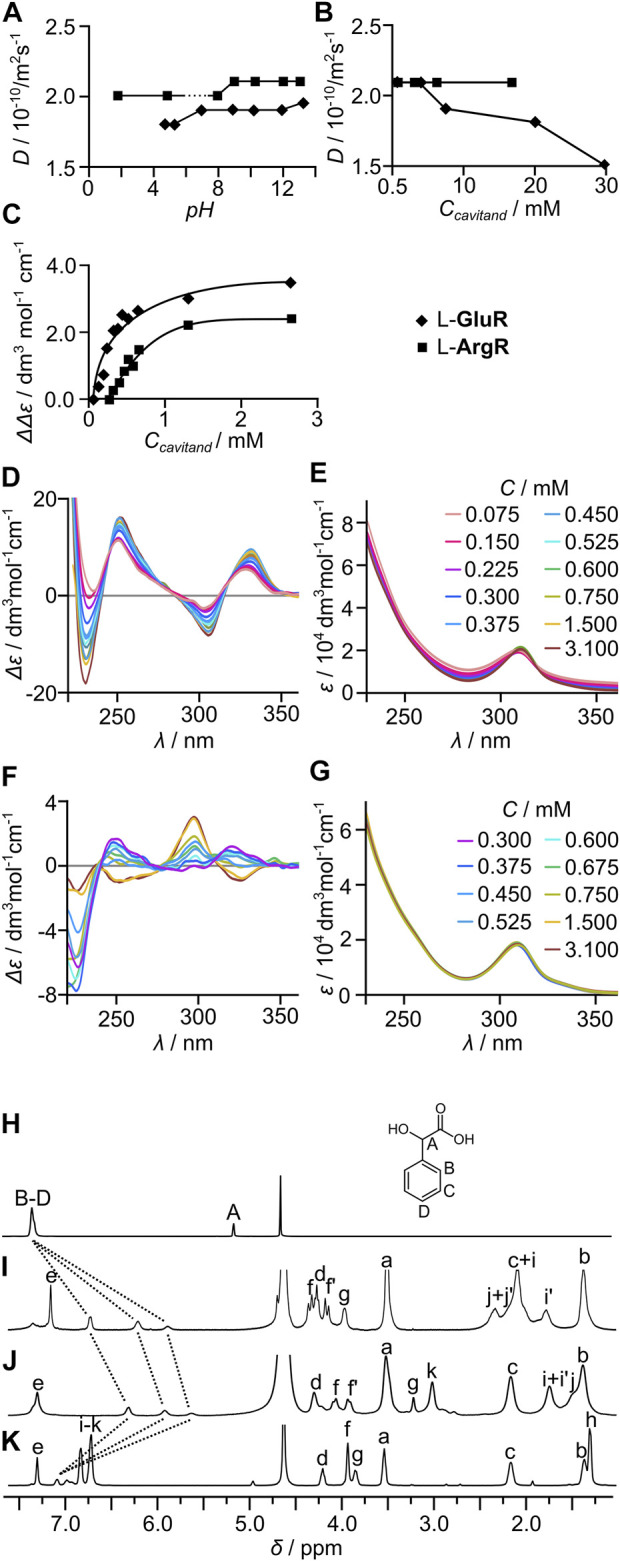
Self-assembly of homocapsules (L-**GluR**)_2_ and (L-**ArgR**)_2_ in water. Diffusion coefficients (*D*, measured by DOSY) of (L-**GluR**)_2_ and (L-**ArgR**)_2_ in water: **(A)** at various pH (C = 10 mM); and **(B)** at various concentrations (pH = 4.8). Concentration-dependent CD and UV spectra: **(C)** a plot of concentration-dependent intensity of CD bands for L-**GluR** and L-**ArgR** (water, pH = 11.9); **(D)** CD for L-**GluR**; **(E)** UV for L-**GluR**; **(F)** CD for L-**ArgR**; and **(G)** UV for L-**ArgR**. The encapsulation of mandelic acid (*R*)-**3** followed by ^1^H NMR: **(H)** (*R*)-3; **(I)** (L-**GluR**)_2_ + (*R*)-**3**; **(J)** (L-**ArgR**)_2_ + (*R*)-**3**; **(K) PhR**+ (*R*)-**3** (*C*_(**3**)_ 10 mM, *C*_(cavitand)_ 20 mM, D_2_O, pH = 4.8, 600 MHz, 298 K).

The diffusion coefficient (*D*, measured by DOSY) of L-**GluR** in water is 2.1 × 10^−10^ m^2^s^−1^ (at pH 4.8, 5 mM), which corresponds to *r*_H_ = 11.6 Å (calculated using the Stokes–Einstein equation for spherical particles, see ESI for details). This value is in reasonable agreement with the estimated *r*_H_ for a dimeric (L-**GluR**)_2_ [*r*_H_ (dimer) = 9.1 Å, *r*_H_ (cavitand) = 7.2 Å calculated by averaging the dimensions of the model structure, Supplementary Figures S68 and S69]. The values of *D* remain constant over the range of pH 4–13 (Figure 2A, for pH < 4, the compound is not soluble) indicating that (L-**GluR**)_2_ forms within a wide pH range. Concentration-dependent (L-**GluR**)_2_ (0.74–30 mM) indicates that the values of *D* reflect the size of dimeric species within the concentration range of 0.74–10 mM, and at a higher concentration the compounds start to aggregate (Figure 2B). To follow the process at lower concentrations, we used CD spectroscopy, which can be a sensitive probe for self-assembly (Szymański et al., 2021). Concentration-dependent CD spectra of L-**GluR** (0.075–3.1 mM) show a gradual decrease of intensity of the bands (Δε), while the intensities of the UV bands (ε) remain unchanged (Figures 2C–E). This trend is interpreted by gradual dissociation of the dimers to monomers at concentration < 1 mM. The explanation of this effect is based on the previous findings that correlate conformational lability and self-assembly processes with the intensity of ECD spectra (Szymański et al., 2021). Monomers are expected to have higher conformational lability than the self-assembled dimers. The ECD effects (Δε), in contrary to molar absorptivity (ε), are highly conformation-dependent; therefore, averaging many substantially different spectra of conformers of a labile molecule lowers the intensity of the ECD spectrum in comparison to the conformationally rigid analog. Self-assembly restricts conformational lability, and thus the ECD spectrum of (L-**GluR**)_2_ should have a higher intensity than the spectrum of the conformationally labile monomer L-**GluR**.

Furthermore, the proof of capsule formation in water comes from the complexation of mandelic acid, **3**. The addition of **3** (1 equiv.) to (L-**GluR**)_2_ results in a substantial upfield chemical shift of the guest’s signals consistent with the complexation of the guest within the cavity (Figures 2H,I). Effective complexation is observed over the wide pH range (Supplementary Figure S38). The addition of an excess amount of **3** (5 equiv.) to (L-**GluR**)_2_ leads to a gradual decrease of complexation-induced shifts (CISs), indicating that complexation/decomplexation processes are fast on the NMR timescale (milliseconds or faster). This fast kinetics of encapsulation in water for (L-**GluR**)_2_ is in sharp contrast with the previously observed behavior of the hydrophobic capsule (L-**PheR**)_2_, that is, in non-polar solvents, it exhibits very slow kinetics of guest complexation (in the range of days). Control experiments with cavitand **PhR** (which is monomeric but still can complex the guest in the cavity) and **3** demonstrate that **PhR** exerts considerably smaller influence on the chemical shifts of **3** (Figures 2H,K).

L-**ArgR** shows an analogous self-assembly behavior in water to L-**GluR**, with differences originating from different solubility (L-**ArgR** is insoluble in the pH of 6–8). In the remaining pH range and within the concentration range of 0.74–20.0 mM, diffusion coefficients remain similar corresponding to the size of capsules (L-**ArgR**)_2_ (*D* = 2.1 × 10^−10^ m^2^s^−1^ r_H_ = 11.6 Å, Figure 2B). The concentration-dependent CD spectra (3.1–0.20 mM) show a gradual decrease of (Δε) upon dilution (Figures 2C,F,G). Complexation experiments also show the encapsulation of mandelic acid, **3**, in the cavity of (L-**ArgR**)_2_ at different pH (Figures 2H,J).

In agreement with the hydrophobic character interactions, L-**GluR** does not self-assemble in DMSO. It is manifested by diffusion coefficient *D* = 1.5 × 10^−10^ m^2^s^−1^ that corresponds to *r*_H_ = 7.4 Å. The addition of **3** (1 equiv.) to L-**GluR** in DMSO causes no changes in the chemical shifts of the guest, indicating no interactions in this solvent (Supplementary Figure S36).

### 3.1.3 Protonation Equilibria

Even though the dimers remain stable over the wide pH range, as it was demonstrated in Figure 2A, protonation equilibria influence their structures, due to the presence of various pH-dependent groups (amine, guanidine, carboxylic, and phenolic groups) in different structural parts (backbones and side chains). The influence of pH on the structure of dimers was followed by UV, ECD, and NMR.

**FIGURE 3:**
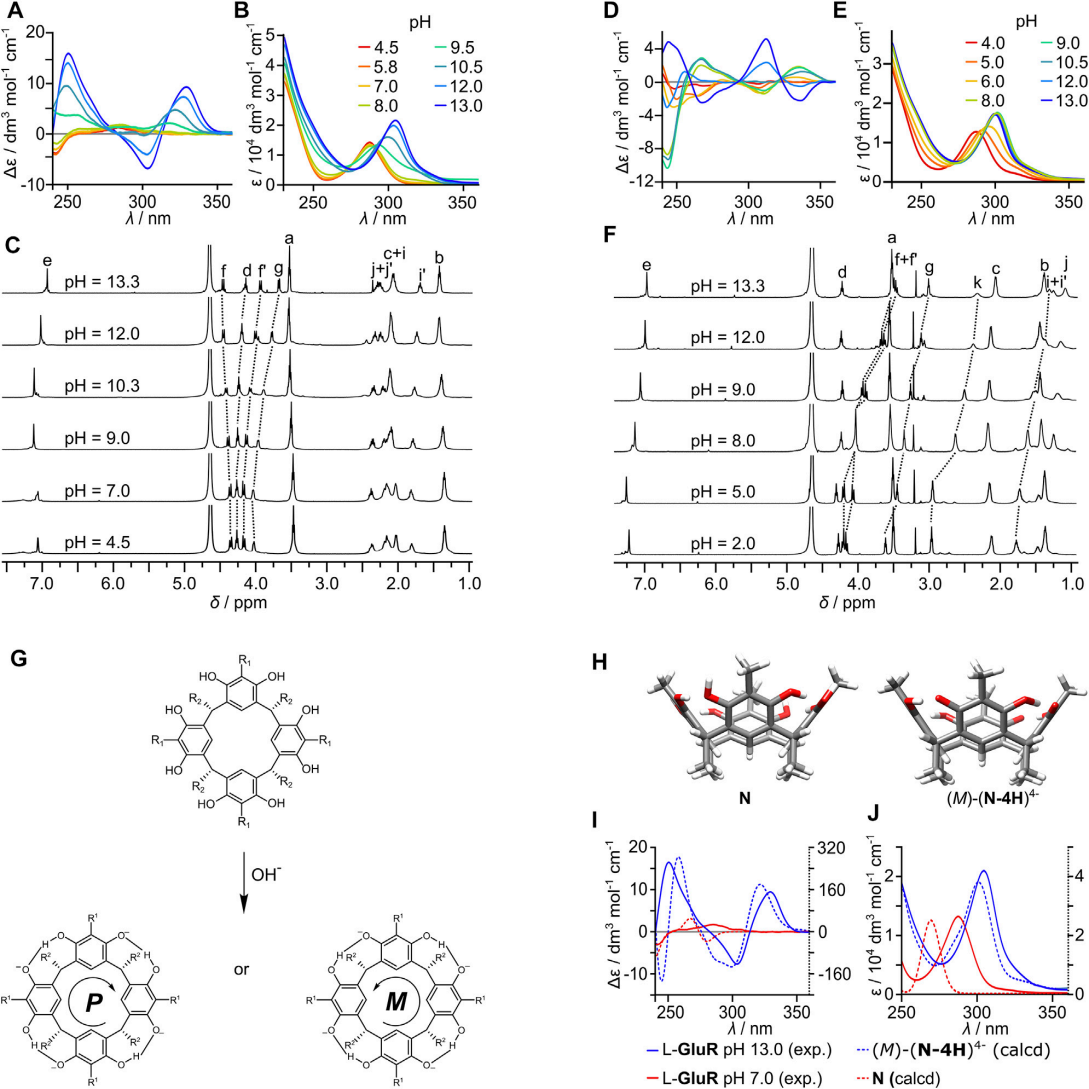
Structural changes of homodimers upon pH change: **(A,B)** pH-dependent ECD and UV spectra of L-**GluR** (C = 3.1 mM, water, pH was adjusted by using NaOH); **(C)** pH-dependent changes of NMR spectra of (L-**GluR**)_2_ (C = 10 mM, water, pH was adjusted by using NaOD); **(D,E)** pH-dependent ECD and UV spectra of L-**ArgR** (C = 3.1 mM, water, pH was adjusted by using NaOH); **(F)** pH-dependent ^1^H NMR spectra of (L-**ArgR**)_2_ (C = 10 mM, water, pH was adjusted by using NaOD); **(G)** postulated mechanism of diastereoselective deprotonation; **(H)** geometry-optimized structures of model compounds: neutral **(N)** in *C*_4_ inherently chiral conformation and tetraanionic, (*M*)-(**N-4H**)^**4−**^; comparison of calculated and experimental spectra: **(I)** CD; **(J)** UV (TD DFT wb97xd/6-31+G(d); y-axis: left, solid axis—experimental intensity; right, dashed axis—calculated intensity).

For (L-**GluR**)_2_, the band at 290 nm in the UV spectrum remains unchanged up to a pH of 9.0, and then it shows the abrupt bathochromic shift to 305 nm (Figure 3B), indicating deprotonation of phenolic groups in agreement with resorcinol’s pKa1 = 9.30. Furthermore, the increase of pH (up to 13) does not induce substantial changes, which may indicate that the second resorcinol deprotonation event (for resorcinol pKa2 = 11.06) is not visible at UV spectra of (L-**GluR**)_2_. A similar trend is observed for (L-**ArgR**)_2_, but deprotonation of phenolic groups takes place at a lower pH (Figure 3E). The deprotonation processes for both capsules are accompanied by large changes in CD signals (Figures 3A,D). For (L-**GluR**)_2_ at a pH of less than 9, the 250–350 nm bands are CD-silent, while the band at 240 nm shows a low-intensity negative CD effect. At a pH greater than 9, a positive couplet at 330/300 nm starts to emerge, and its intensity increases with the pH. For (L-**ArgR)**_**2**_, the pH-induced changes follow the same trajectory as for (L-**GluR**)_2_, but the corresponding changes are observed at lower pH (maximum at pH 8). The pronounced quantitative difference between (L-**ArgR**)_2_ and (L-**GluR**)_2_ appears at pH > 12, when the signs of CD signals reverse but only for (L-**ArgR**)_2_. This change may be attributed to the second deprotonation event of resorcinol rings that takes place for basic L-**ArgR**, while acidic L-**GluR** is not reaching this point within the studied pH range. It is interesting to note that deprotonation of a phenol group does not preclude the formation of a binding motif that stabilizes the capsule.

The enhancement of CD bands follows the first deprotonation event of resorcinol, which was also detected by the UV spectra. We attribute these changes to “diastereoselective deprotonation” of the resorcinol rings, that is, one of the hydroxyl groups in each resorcinol ring is selectively deprotonated and forms a *C*_4_-symmetric inherently chiral OH⋅⋅O^−^ pattern (Figure 3G). This hypothesis is consistent with the NMR results that show increasing differentiation of diastereotopic H_f_ protons at high pH (Figure 3C). It is further supported by the calculation of theoretical CD spectra for model compounds: neutral (**N**) and tetraanionic (**N-4H**)^4−^ (Figure 3H) by TD DFT wb97xd/6-31+G(d) (Frisch et al., 2016). The results show that neutral form, **N**, possessing eight OH groups arranged in a *C*_4_-symmetric chiral conformation has very small CD effects within the resorcinol chromophore (Figure 3I). Upon tetra-deprotonation and formation of a *C*_4_-symmetric inherently chiral OH⋅⋅O^−^ pattern, like in the (*M*)-(**R-4H**)^4−^, the CD effects become an order of magnitude larger, supporting the hypothesis that diastereoselective deprotonation is responsible for an increase of CD intensity at high pH. Thus, for (L-**ArgR**)_2_ and (L-**GluR**)_2_, the chirality of the amino acids induces directional deprotonation, which is effective only in the self-assembled dimers, and decreases as the dimer dissociates (Figure 2C).

### 3.1.4 Self-Assembly of Heterocapsules

**FIGURE 4:**
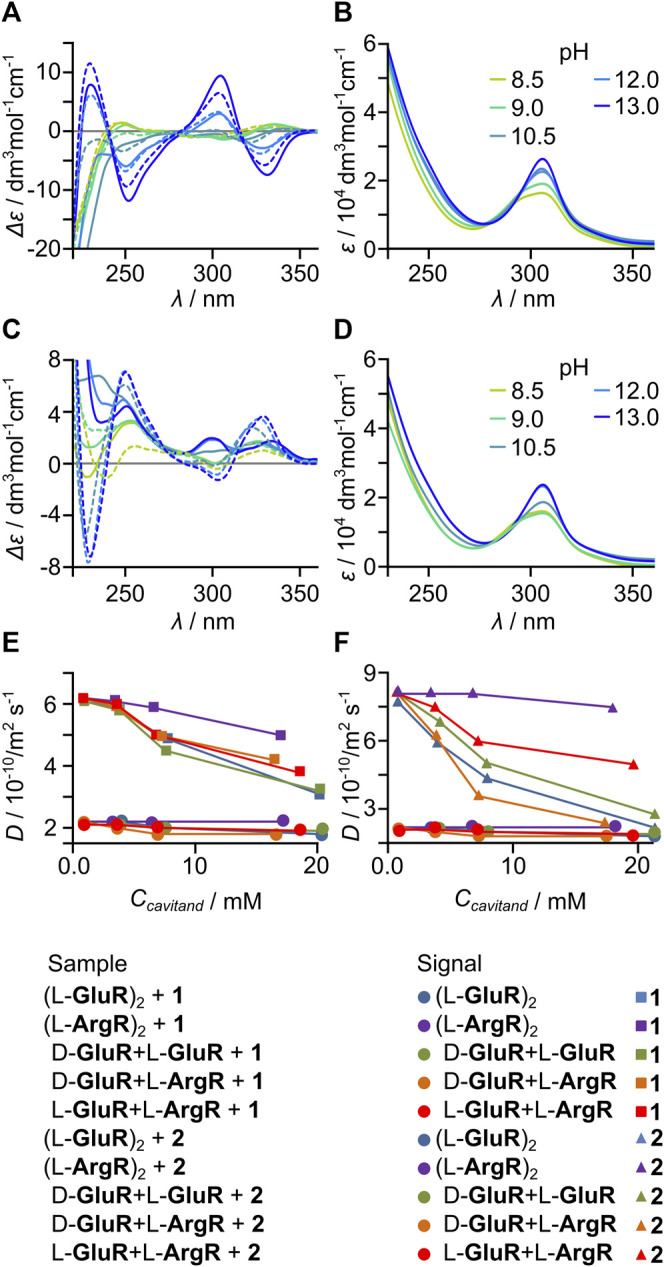
Formation of heterodimers: **(A)** pH-dependent ECD, and **(B)** UV spectra of D-**GluR** + L-**ArgR**; **(C)** pH-dependent ECD and **(D)** UV spectra of L-**GluR** + L-**ArgR** (solid lines—experimental spectra and dashed lines—a weighted mathematical sum of the components at given pH). Diffusion coefficient changes (capsules’ and guests’ signals) during titration of **(E) 1** and **(F) 2**, with various capsules (pH = 9, C_(guest)_ = 10 mM).

The acid/base character of the side chains and chirality of the cavitands enables the formation of heterodimers involving either acid–base dimers or D–L dimers. It can be expected that favorable electrostatic interactions between side chains of positively charged arginine and negatively charged glutamic acid may favor the formation of heterodimers. On the other hand, chirality may also play a role because in a head-to-head dimer the steric repulsion between side chains should be smaller for D–L dimers than for L–L dimers (Jędrzejewska and Szumna, 2017). To test these possibilities, we performed experiments of heterodimer formation for D-**GluR** + L-**GluR**, L-**GluR** + L**-ArgR**, and D-**GluR** + L**-ArgR**. Indeed, when the cavitands containing arginine were mixed with those containing glutamic acid in methanol or water at pH < 7, the formation of precipitates was observed independently of the chirality of the components, indicating the effective formation of heterodimers. For mixtures containing only glutamic acid cavitands but of different chirality, that is, D-**GluR** + L-**GluR,** no precipitation was observed. It was found that precipitates have different solubility profiles than the components, for example, they do not dissolve in DMSO but dissolve in water only at pH ≥ 9. After dissolution of the precipitates in water at pH = 9, it was found that they contain equimolar mixtures of cavitands **ArgR** and **GluR** (the supernatants contain only traces of the substrates). The D-**GluR** + L-**ArgR** and L-**GluR** + L**-ArgR** heterodimers remain in fast exchange with their components on the NMR timescale, and the spectra are broad. Evidence for the formation of heterodimers was found by analyzing ECD spectra and complexation characteristics toward benzaldehyde **2** (Figure 4).

Experimental ECD spectra of acid–base heterodimers (D-**GluR** + L**-ArgR** and L-**GluR** + L**-ArgR** are different than the sum components, especially at pH > 10 (Figures 4A,C). Different behaviors of the mixtures were also observed during titrations of **2** (10 mM) with varying amounts of capsules (3–10 mM in respect to cavitand, pH 9) monitored by DOSY (Figure 4F). D-**GluR** + L**-ArgR** and L-**GluR** + L-**ArgR** induce a decrease of *D* values of **2** more effectively than it is expected for the sum of components. This indirectly proves the formation of heterodimers which have better encapsulation properties than the components. The formation of heterochiral dimers D-**GluR** + L-**GluR** remains ambiguous.

### 3.1.5 Screening of Encapsulation Properties

Assuming that the binding motif of hydrophilic capsules is similar to that of hydrophobic (L-**PheR**)_2_, in case of (L-**ArgR**)_2_ (Kuberski and Szumna, 2009) or based on hydrophobic clustering, in case of (L-**GluR**)_2_, the size of the cavity can be estimated as c.a. 300 Å^3^. Even after having these initial hints, it is non-trivial to predict complexation preferences of the capsules in water because an aqueous environment diminishes polar interactions while enhancing hydrophobic ones (Escobar and Ballester, 2021). Additionally, due to the dynamic character of the capsules in water, the size of cavities may be adaptable.

We have found that purely hydrophobic guests, for example, toluene and cyclohexane are not complexed in the cavity. This is in contrast to previously reported water-soluble capsules known from the literature that typically prefer encapsulation of hydrophobic guests by hydrophobic effects. Highly hydrophilic guests, for example, glucose or mannitol (known to interact with resorcinarenes in a non-polar environment) (Kikuchi et al., 1992; Evan-Salem et al., 2006) are also not complexed. Efficient complexation was observed for guests with mixed polar/apolar characters that are compatible with the character of the cavity (Figure 1E). Neutral guests such as epoxides, esters, ketones, aldehydes, and amides are effectively complexed inside (L-**GluR**)_2_. Charged guests such as acids and amines, carnitine, tetraalkylammonium salts, and some amino acids also experience considerable upfield shifts upon interactions with (L-**GluR**)_2_ (Supplementary Figures S32–S53). Control experiments show no upfield shift for neutral guests upon addition of control cavitand **PhR**, indicating that the observed effects for complexation in (L-**GluR**)_2_ can be attributed to encapsulation.

**FIGURE 5:**
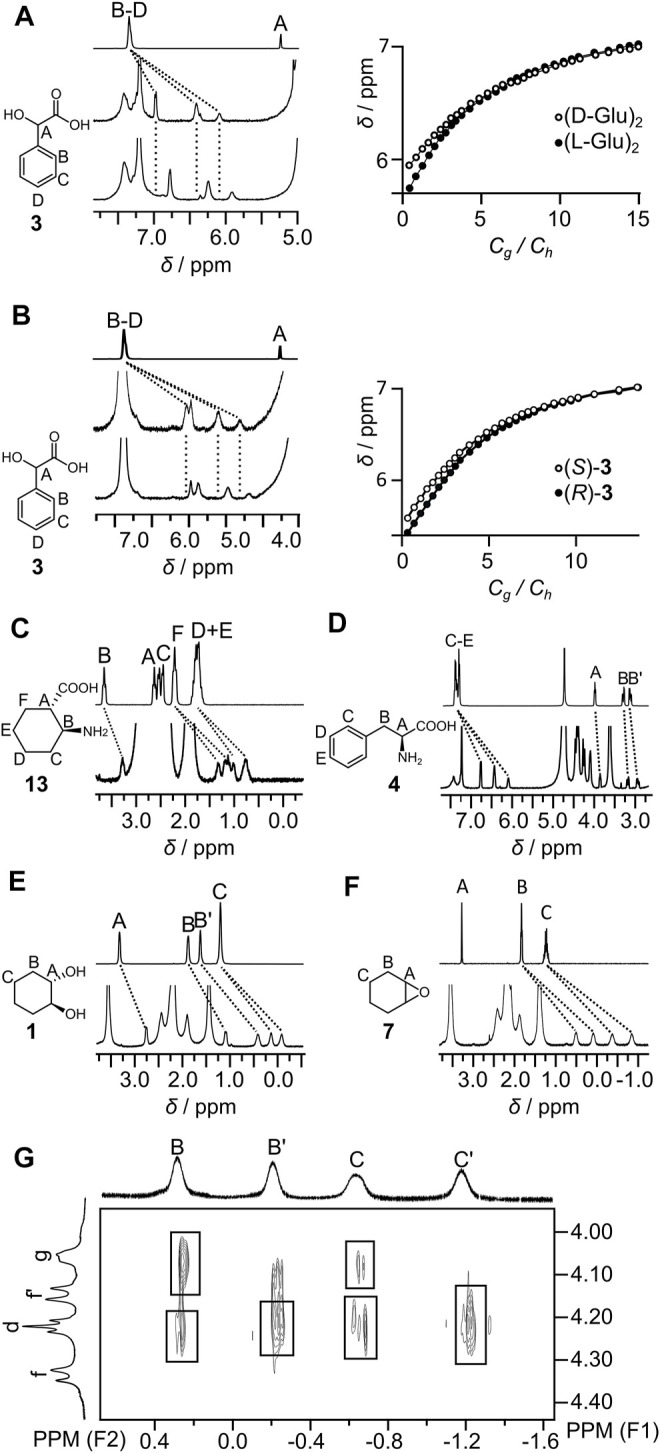
Complexation properties of the capsules (partial ^1^H NMR spectra and selected titration curves): **(A) 3** (top) and (*S*)-**3** with (L-**GluR**)_2_ (middle) and with (D-**GluR**)_2_ (bottom); **(B) 3** (top) and (*S*)-**3** with (L-**ArgR**)_2_ (middle) and (*R*)-**3** (L-**ArgR**)_2_ (bottom); **(C)** (L-**GluR**)_2_ with (1*S*, 2*R*)-**13**; **(D)** (L-**GluR**)_2_ with (*S*)-**4**; **(E)** (L-**GluR**)_2_ with (1*S*, 2*S*)-**1**; **(F)** (L-**GluR**)_2_ with **7**; **(G)** partial ROESY spectrum of (L-**GluR**)_2_ with **7** (D_2_O, 600 MHz).

Titration experiments have been performed for representative guests. Titration curves could not be fitted assuming a 1:1 H:G model and may reflect the influence of the guest on capsule’s self-assembly and/or complex formation with more than one guest inside the cavity. The Job plot for representative guest **3** (Supplementary Figure S33) suggests mixed stoichiometry with a dominant 1:1 complex and a lower amount of the complex containing more than one guest molecule. Good quality fits to the titration data were obtained assuming the formation of two types of species: (guest)⊂(L-**GluR**)_2_ (K_1_) and (guest)_2_⊂(L-**GluR**)_2_ (K_2_) (Supplementary Figures S32, S35, S37, S49, S50). Data show that (L-**GluR**)_2_ exhibits an affinity for the studied guests in the range of logK_1_ = 3.9–5.0 and contributions of the complexes with two guests in the range of logK_2_ = 1.3–2.0 (Table 1). The experimentally determined K_2_ values are considerably lower than the statistically predicted K_2(stat)_ = K_1_/4. This effect is attributed to negative cooperativity due to repulsions in the cavity. The chiral recognition of the enantiomers was moderate, with the largest difference observed between (*R*)-**3** and (*S*)-**3**. Qualitative chiral recognition of all chiral guests was also observed in ^1^H NMR spectra (Figures 5A,B), indicating different binding modes between enantiomeric guests and chiral capsules. In most cases, determination of the exact mode of interactions by NOE/ROE was not possible due to the broadening of the signals as a result of in–out exchange and tumbling of the guests inside the cavity. However, in the case of guest **7**, the ROESY spectra clearly show ROEs between the signals of encapsulated **7** and (L-**GluR**)_2_ (Figure 5H). The *C′* signal of the guest (which is the most shifted) exhibits the strongest interactions with the *d* protons of the capsule (positioned at the bottom of the cavity), indicating that the C protons are positioned deep in the cavity. The less-shifted signals of the guest, *B*, exhibit the strongest interactions with *g* protons of the capsule, which are positioned in the central part of the cavity. Such an orientation of guest **7** is in agreement with the amphiphilic character of the cavity; the hydrophobic poles interact preferably with the aliphatic part of the guest molecule, and the central hydrophilic part prefers to host the polar part of guest **7**.

**TABLE 1:** Complexation constants determined by ^1^H NMR titrations and subsequent curve fitting using HypNMR (D_2_O, pH = 4.8, 298 K).

Host (H)	Guest (G)	logK_1_ ^a^	σ_1_ ^c^	logK_2_ ^b^	σ_2_ ^c^
(D-**GluR**)_2_	(*R*)**-3**	4.85	0.02	1.25	0.02
(L-**GluR**)_2_	(*R*)**-3**	4.20	0.03	1.80	0.03
(D-**GluR**)_2_	**1**	4.84	0.03	1.85	0.03
(L-(**GluR**)_2_	**1**	4.81	0.04	1.73	0.03
(D-**GluR**)_2_	**13**	4.80	0.02	1.46	0.02
(L-(**GluR**)_2_	**13**	4.80	0.05	1.32	0.05
(D-**GluR**)_2_	**16**	4.77	0.03	1.54	0.03
(L-(**GluR**)_2_	**16**	4.99	0.02	1.43	0.02
(L-**ArgR**)_2_	(*R*)**-3**	4.78	0.02	1.85	0.02
(L-**ArgR**)_2_	(*S*)**-3**	4.34	0.03	1.77	0.03

a K_1_ = [HG]/[H][G].

b K_2_ = [HG_2_]/[HG][G].

c σ—standard deviation.

## 3.2 Conclusion

We have presented the synthesis of new chiral dimeric capsules made of polar amino acids and demonstrated their self-assembly in the most demanding aqueous environment. The capsules have functional interiors and encapsulate a variety of guests that are compatible with the mixed hydrophobic/hydrophilic character of their cavities. The assembly/disassembly and complexation processes are fast on the NMR timescale. These features make the reported capsules markedly different from previously known water-soluble capsules (typically achiral and encapsulating hydrophobic guests) and from structurally analogous hydrophobic capsules that exhibit very slow complexation rates. These features are also desirable for future applications, for example, in catalysis, which typically requires fast kinetics, moderate complexation strength, and stabilization of chiral polar transition states. Moreover, the possibilities of using longer peptides open the way toward the extension of the capsules. These possibilities are currently being tested.

## Data Availability

The original contributions presented in the study are included in the article/Supplementary Material, further inquiries can be directed to the corresponding author.

## References

[B1] Adriaenssens L. Ballester P. (2013). Hydrogen-Bonded Supramolecular Capsules with Functionalized Interiors: the Controlled Orientation of Included Guests. Chem. Soc. Rev. 42, 3261–3277. 10.1039/c2cs35461f 23321897

[B2] Ashbaugh H. S. Gibb B. C. Suating P. (2021). Cavitand Complexes in Aqueous Solution: Collaborative Experimental and Computational Studies of the Wetting, Assembly, and Function of Nanoscopic Bowls in Water. J. Phys. Chem. B 125, 3253–3268. 10.1021/acs.jpcb.0c11017 33651614 PMC8040017

[B3] Ayhan M. M. Casano G. Karoui H. Rockenbauer A. Monnier V. Hardy M. (2015). EPR Studies of the Binding Properties, Guest Dynamics, and Inner-Space Dimensions of a Water-Soluble Resorcinarene Capsule. Chem. Eur. J. 21, 16404–16410. 10.1002/chem.201502212 26403999

[B4] Beaudoin D. Rominger F. Mastalerz M. (2016). Chirality-Assisted Synthesis of a Very Large Octameric Hydrogen-Bonded Capsule. Angew. Chem. Int. Ed. 55, 15599–15603. 10.1002/anie.201609073 27862733

[B5] Beyeh N. K. Díez I. Taimoory S. M. Meister D. Feig A. I. Trant J. F. (2018). High-Affinity and Selective Detection of Pyrophosphate in Water by a Resorcinarene Salt Receptor. Chem. Sci. 9, 1358–1367. 10.1039/c7sc05167k 29675184 PMC5887233

[B6] Bolliger J. L. Belenguer A. M. Nitschke J. R. (2013). Enantiopure Water-Soluble [Fe4L6] Cages: Host-Guest Chemistry and Catalytic Activity. Angew. Chem. Int. Ed. 52, 7958–7962. 10.1002/anie.201302136 23788518

[B7] Brown C. J. Toste F. D. Bergman R. G. Raymond K. N. (2015). Supramolecular Catalysis in Metal-Ligand Cluster Hosts. Chem. Rev. 115, 3012–3035. 10.1021/cr4001226 25898212

[B8] Butler R. N. Coyne A. G. (2010). Water: Nature's Reaction Enforcer-Comparative Effects for Organic Synthesis "In-Water" and "On-Water". Chem. Rev. 110, 6302–6337. 10.1021/cr100162c 20815348

[B9] Castilla A. M. Ramsay W. J. Nitschke J. R. (2014). Stereochemistry in Subcomponent Self-Assembly. Acc. Chem. Res. 47, 2063–2073. 10.1021/ar5000924 24793652

[B10] Chen L.-J. Yang H.-B. Shionoya M. (2017). Chiral Metallosupramolecular Architectures. Chem. Soc. Rev. 46, 2555–2576. 10.1039/c7cs00173h 28452389

[B11] Corbellini F. Knegtel R. M. A. Grootenhuis P. D. J. Crego-Calama M. Reinhoudt D. N. (2005). Water-Soluble Molecular Capsules: Self-Assembly and Binding Properties. Chem. Eur. J. 11, 298–307. 10.1002/chem.200400849 15551310

[B12] Cullen W. Turega S. Hunter C. A. Ward M. D. (2015). pH-Dependent Binding of Guests in the Cavity of a Polyhedral Coordination Cage: Reversible Uptake and Release of Drug Molecules. Chem. Sci. 6, 625–631. 10.1039/c4sc02090a 28936311 PMC5588781

[B13] Escobar L. Ballester P. (2021). Molecular Recognition in Water Using Macrocyclic Synthetic Receptors. Chem. Rev. 121, 2445–2514. 10.1021/acs.chemrev.0c00522 33472000

[B14] Evan-Salem T. Baruch I. Avram L. Cohen Y. Palmer L. C. Rebek J. Jr. (2006). Resorcinarenes Are Hexameric Capsules in Solution. Proc. Natl. Acad. Sci. U.S.A. 103, 12296–12300. 10.1073/pnas.0604757103 16894154 PMC1567874

[B15] Frassineti C. Alderighi L. Gans P. Sabatini A. Vacca A. Ghelli S. (2003). Determination of Protonation Constants of Some Fluorinated Polyamines by Means of 13C NMR Data Processed by the New Computer Program HypNMR2000. Protonation Sequence in Polyamines. Anal. Bioanal. Chem. 376, 1041–1052. 10.1007/s00216-003-2020-0 12845401

[B16] Frassineti C. Ghelli S. Gans P. Sabatini A. Moruzzi M. S. Vacca A. (1995). Nuclear Magnetic Resonance as a Tool for Determining Protonation Constants of Natural Polyprotic Bases in Solution. Anal. Biochem. 231, 374–382. 10.1006/abio.1995.9984 8594988

[B17] Frisch M. J. Trucks G. W. Schlegel H. B. Scuseria G. E. Robb M. A. Cheeseman J. R. (2016). Gaussian 09, Revision E.01. Wallingford CT: Gaussian, Inc.

[B18] Gawande M. B. Bonifácio V. D. B. Luque R. Branco P. S. Varma R. S. (2013). Benign by Design: Catalyst-free In-Water, On-Water Green Chemical Methodologies in Organic Synthesis. Chem. Soc. Rev. 42, 5522–5551. 10.1039/c3cs60025d 23529409

[B19] Gibb B. C. Chapman R. G. Sherman J. C. (1996). Synthesis of Hydroxyl-Footed Cavitands. J. Org. Chem. 61, 1505–1509. 10.1021/jo951633c

[B20] Gibb C. L. D. Gibb B. C. (2004). Well-Defined, Organic Nanoenvironments in Water: The Hydrophobic Effect Drives a Capsular Assembly. J. Am. Chem. Soc. 126, 11408–11409. 10.1021/ja0475611 15366865

[B21] Gropp C. Quigley B. L. Diederich F. (2018). Molecular Recognition with Resorcin[4]arene Cavitands: Switching, Halogen-Bonded Capsules, and Enantioselective Complexation. J. Am. Chem. Soc. 140, 2705–2717. 10.1021/jacs.7b12894 29451782

[B22] Guo H. Zhang L. W. Zhou H. Meng W. Ao Y. F. Wang D. X. (2020). Substrate-Induced Dimerization Assembly of Chiral Macrocycle Catalysts toward Cooperative Asymmetric Catalysis. Angew. Chem. Int. Ed. 59, 2623–2627. 10.1002/anie.201910399 31845448

[B23] Hanafusa M. Tsuchida Y. Matsumoto K. Kondo K. Yoshizawa M. (2020). Three Host Peculiarities of a Cycloalkane-Based Micelle toward Large Metal-Complex Guests. Nat. Commun. 11, 6061–6068. 10.1038/s41467-020-19886-4 33247106 PMC7695700

[B24] He Y.-P. Yuan L.-B. Song J.-S. Chen G.-H. Lin Q. Li C. (2018). Optical Resolution of the Water-Soluble Ti4(embonate)6 Cages for Enantioselective Recognition of Chiral Drugs. Chem. Mater. 30, 7769–7775. 10.1021/acs.chemmater.8b03174

[B25] Hiraoka S. Nakamura T. Shiro M. Shionoya M. (2010). In-Water Truly Monodisperse Aggregation of Gear-Shaped Amphiphiles Based on Hydrophobic Surface Engineering. J. Am. Chem. Soc. 132, 13223–13225. 10.1021/ja1069135 20815344

[B26] Jędrzejewska H. Szumna A. (2017). Making a Right or Left Choice: Chiral Self-Sorting as a Tool for the Formation of Discrete Complex Structures. Chem. Rev. 117, 4863–4899. 10.1021/acs.chemrev.6b00745 28277655

[B27] Jordan J. H. Gibb B. C. (2015). Molecular Containers Assembled through the Hydrophobic Effect. Chem. Soc. Rev. 44, 547–585. 10.1039/c4cs00191e 25088697

[B28] Kamps J. J. A. G. Hopkinson R. J. Schofield C. J. Claridge T. D. W. (2019). How Formaldehyde Reacts with Amino Acids. Commun. Chem. 2, 1–14. 10.1038/s42004-019-0224-2

[B29] Katagiri H. Tanaka Y. Furusho Y. Yashima E. (2007). Multicomponent Cylindrical Assemblies Driven by Amidinium-Carboxylate Salt-Bridge Formation. Angew. Chem. Int. Ed. 46, 2435–2439. 10.1002/anie.200603921 17309084

[B30] Kikuchi Y. Tanaka Y. Sutarto S. Kobayashi K. Toi H. Aoyama Y. (1992). Highly Cooperative Binding of Alkyl Glucopyranosides to the Resorcinol Cyclic Tetramer Due to Intracomplex Guest-Guest Hydrogen-Bonding: Solvophobicity/Solvophilicity Control by an Alkyl Group of the Geometry, Stoichiometry, Stereoselectivity, and Cooperativity. J. Am. Chem. Soc. 114, 10302–10306. 10.1021/ja00052a029

[B31] Kohlhaas M. Zähres M. Mayer C. Engeser M. Merten C. Niemeyer J. (2019). Chiral Hydrogen-Bonded Supramolecular Capsules: Synthesis, Characterization, and Complexation of C70. Chem. Commun. 55, 3298–3301. 10.1039/c8cc10152c 30810550

[B32] Kondo K. Suzuki A. Akita M. Yoshizawa M. (2013). Micelle-Like Molecular Capsules with Anthracene Shells as Photoactive Hosts. Angew. Chem. Int. Ed. 52, 2308–2312. 10.1002/anie.201208643 23345251

[B33] Kondo K. Klosterman J. K. Yoshizawa M. (2017). Aromatic Micelles as a New Class of Aqueous Molecular Flasks. Chem. Eur. J. 23, 16710–16721. 10.1002/chem.201702519 28710788

[B34] Kuberski B. Szumna A. (2009). A Self-Assembled Chiral Capsule with Polar Interior, Chem. Commun. 1959-1961. 10.1039/b820990a 19333456

[B35] Lipshutz B. H. Ghorai S. (2014). Transitioning Organic Synthesis from Organic Solvents to Water. What's *Your* E Factor? Green. Chem. 16, 3660–3679. 10.1039/c4gc00503a 25170307 PMC4142526

[B36] Liu M. Zhang L. Wang T. (2015). Supramolecular Chirality in Self-Assembled Systems. Chem. Rev. 115, 7304–7397. 10.1021/cr500671p 26189453

[B37] Martin A. D. Boulos R. A. Hubble L. J. Hartlieb K. J. Raston C. L. (2011). Multifunctional Water-Soluble Molecular Capsules Based on P-Phosphonic Acid Calix[5]arene. Chem. Commun. 47, 7353–7355. 10.1039/c1cc11991e 21637889

[B38] Mateos-Timoneda M. A. Crego-Calama M. Reinhoudt D. N. (2004). Supramolecular Chirality of Self-Assembled Systems in Solution. Chem. Soc. Rev. 33, 363–372. 10.1039/b305550g 15280969

[B39] Nie S.-X. Guo H. Huang T.-Y. Ao Y.-F. Wang D.-X. Wang Q.-Q. (2020). Xenon Binding by a Tight yet Adaptive Chiral Soft Capsule. Nat. Commun. 11, 6257. 10.1038/s41467-020-20081-8 33288758 PMC7721739

[B40] Ning R. Zhou H. Nie S. X. Ao Y. F. Wang D. X. Wang Q. Q. (2020). Chiral Macrocycle-Enabled Counteranion Trapping for Boosting Highly Efficient and Enantioselective Catalysis. Angew. Chem. Int. Ed. 59, 10894–10898. 10.1002/anie.202003673 32198953

[B41] Percástegui E. G. Ronson T. K. Nitschke J. R. (2020). Design and Applications of Water-Soluble Coordination Cages. Chem. Rev. 120, 13480–13544. 10.1021/acs.chemrev.0c00672 33238092 PMC7760102

[B42] Rahman F.-U. Tzeli D. Petsalakis I. D. Theodorakopoulos G. Ballester P. Rebek J. Jr. (2020). Chalcogen Bonding and Hydrophobic Effects Force Molecules into Small Spaces. J. Am. Chem. Soc. 142, 5876–5883. 10.1021/jacs.0c01290 32125842

[B43] Rivera J. M. Martín T. Rebek J. (2001). Chiral Softballs: Synthesis and Molecular Recognition Properties. J. Am. Chem. Soc. 123, 5213–5220. 10.1021/ja004080i 11457383

[B44] Rivera J. M. Martín T. Rebek J. (1998). Chiral Spaces: Dissymmetric Capsules through Self-Assembly. Science 279, 1021–1023. 10.1126/science.279.5353.1021 9461432

[B45] Sansone F. Baldini L. Casnati A. Chierici E. Faimani G. Ugozzoli F. (2004). Chiral Dimeric Capsules from N, C-Linked Peptidocalix[4]arenes Self-Assembled through an Antiparallel β-Sheetlike Motif. J. Am. Chem. Soc. 126, 6204–6205. 10.1021/ja031511z 15149197

[B46] Seeber G. Tiedemann B. E. F. Raymond K. N. (2006). “Supramolecular Chirality in Coordination Chemistry,” in Supramolecular Chirality (Berlin Springer), 147–183.

[B47] Setner B. Szumna A. (2019). Complexation of Chiral Amines by Resorcin[4]arene Sulfonic Acids in Polar media - Circular Dichroism and Diffusion Studies of Chirality Transfer and Solvent Dependence. Beilstein J. Org. Chem. 15, 1913–1924. 10.3762/bjoc.15.187 31501658 PMC6720235

[B48] Sheldon R. A. (2008). E Factors, Green Chemistry and Catalysis: an Odyssey. Chem. Commun. 3352-3365. 10.1039/b803584a 18633490

[B49] Sheldon R. A. (2016). Green Chemistry and Resource Efficiency: Towards a Green Economy. Green. Chem. 18, 3180–3183. 10.1039/c6gc90040b

[B50] Sheldon R. A. (2017). The E Factor 25 Years on: The Rise of Green Chemistry and Sustainability. Green. Chem. 19, 18–43. 10.1039/c6gc02157c

[B51] Simon M.-O. Li C.-J. (2012). Green Chemistry Oriented Organic Synthesis in Water. Chem. Soc. Rev. 41, 1415–1427. 10.1039/c1cs15222j 22048162

[B52] Suzuki A. Akita M. Yoshizawa M. (2016). Amphiphilic Tribranched Scaffolds with Polyaromatic Panels that Wrap Perylene Stacks Displaying Unusual Emissions. Chem. Commun. 52, 10024–10027. 10.1039/c6cc04823d 27444246

[B53] Suzuki A. Kondo K. Akita M. Yoshizawa M. (2013). Atroposelective Self-Assembly of a Molecular Capsule from Amphiphilic Anthracene Trimers. Angew. Chem. Int. Ed. 52, 8120–8123. 10.1002/anie.201302789 23784943

[B54] Szumna A. (2009a). Chiral Encapsulation by Directional Interactions. Chem. Eur. J. 15, 12381–12388. 10.1002/chem.200901654 19806617

[B55] Szumna A. (2009b). Water Co-encapsulation in an Inverted Molecular Capsule. Chem. Commun., 4191–4193. 10.1039/B908833D 19585017

[B56] Szymański M. P. Grajda M. Szumna A. (2021). Amplification of Electronic Circular Dichroism-A Tool to Follow Self-Assembly of Chiral Molecular Capsules. Molecules 26, 7100–7108. 10.3390/molecules26237100 34885682 PMC8658961

[B57] Taylor L. L. K. Riddell I. A. Smulders M. M. J. (2019). Self-Assembly of Functional Discrete Three-Dimensional Architectures in Water. Angew. Chem. Int. Ed. 58, 1280–1307. 10.1002/anie.201806297 29939463

[B58] Tiefenbacher K. Zhang K.-d. Ajami D. Rebek J. (2015). Robust Hydrogen-Bonded Capsules with Stability in Competitive Media. J. Phys. Org. Chem. 28, 187–190. 10.1002/poc.3378

[B59] Tokunaga Y. Rebek J. (1998). Chiral Capsules. 1. Softballs with Asymmetric Surfaces Bind Camphor Derivatives. J. Am. Chem. Soc. 120, 66–69. 10.1021/ja972885t

[B60] Watfa N. Xuan W. Sinclair Z. Pow R. Abul-Haija Y. Long D.-L. (2019). Enantioselective Recognition of Chiral Guests by the Water-Soluble Chiral Keplerate {Mo132} Spherical Capsule with 30 Inner Lactate Ligands. ChemRxiv, Cambridge. 10.26434/chemrxiv.8847923.v1

[B61] Wierzbicki M. Szumna A. (2013). Assembly-Driven Synthesis of Hybrid Molecular Capsules Controlled by Chiral Sorting. Chem. Commun. 49, 3860–3862. 10.1039/c3cc41515e 23545777

[B62] Yamashina M. Sei Y. Akita M. Yoshizawa M. (2014). Safe Storage of Radical Initiators within a Polyaromatic Nanocapsule. Nat. Commun. 5, 4662–4667. 10.1038/ncomms5662 25130933

[B63] Yi J. W. Barry N. P. E. Furrer M. A. Zava O. Dyson P. J. Therrien B. (2012). Delivery of Floxuridine Derivatives to Cancer Cells by Water-Soluble Organometallic Cages. Bioconjug. Chem. 23, 461–471. 10.1021/bc200472n 22263930

[B64] Zadmard R. Entezari H. Ataeian P. Mirza-Aghayan M. (2013). Water-Soluble Molecular Capsules via Multiple Ionic Interactions. Loc 10, 147–149. 10.2174/1570178611310020014

[B65] Zhan Y.-Y. Ogata K. Kojima T. Koide T. Ishii K. Mashiko T. (2018). Hyperthermostable Cube-Shaped Assembly in Water. Commun. Chem. 1, 1–9. 10.1038/s42004-018-0014-2

[B66] Zhang K.-D. Ajami D. Rebek J. (2013). Hydrogen-Bonded Capsules in Water. J. Am. Chem. Soc. 135, 18064–18066. 10.1021/ja410644p 24245649

