# Corrigendum: Chiral water-soluble molecular capsules with amphiphilic interiors

**DOI:** 10.3389/fchem.2023.1205654

**Published:** 2023-05-03

**Authors:** Arkadiusz Marek Sakowicz, Agnieszka Szumna

**Affiliations:** Institute of Organic Chemistry, Polish Academy of Sciences, Warsaw, Poland

**Keywords:** host–guest system, salt bridge, self-assembly, supramolecular chemistry, water chemistry

In the original article, there was an error in Figure 1 as published. Based on the re-analysis of published data and additional experiments, the authors claim that the molecular structure of L-**GluR** is cyclic. The corrected Figure 1 and its caption appear below.

**FIGURE 1:**
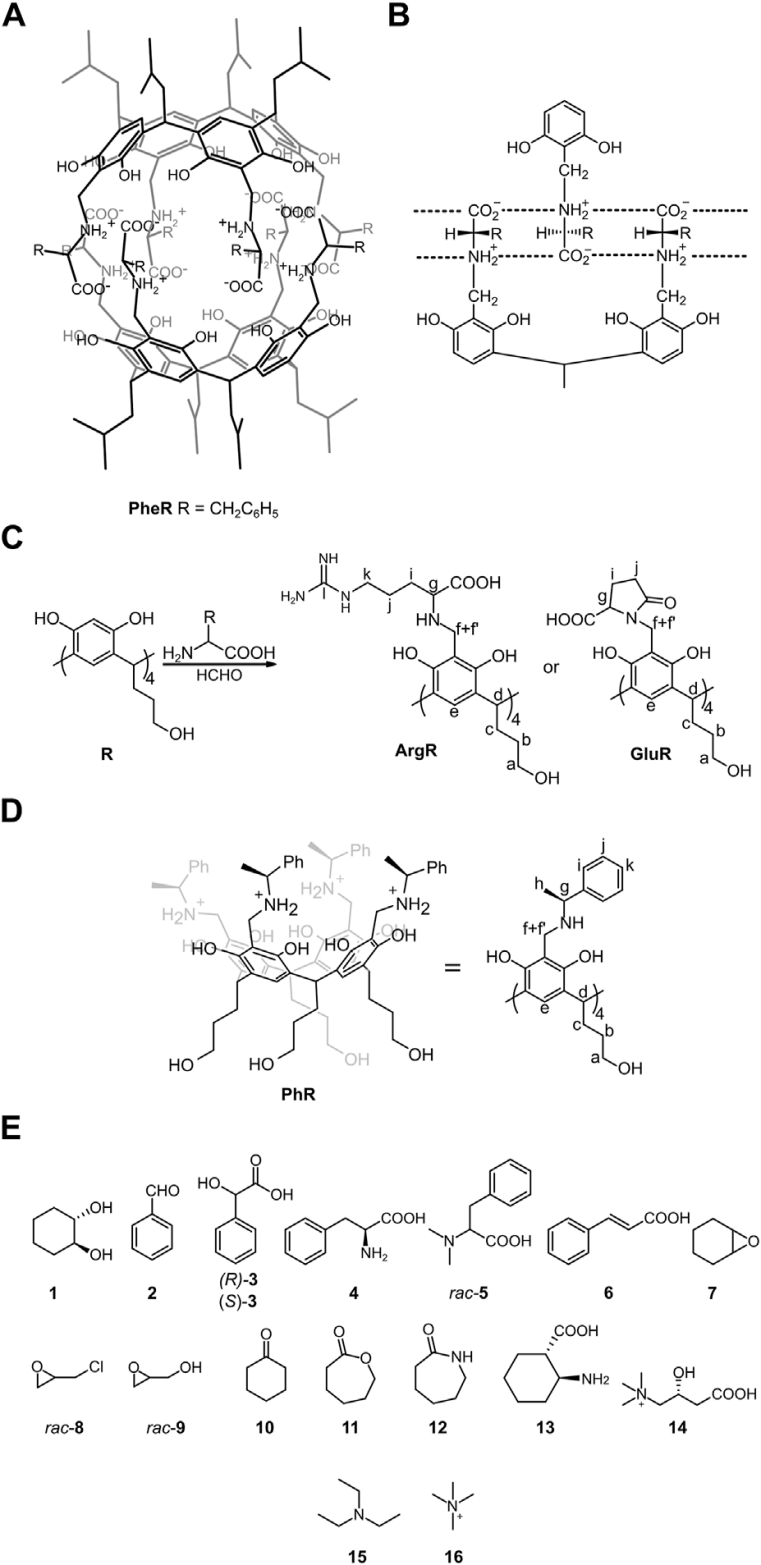
Structures of the compounds used in this study along with notation of the NMR signals: **(A,B)** hydrophobic capsule (L-**PheR**)_2_ and its binding motif (ref. Kuberski and Szumna, 2009); **(C)** synthesis of water-soluble cavitands; **(D)** structure of reference cavitand **PhR** (ref. Setner and Szumna, 2019); and **(E)** guest molecules.

In the original article, there were errors in the **Supplementary Figures S2, S4, S68, and S69**. The errors come from erroneous assignment of ^13^C NMR carbonyl signals (k,h) in the spectrum at pH 11 and in the cyclic structure of L-**GluR**. The correct figures can be viewed in the updated original article.

Continuing our studies on amphiphilic molecular capsules using dipeptides as components, we have found that dipeptides containing glutamic acid are prone to intramolecular cyclization. Therefore, we re-analyzed the published analytical data for (L-**GluR**)_2_ and have found similar spectral features indicating that intramolecular cyclization might have taken place for (L-**GluR**)_2_. Specifically, the ESI MS spectrum of L-**GluR** shows the signal of (M-4H_2_O-H)^−^ (**Supplementary Figure S66**). After re-interpretation of the HMBC spectrum and the re-assignment of carbonyl signals (h, k), we found an additional low-intensity correlation between H_g_ and C_k_. The ESI MS spectrum alone is not an unambiguous proof, because water loss in the gas phase is often observed for amino acid derivatives possessing carboxylic side chains, especially for large molecules that require high ionization energy in the electrospray technique (like here). However, together with the HMBC correlation and new data for dipeptides, it strongly suggest that L-**GluR** underwent intramolecular cyclization. Therefore, the reason for this correction is that the molecular structure of L-**GluR** is most likely cyclic. As claimed in the paper, formation of dimeric structures is still supported by DOSY, ECD and complexation studies for L-**GluR**. However, the mode of dimerization for L-**GluR** is most likely different than for (L-**ArgR**)_2_. We suggest that the formation of (L-**GluR**)_2_ proceeds through hydrophobic clustering. All experimental data (spectra, binding studies and calculations) in the paper are correct. The interpretation of data for (L-**ArgR**)_2_ also remains correct, without reservations. It is still claimed that both compounds form dimeric capsules, and are effective in encapsulation of guest molecules and the calculated values of K_ass_ are correct within an experimental error. Therefore, despite different structure of **(**L-**GluR**)_2_, all conclusions remain valid.

The following corrections have been made accordingly:

In **Introduction**, second paragraph, page 2, it was previously stated: “We demonstrate that electrostatic interactions between the backbones of amino acids are effective in inducing their self-assembly in water, and this mode leaves side chains available for additional interactions.”

The corrected sentence appears below:

“We demonstrate that electrostatic and hydrophobic interactions are effective in inducing their self-assembly in water.”

In section **3 Original Research**, “*3.1 Results and Discussion”, “3.1.1 Design and Synthesis”*, second paragraph, page 3, it was previously stated: “Among the amino acids tested (Glu, Asp, Arg, and His), only glutamic acid and arginine gave the target cavitands (L-**GluR**, D-**GluR**, and L-**ArgR**).”

The corrected section appears below:

“Among the amino acids tested (Glu, Asp, Arg, and His), only glutamic acid and arginine gave the cavitands (L-**GluR**, D-**GluR**, and L-**ArgR**). Nevertheless, the ESI MS spectrum of L-**GluR** shows the signal of (M-4H_2_O – H]^−^ (**Supplementary Figure S66**), and in the HMBC spectrum, we found an additional low-intensity correlation between H_g_ and C_k_ (**Supplementary Figure S4**), which suggests that L-**GluR**, in contrary to L-**ArgR**, underwent intramolecular cyclization (Figure 1C).”

In section “*3.1.2 Self-Assembly of Homocapsules*”, first paragraph, page 3, it was previously stated: “The cavitands L-**GluR** and L-**ArgR** are expected to self-assemble to dimeric capsules using electrostatic interactions between zwitterionic structures involving their “backbone groups” (Figure 1B).”

The corrected sentence appears below:

“The cavitands are expected to self-assemble to dimeric capsules using electrostatic interactions between zwitterionic structures involving their “backbone groups” (for L-**ArgR**, Figure 1B; **Supplementary Figure S69**) or by hydrophobic clustering (for L-**GluR**, **Supplementary Figure S68**).”

In section “*3.1.2. Self-Assembly of Homocapsules*”, second paragraph, page 4, it was previously stated: “This value is in reasonable agreement with the estimated *r*_H_ for a dimeric (L-**GluR**)_2_ [*r*_H_ (dimer) = 10.8 Å, *r*_H_ (cavitand) = 9.2 Å calculated by averaging the dimensions of the model structure, **Supplementary Figures S68 and S69**].”

The corrected sentence appears below:

“This value is in reasonable agreement with the estimated *r*_H_ for a dimeric (L-**GluR**)_2_ [*r*_H_ (dimer) = 9.1 Å, *r*_H_ (cavitand) = 7.2 Å calculated by averaging the dimensions of the model structure, **Supplementary Figures S68 and S69**].”

In section “*3.1.2 Self-Assembly of Homocapsules*”, paragraph 5, page 5, it was previously stated: “Quite intriguingly, L-**GluR** does not self-assemble in DMSO, which is a less polar solvent than water.”

The corrected sentence appears below:

“In agreement with the hydrophobic character interactions, L-**GluR** does not self-assemble in DMSO.”

In section “*3.1.5 Screening of Encapsulation Properties*”, first paragraph, page 6, it was previously stated: “Assuming that the binding motif of hydrophilic capsules is similar to that of hydrophobic (L-**PheR**)_2_, the size of the cavity can be estimated as c.a. 300 Å^3^, and it can be expected that the interior of the cavity has a mixed character—the “poles” are hydrophobic, while the “equator” part is polar (Kuberski and Szumna, 2009).”

The corrected sentence appears below:

“Assuming that the binding motif of hydrophilic capsules is similar to that of hydrophobic (L-**PheR**)_2_, in case of (L-**ArgR**)_2_ (Kuberski and Szumna, 2009) or based on hydrophobic clustering, in case of (L-**GluR**)2, the size of the cavity can be estimated as c.a. 300 Å^3^.”

The authors apologize for these errors and state that this does not change the scientific conclusions of the article in any way. The original article has been updated.

