# Supplementary material for: Chiral Water-Soluble Molecular Capsules With Amphiphilic Interiors
Source: Front Chem. 2022 Apr 14;10:883093. doi: 10.3389/fchem.2022.883093 (PMC9047736; doi:10.3389/fchem.2022.883093)
Supplement: Supplementary file 1 [file DataSheet1.pdf]

SUPPLEMENTARY INFORMATION

Chiral water-soluble molecular capsules with amphiphilic interiors

Arkadiusz Sakowicz, Agnieszka Szumna*

Table of content

1. Synthesis and compounds characterization 2

2. NMR spectra 4

   2.1. NMR spectra of capsules 4

   2.2. DOSY experiments 11

   2.3. Complexation of guests 28

3. UV and ECD spectra 41

4. IR spectra 45

5. MS spectra 47

6. Calculations of the size of the capsule 48

1. Synthesis and compounds characterization

All solvents and chemicals used were purchased from Sigma Aldrich, TCI Europe N. V., Roth, Chem Impex Inc., and Euriso-top, were of reagent grade and were used without further purification.

All reactions were carried out under the atmosphere of the air.

ECD spectra were recorded on ECD Jasco J-715 spectropolarimeter.

IR spectra were recorded on SHIMADZU IRTracer100 spectrophotometer.

High-resolution mass spectra were recorded on SYNAPT spectrometer.

Optical rotations were recorded on Jasco P-2000 polarymeter.

Synthesis of (L-GluR)$_2$

Resorcin[4]arene **R**[1] (2.16 g, 3 mmol), L-glutamic acid (2.21 g 15 mmol), and formaldehyde (40 % aqueous solution, 0.36 ml, 12 mmol) were added to the mixture of DMF and water (1:3, 80 ml). The solution was heated to 60°C and stirred at that temperature for 3 days. After cooling the reaction was evaporated and the precipitate was washed with water, acetonitrile, again with water, and dried, yield: 50%. ^{1}H NMR (600 MHz, D_2O) δ 7.03 (s, 1H); 4.36 (d, *J* = 14.7 Hz, 1H); 4.27 (t, *J* = 7.8 Hz, 1H), 4.17 (d, *J* = 14.6 Hz, 1H); 4.01 (m, 1H); 3.48 (t, *J* = 6.6 Hz, 2H); 2.36 (m, 2H); 2.19 (m, 1H); 2.16 (m, 1H); 2.01 (m, 2H); 1.80 (m, 1H); 1.36 (t, *J* = 7.5 Hz, 2H); ^{13}C NMR (150 MHz, D_2O) δ 179.5; 178.9; 150.2; 150.1; 125.4; 124.2; 112.1; 63.0; 61.6; 61.5; 36.2; 34.3; 29.9; 29.8; 29.3; 23.1. IR (KBr): *ν*/cm^{-1} 3315; 2942; 1874; 1730; 1644; 1230; 658. Optical rotation: $[\alpha]_D$ = 18.0 (c = 9.99 mg / ml, H_2O, pH = 4.8, 22.7 °C). HRMS (ESI-TOF): calcd m/z for $C_{64}H_{75}N_4O_{24}$ $[M-H]^-$ 1283.4771 found 1283.4757.

Synthesis of (L-ArgR)$_2$

L-Arginine monohydrochloride (0.44 g 2.01 mmol) was dissolved in water (pH 3.5, 5 ml). Further, methanol (5 ml), resorcin[4]arene **R** (0.30 g, 0.41 mmol) and formaldehyde (40 % aqueous solution, 0.12 ml, 1.64 mmol) were added. The solution was heated to 60°C and stirred at that temperature for 1 day. After cooling the reaction was evaporated to dryness and the product was purified on the Sephadex LH-20 column, yield: 47%. ^{1}H NMR (600 MHz, D_2O) δ 7.26 (s, 1H); 4.27 (t, *J* = 7.8 Hz, 1H); 4.15 (d, *J* = 13.2 Hz, 1H), 4.04 (d, *J* = 13.1 Hz, 1H); 3.48 (t, *J* = 6.3 Hz, 2H); 3.41 (t, *J* = 6.0 Hz, 1H); 2.86 (t, *J* = 7.2 Hz, 2H); 2.14 (m, 2H); 1.68 (m, 2H), 1.34 (bm, 2H), 1.30 (m, 2H); ^{13}C NMR (150 MHz, D_2O) δ 173.3; 156.6; 151.0; 151.0; 126.5; 108.3; 61.5; 60.8; 41.1; 40.3; 34.2; 29.8; 29.3; 26.6; 23.8. IR (KBr): *ν*/cm^{-1} 3346; 3177; 2938; 2870; 1634; 1472; 1400; 1053. Optical rotation: $[\alpha]_D$ = 11.5 (c = 10.00 mg / ml, H_2O, pH = 4.8, 22.6 °C). HRMS (ESI-TOF): calcd m/z for $C_{68}H_{105}N_{16}O_{20}$ $[M-H]^+$ 1465.7691 found 1465.7704.

Synthesis of (D-GluR+L-ArgR)

To a water solution of (D-**GluR**)$_2$ (5 mg, 2 µmol, in 2 ml of water at pH 5.0) a water solution of (L-**ArgR**)$_2$ (5 mg, 1.5 µmol, in 2 ml of water at pH 5.0) was added. The precipitate was formed, which was washed with water (2 x 5 ml) and dried. The products was obtained in 82 %. IR (KBr): *ν*/cm^{-1} 3377; 2940; 2870; 1643; 1464; 1408; 1057; 835. Optical rotation: $[\alpha]_D$ = 111.7 (c = 10.00 mg / ml, H_2O, pH = 9.0, 22.6 °C).

Synthesis of (L-GluR+L-ArgR)

To a water solution of (D-**GluR**)$_2$ (5 mg, 2 μmol, in 2 ml of water at pH 5.0) a water solution of (L-**ArgR**)$_2$ (5 mg, 1.5 μmol, in 2 ml of water at pH 5.0) was added. The precipitate was formed, which was washed with water (2 x 5 ml) and dried. The product was obtained in 80 %. IR (KBr): ν/cm^{-1} 3381; 2938; 2868; 1645; 1458; 1055; 835. Optical rotation: $[\alpha]_D$ = 69.0 (c = 10.08 mg / ml, H_2O, pH = 9.0, 22.7 °C).

2. NMR spectra

2.1. NMR spectra of capsules

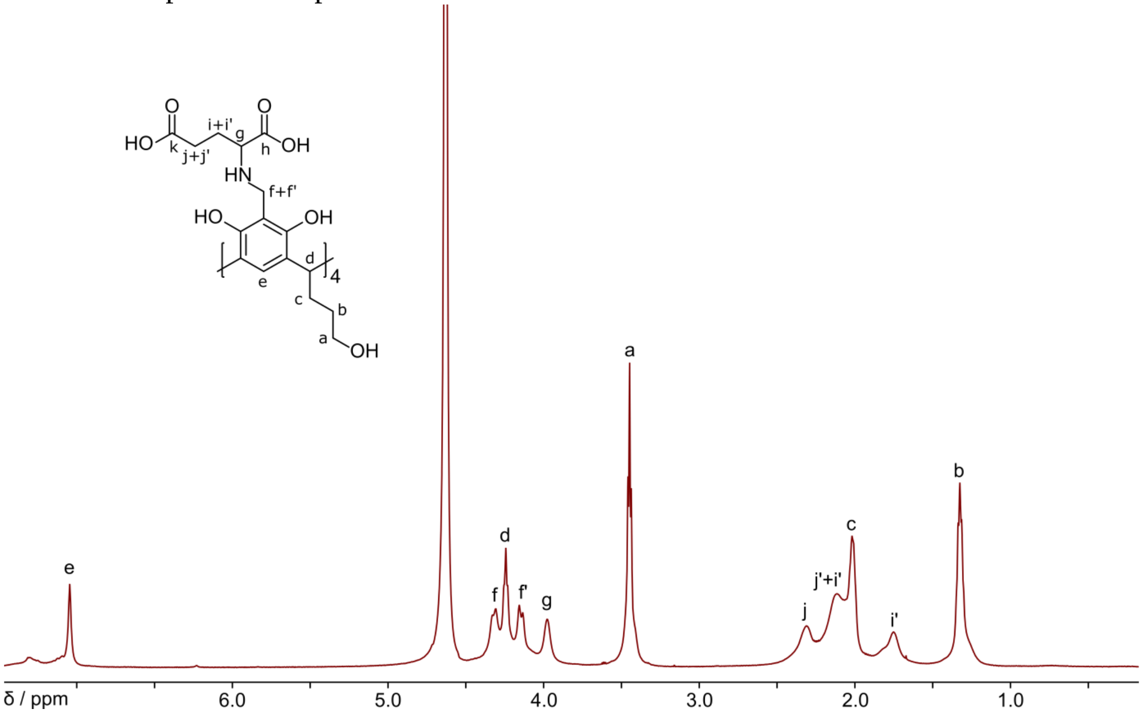

Figure S1. ^{1}H NMR spectrum of (L-**GluR**)$_2$ (600 MHz, 298 K, pD 4.8, D_2O, NaOD / DCl).

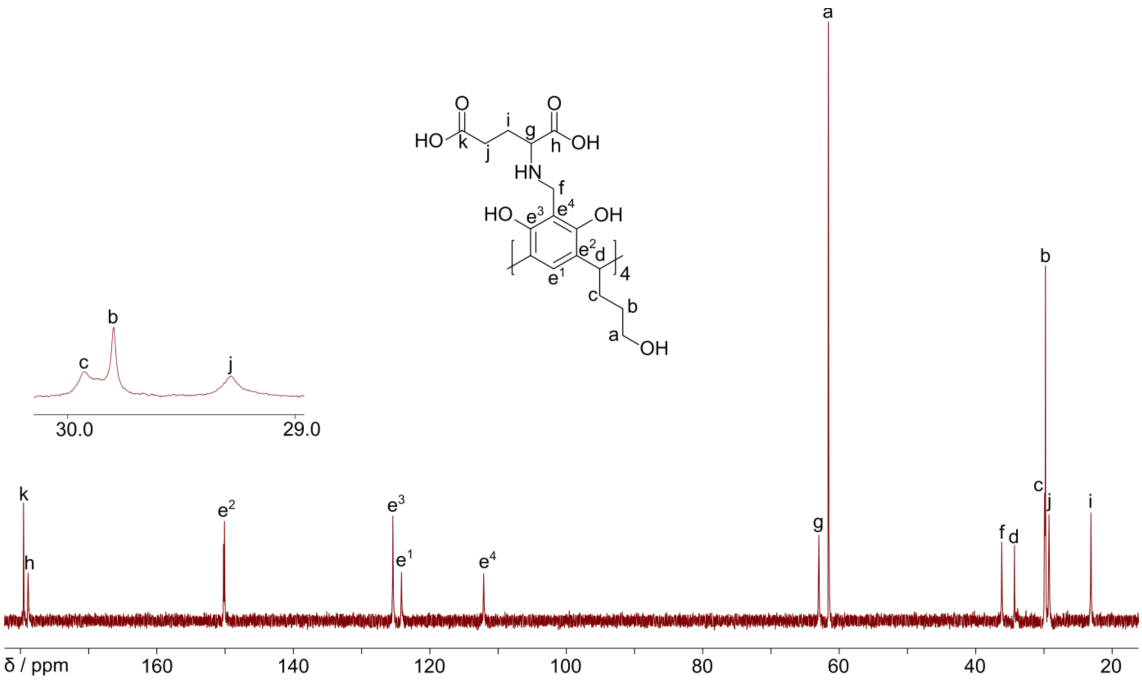

Figure S2. ^{13}C NMR spectrum of (L-**GluR**)$_2$ (150 MHz, 298 K, pD 4.8, D_2O, NaOD / DCl).

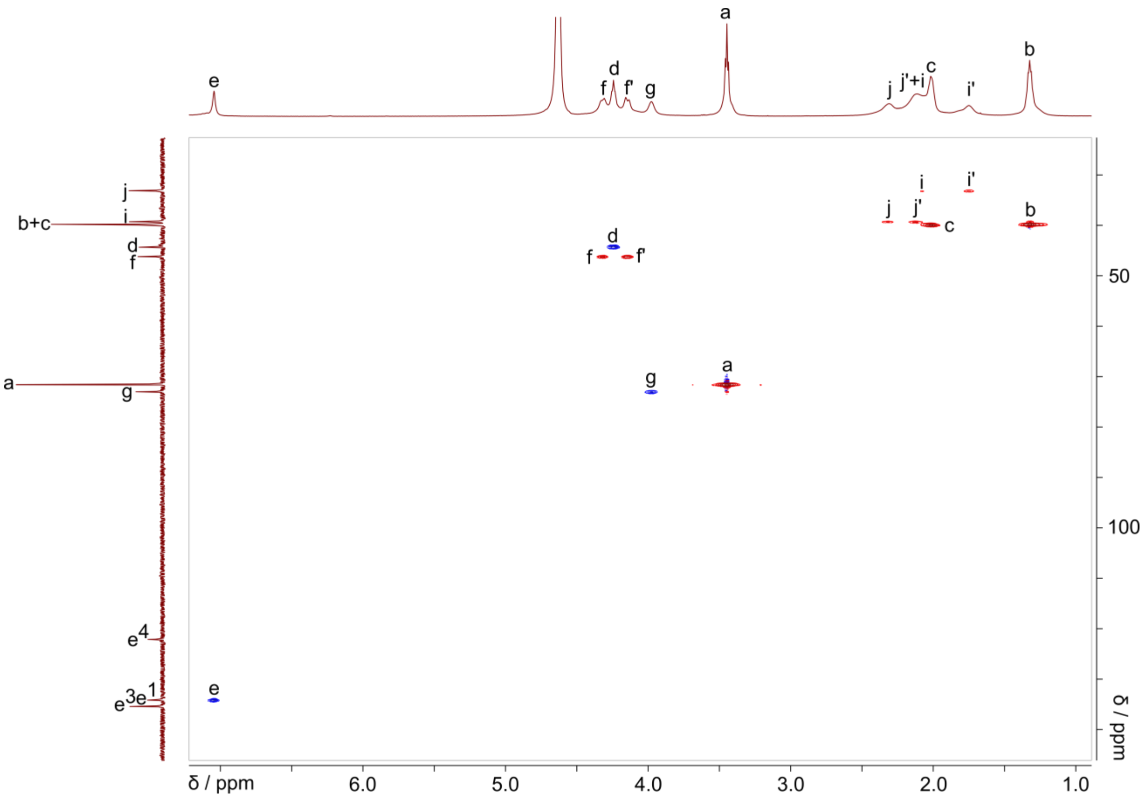

Figure S3. HSQC spectrum of (L-**GluR**)$_2$ (600 MHz, 298 K, pD 4.8, D_2O, NaOD / DCl).

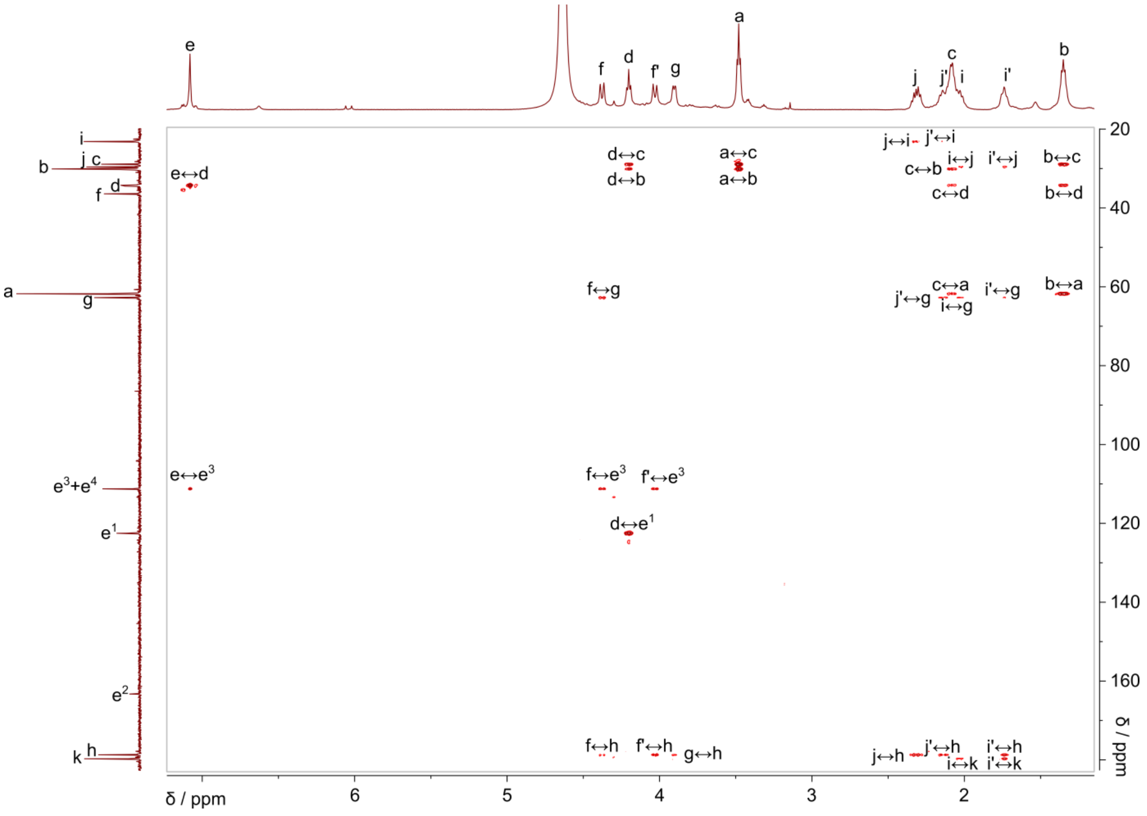

Figure S4. HMBC spectrum of (L-**GluR**)$_2$ (600 MHz, 298 K, pD 11.0, D_2O, NaOD / DCl).

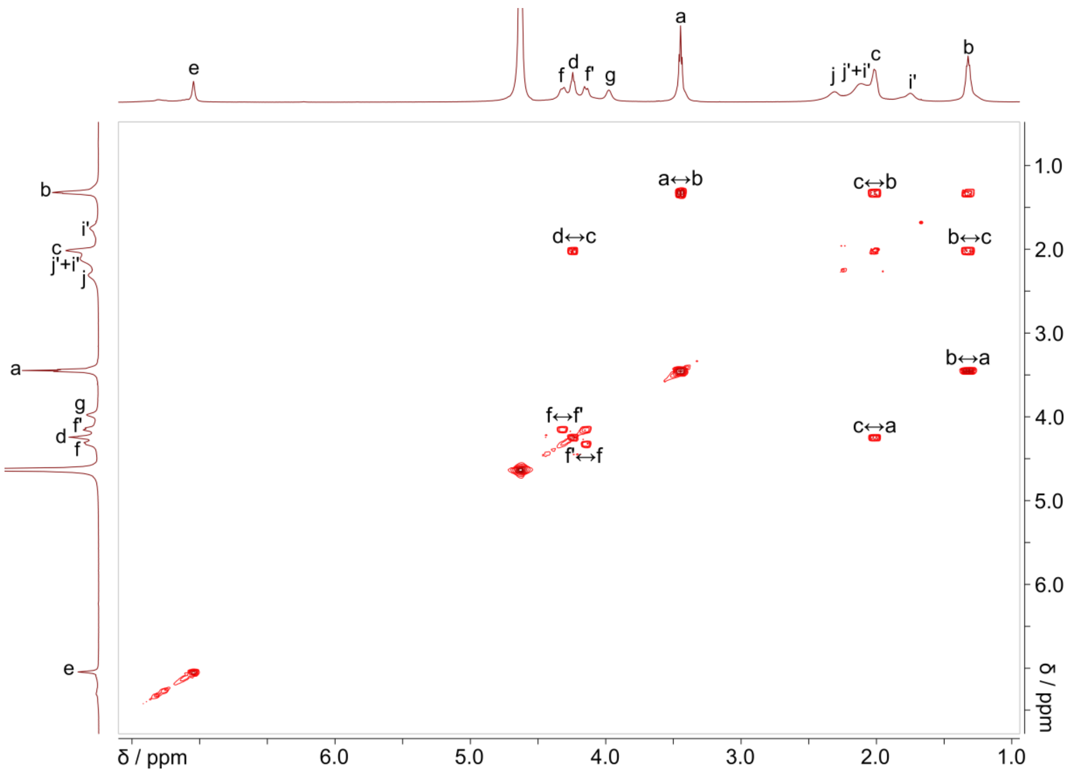

Figure S5. COSY spectrum of (L-**GluR**)$_2$ (600 MHz, 298 K, pD 4.8, D_2O, NaOD / DCl).

.

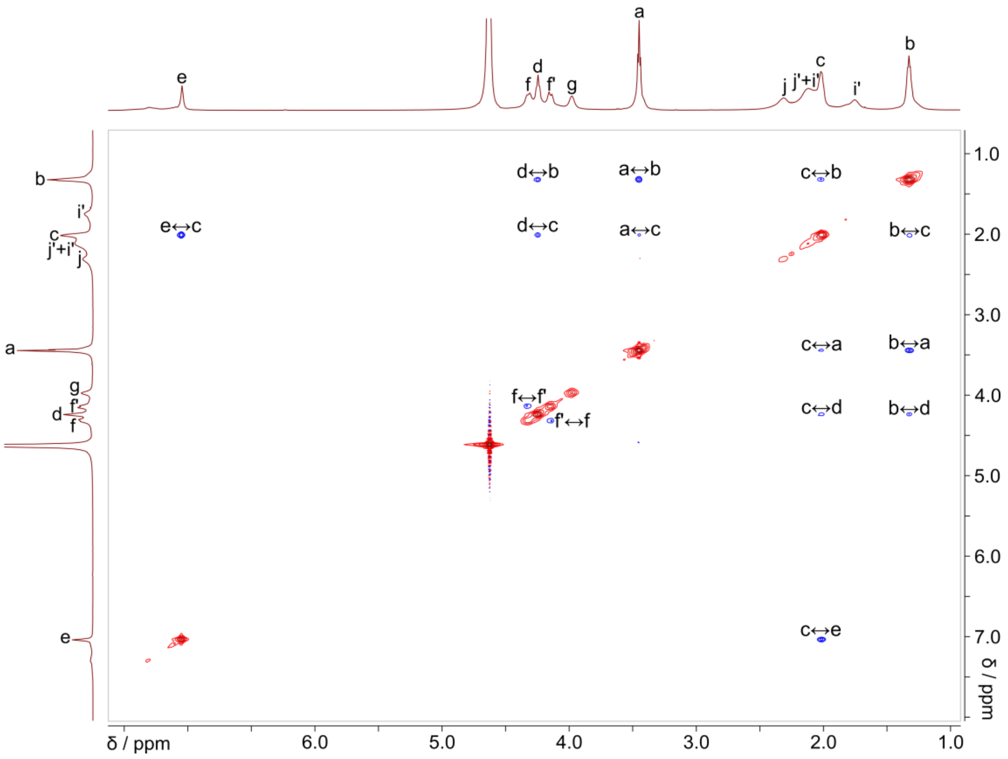

Figure S6. ROESY spectrum of (L-**GluR**)$_2$ (600 MHz, 298 K, pD 4.8, D_2O, NaOD / DCl).

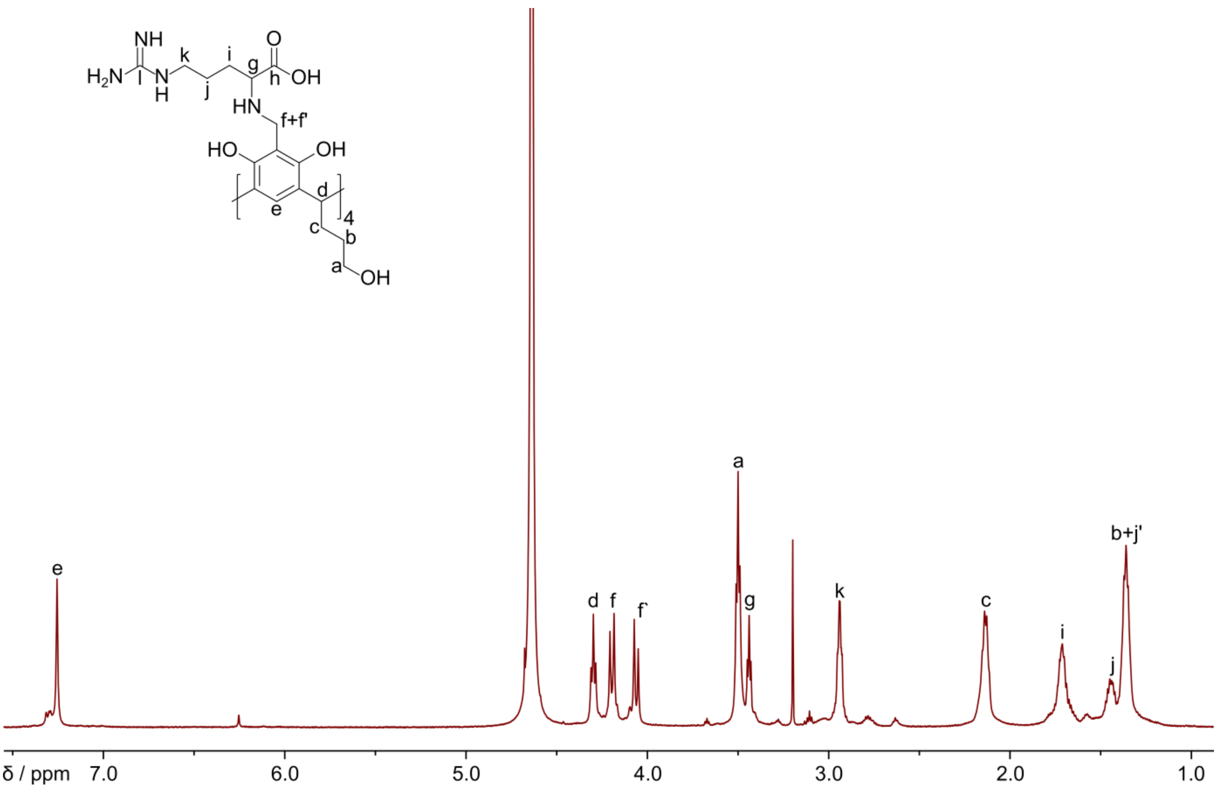

Figure S7. ^{1}H NMR spectrum of (L-**ArgR**)$_2$ (600 MHz, 298 K, pD 4.8, D_2O, NaOD / DCl).

.

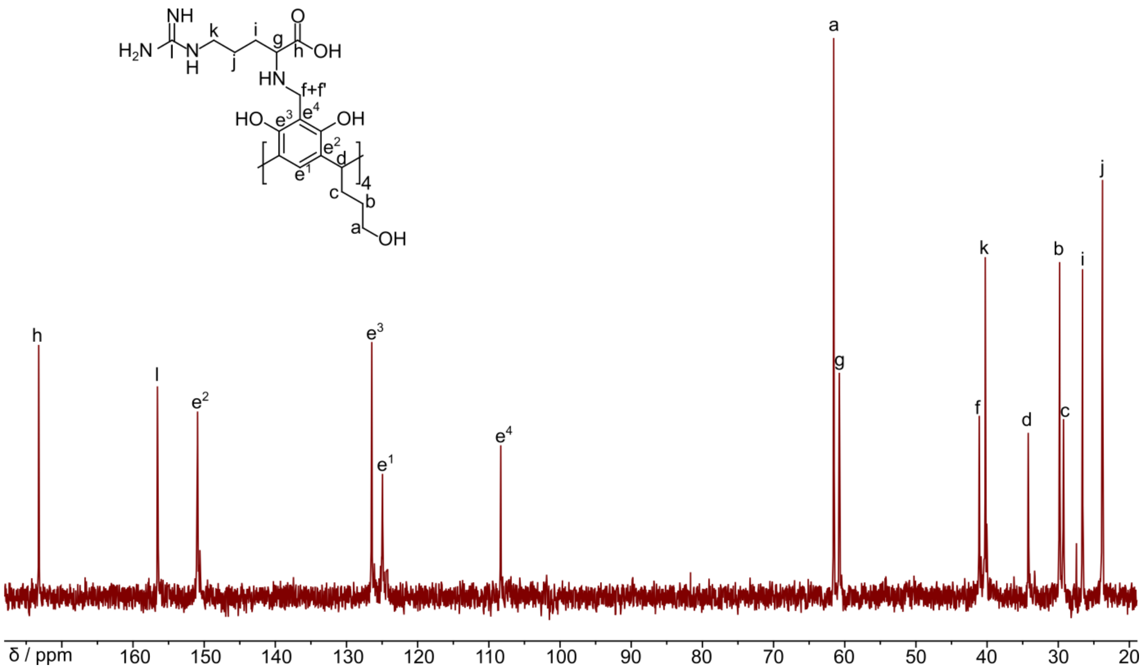

Figure S8. ^{13}C NMR spectrum of (L-**ArgR**)$_2$ (150 MHz, 298 K, pD 4.8, D_2O, NaOD / DCl).

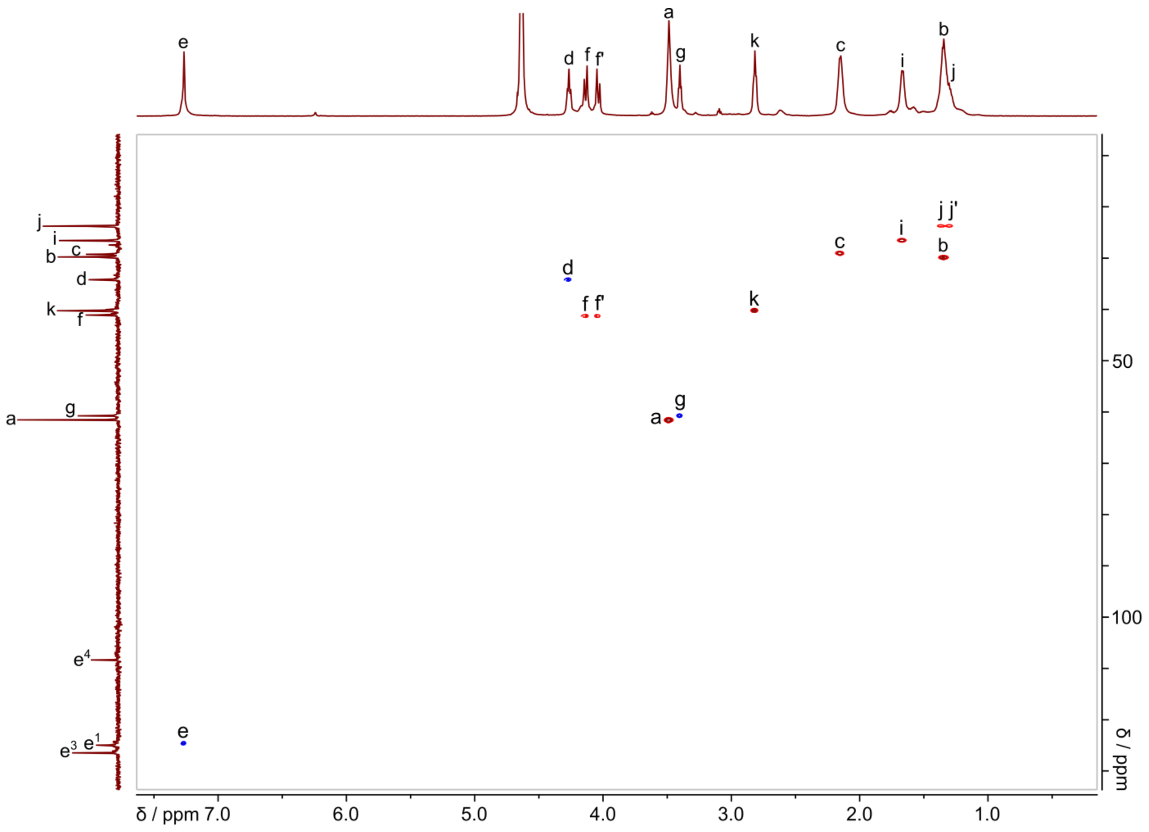

Figure S9. HSQC spectrum of (L-**ArgR**)$_2$ (600 MHz, 298 K, pD 4.8, D_2O, NaOD / DCl).

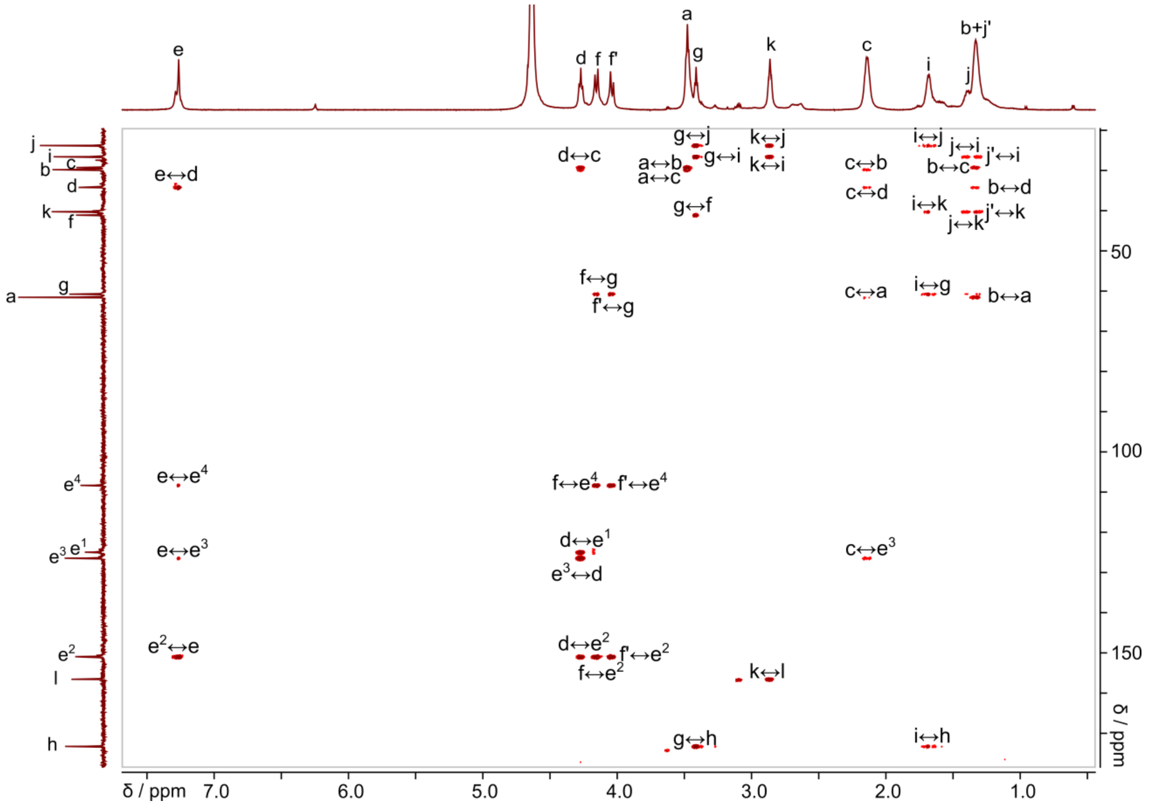

Figure S10. HMBC spectrum of (L-**ArgR**)$_2$ (600 MHz, 298 K, pD 4.8, D_2O, NaOD / DCl).

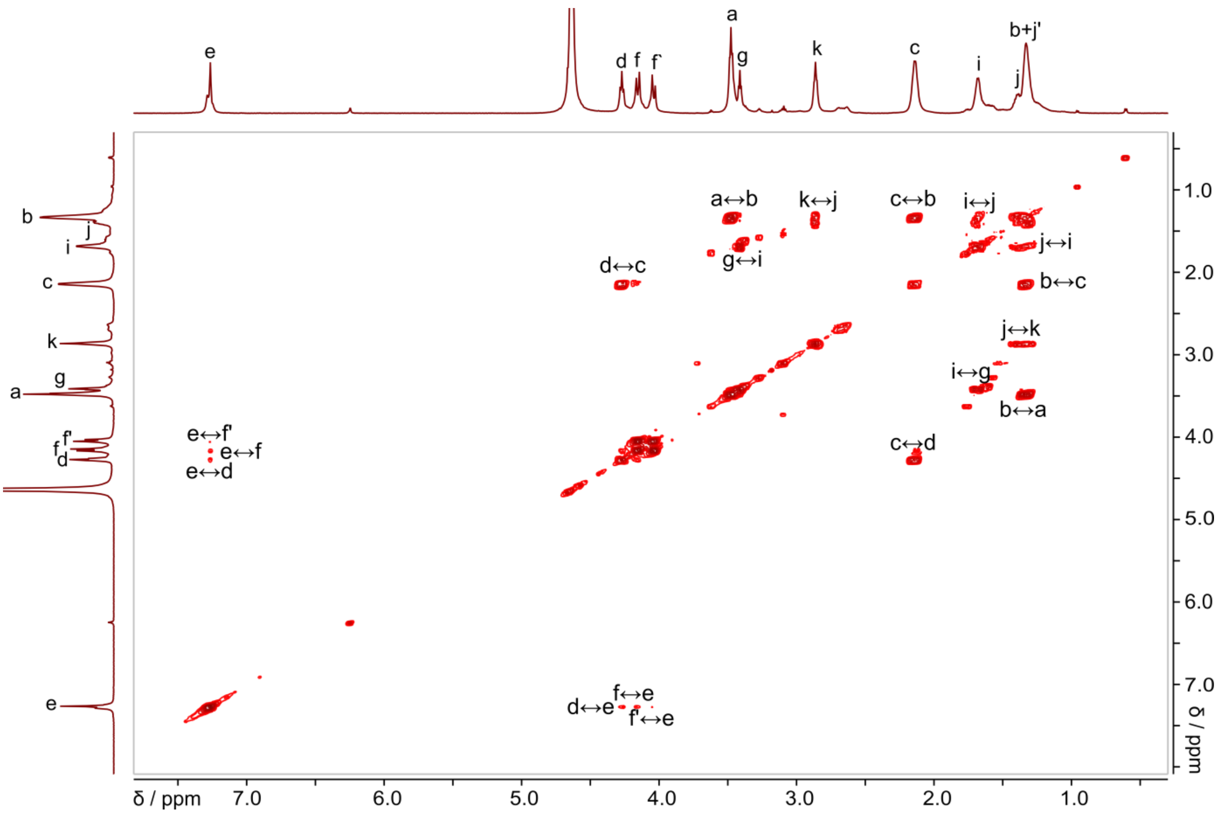

Figure S11. COSY spectrum of (L-**ArgR**)$_2$ (600 MHz, 298 K, pD 4.8, D_2O, NaOD / DCl).

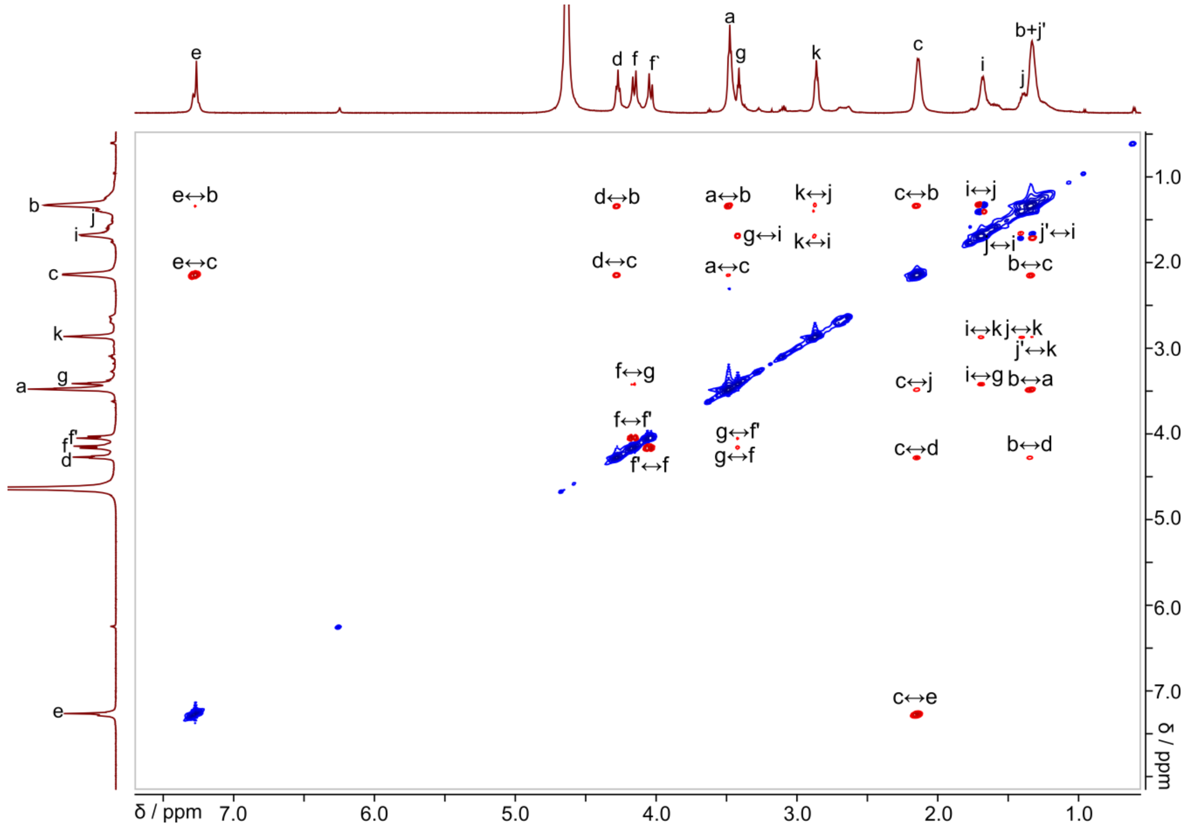

Figure S12. ROESY spectrum of (L-**ArgR**)$_2$ (600 MHz, 298 K, pD 4.8, D_2O, NaOD / DCl).

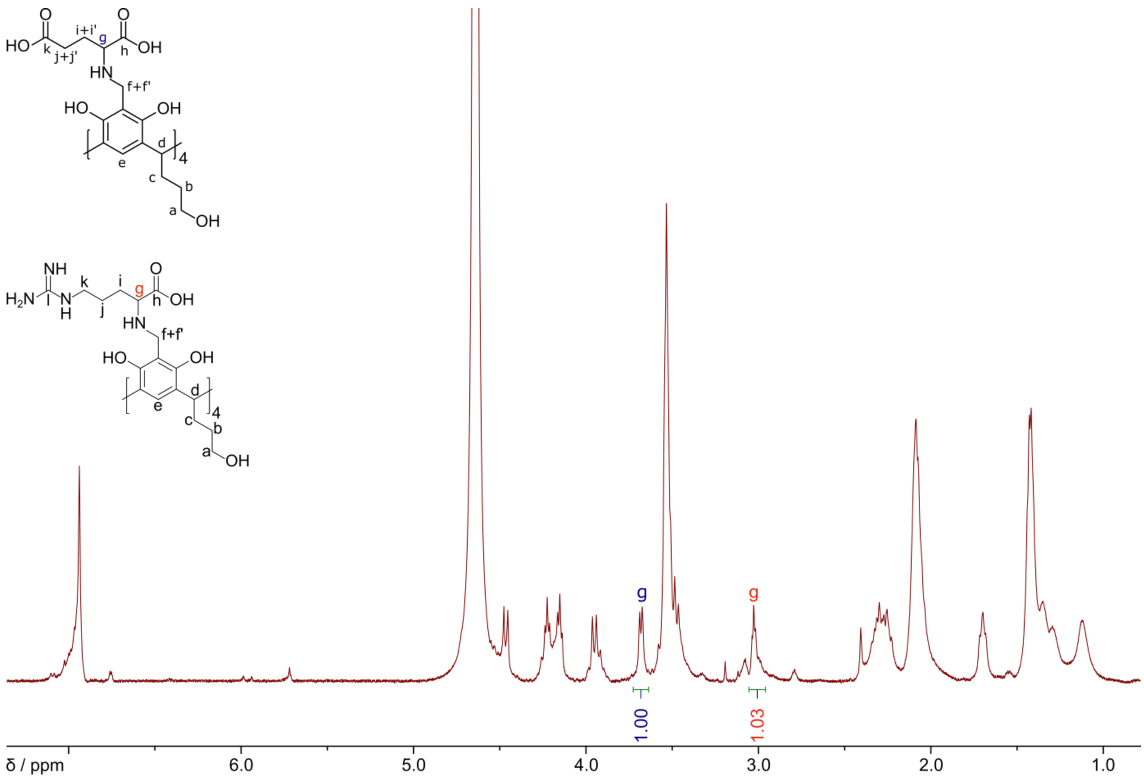

Figure S13. ^{1}H NMR spectrum of D-**GluR**-L-**ArgR** (600 MHz, 298 K, pD 13.0, D_2O, NaOD / DCl).

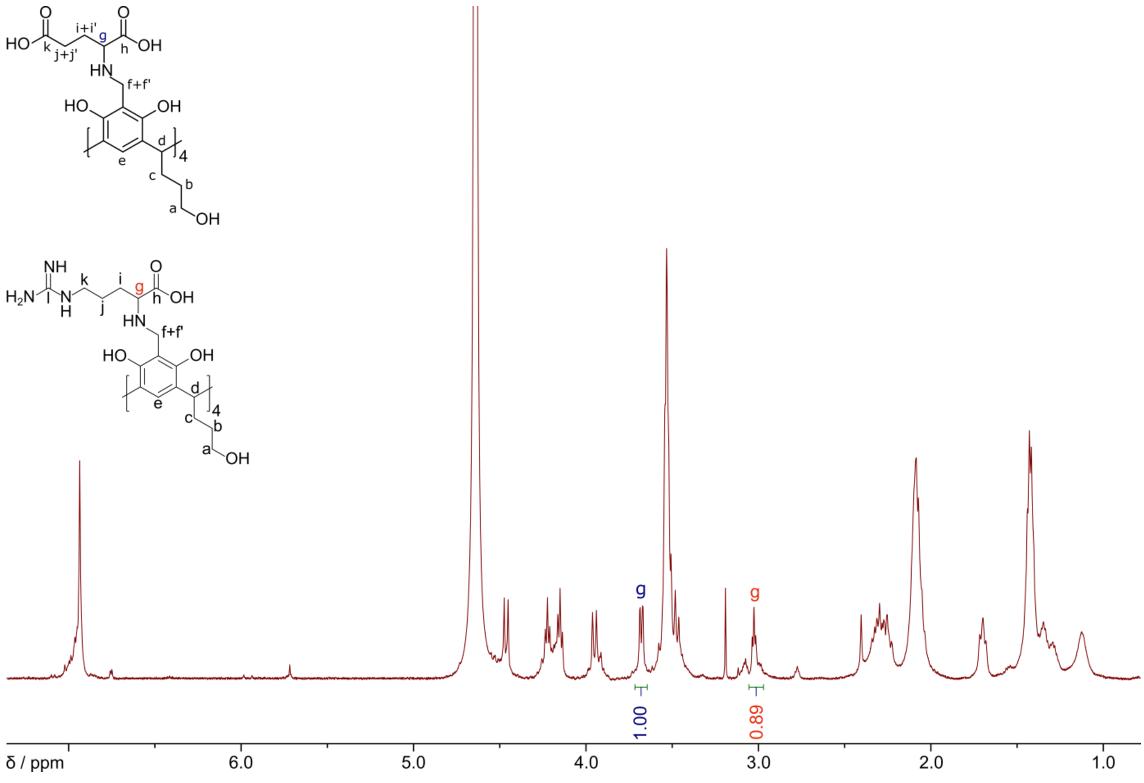

Figure S14. ^{1}H NMR spectrum of L-**GluR**-L-**ArgR** (600 MHz, 298 K, pD 13.0, D_2O, NaOD / DCl).

2.2. DOSY experiments

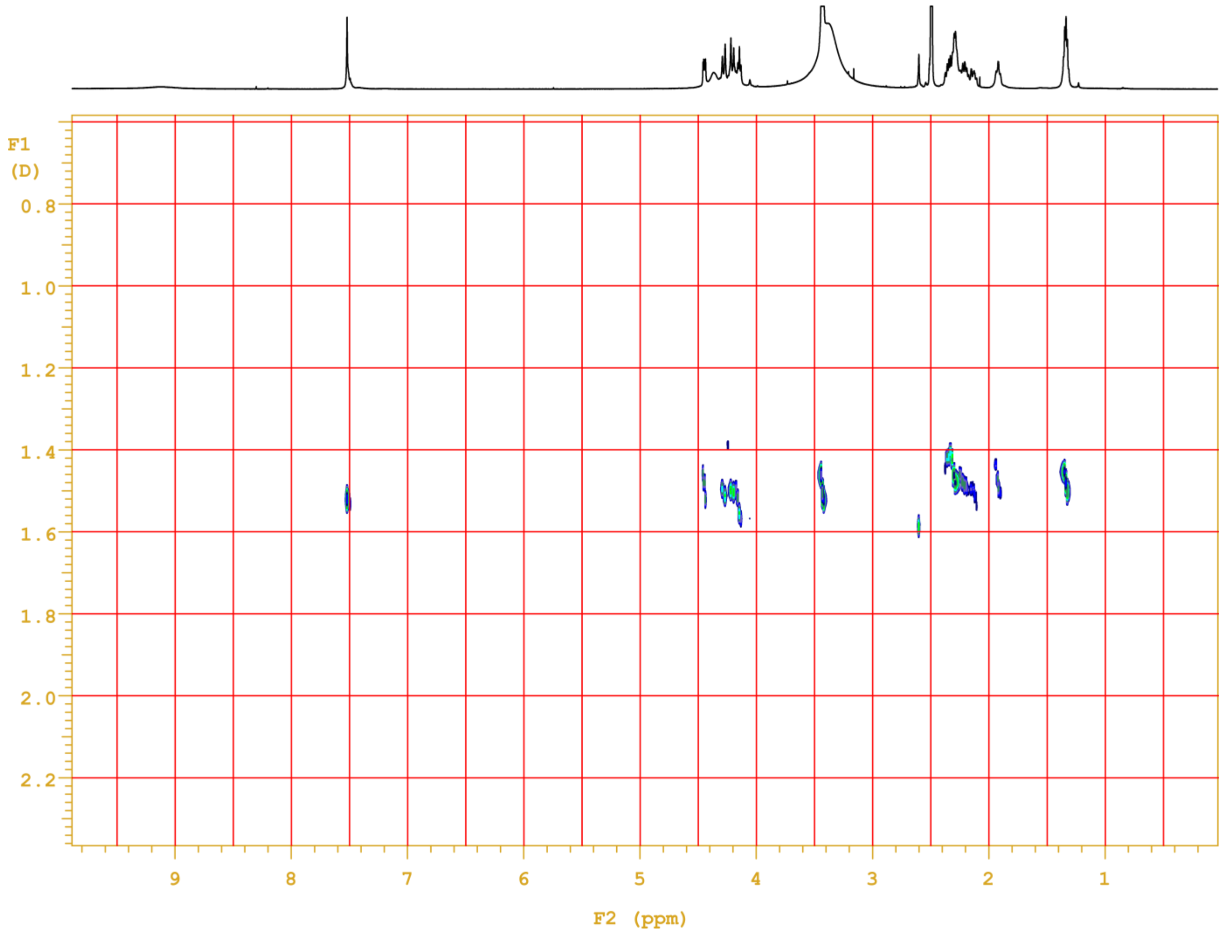

Figure S15. ^{1}H NMR and DOSY spectra of L-**GluR** (C = 20 mM) (600 MHz, 298 K, DMSO).

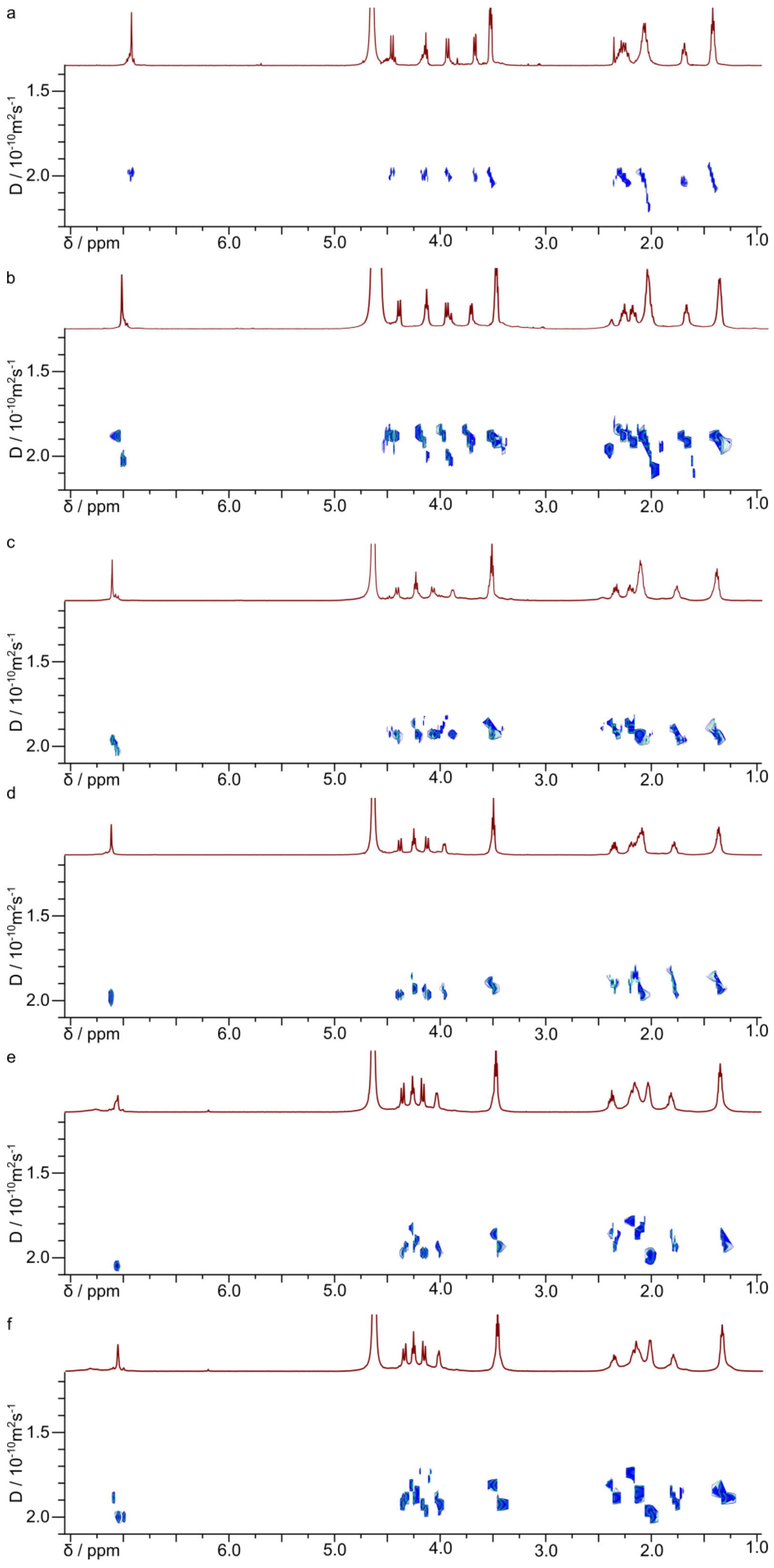

Figure S16. ^{1}H NMR and DOSY spectra of (L-**GluR**)$_2$ (C = 10 mM) at different pD a) 13.3 b) 12.0 c) 10.3 d) 9.0 e) 7.2 f) 5.5 (600 MHz, 298 K, D_2O, NaOD / DCl).

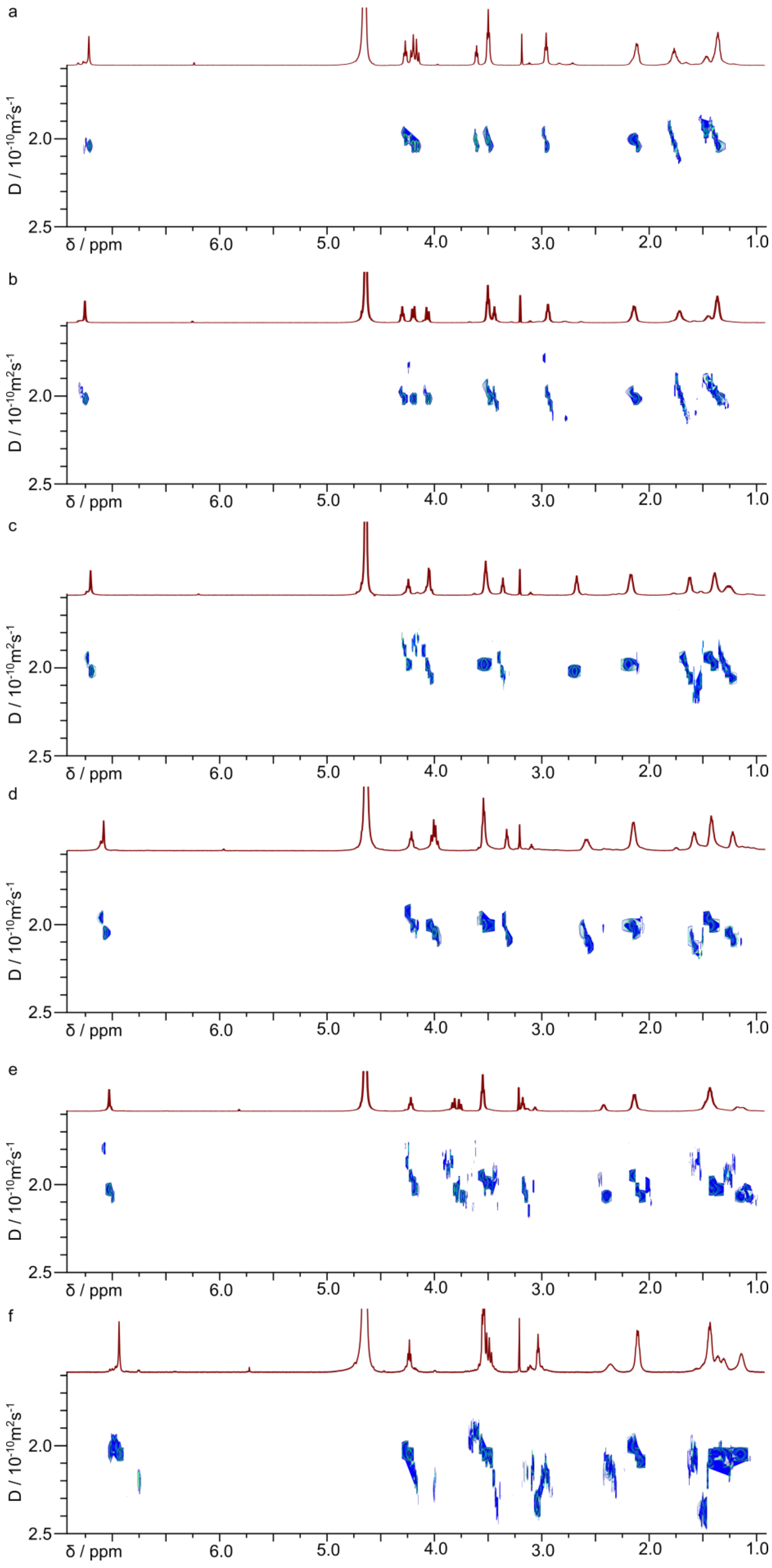

Figure S17. ^{1}H NMR and DOSY spectra of (L-**ArgR**)$_2$ (C = 10 mM) at different pD a) 1.9 b) 5.0 c) 8.0 d) 10.3 e) 12.0 f) 13.3 (600 MHz, 298 K, D_2O, NaOD / DCl).

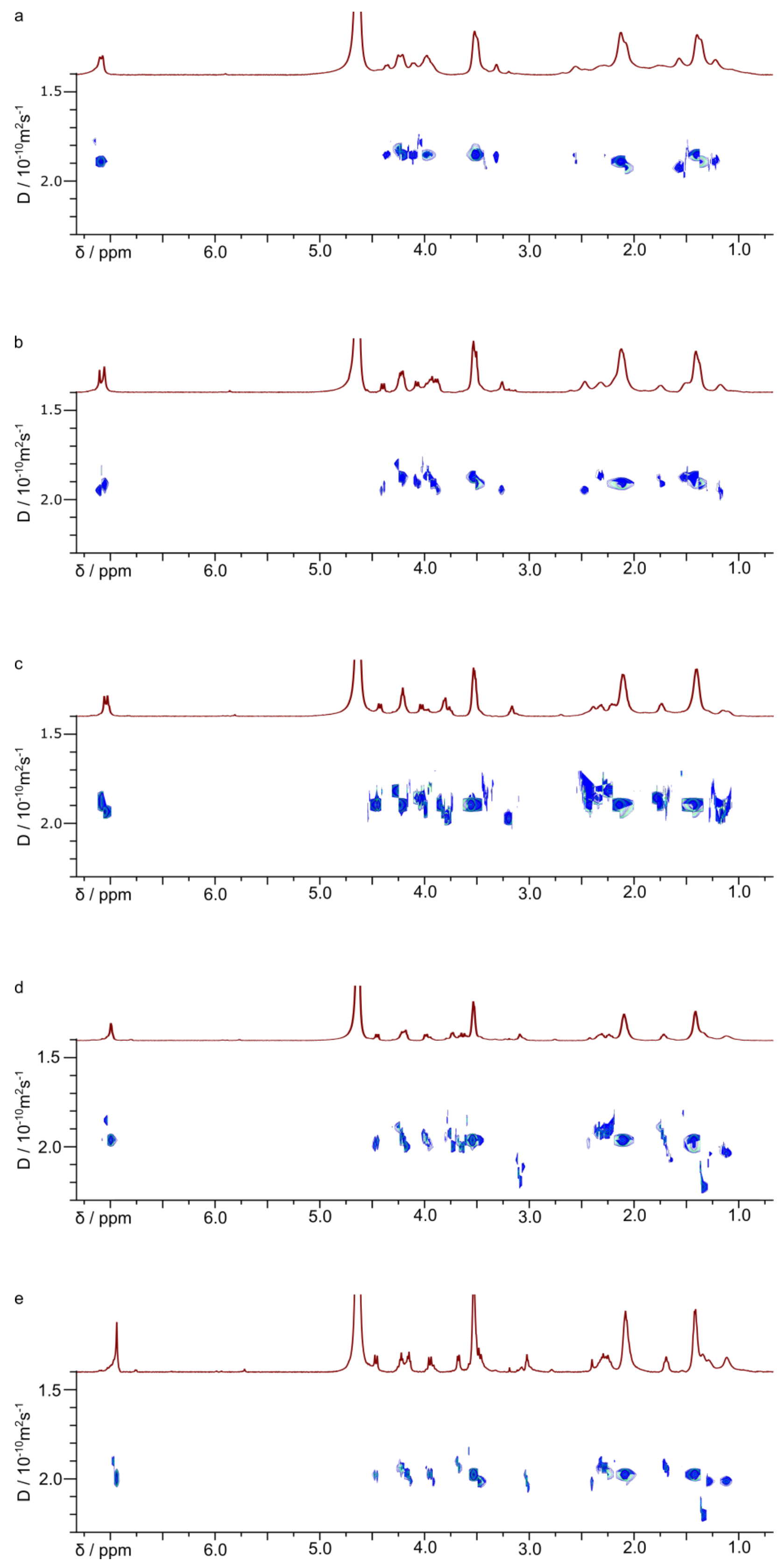

Figure S18. ^{1}H NMR and DOSY spectra of L-**ArgR**-D-**GluR** (C = 10 mM) at different pD a) 9.0 b) 10.0 c) 11.0 d) 12.0 e) 13.0 (600 MHz, 298 K, D_2O, NaOD / DCl).

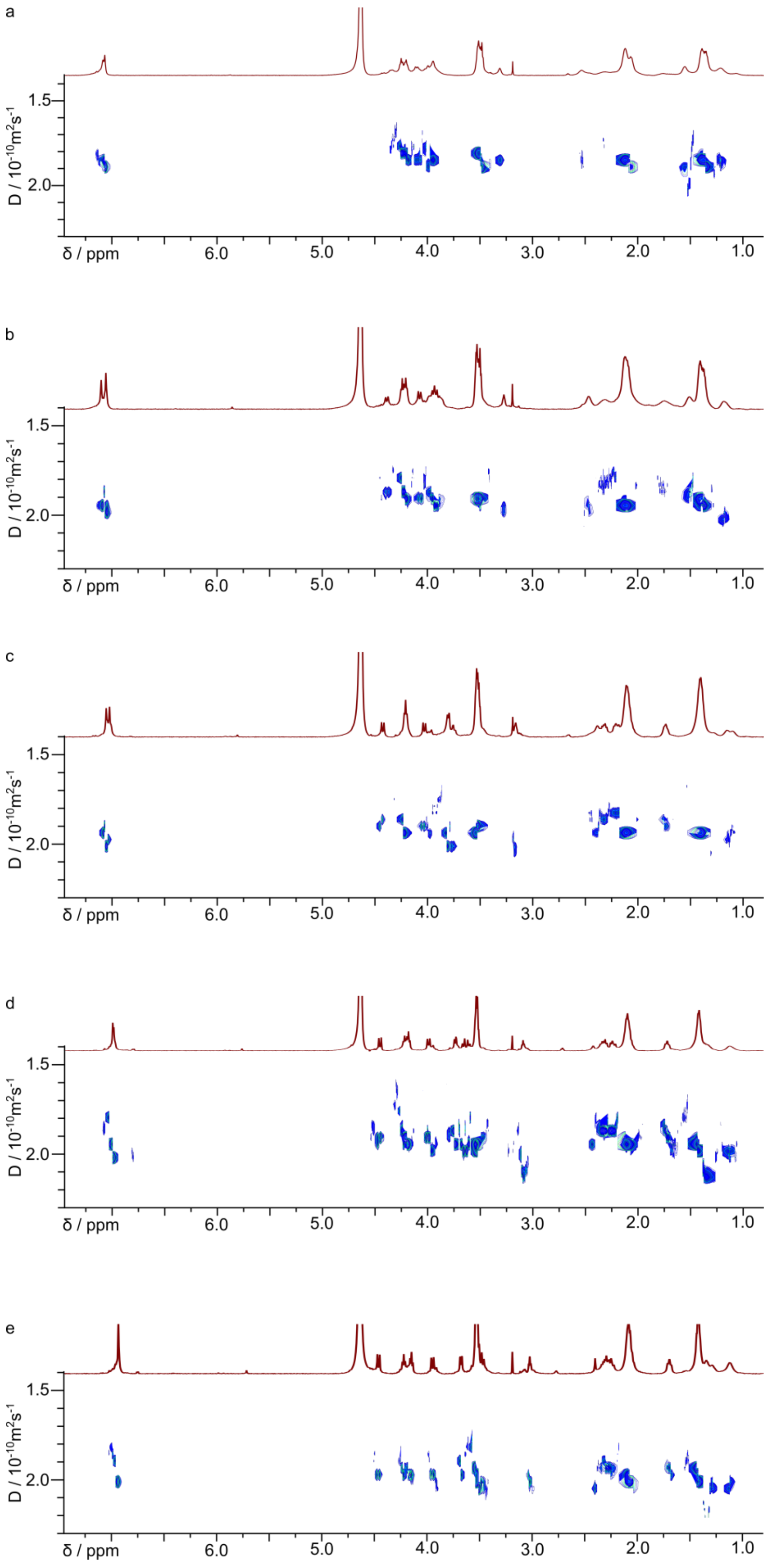

Figure S19. ^{1}H NMR and DOSY spectra of L-**ArgR**-L-**GluR** (C = 10 mM) at different pD a) 9.0 b) 10.0 c) 11.0 d) 12.0 e) 13.0 (600 MHz, 298 K, D_2O, NaOD / DCl).

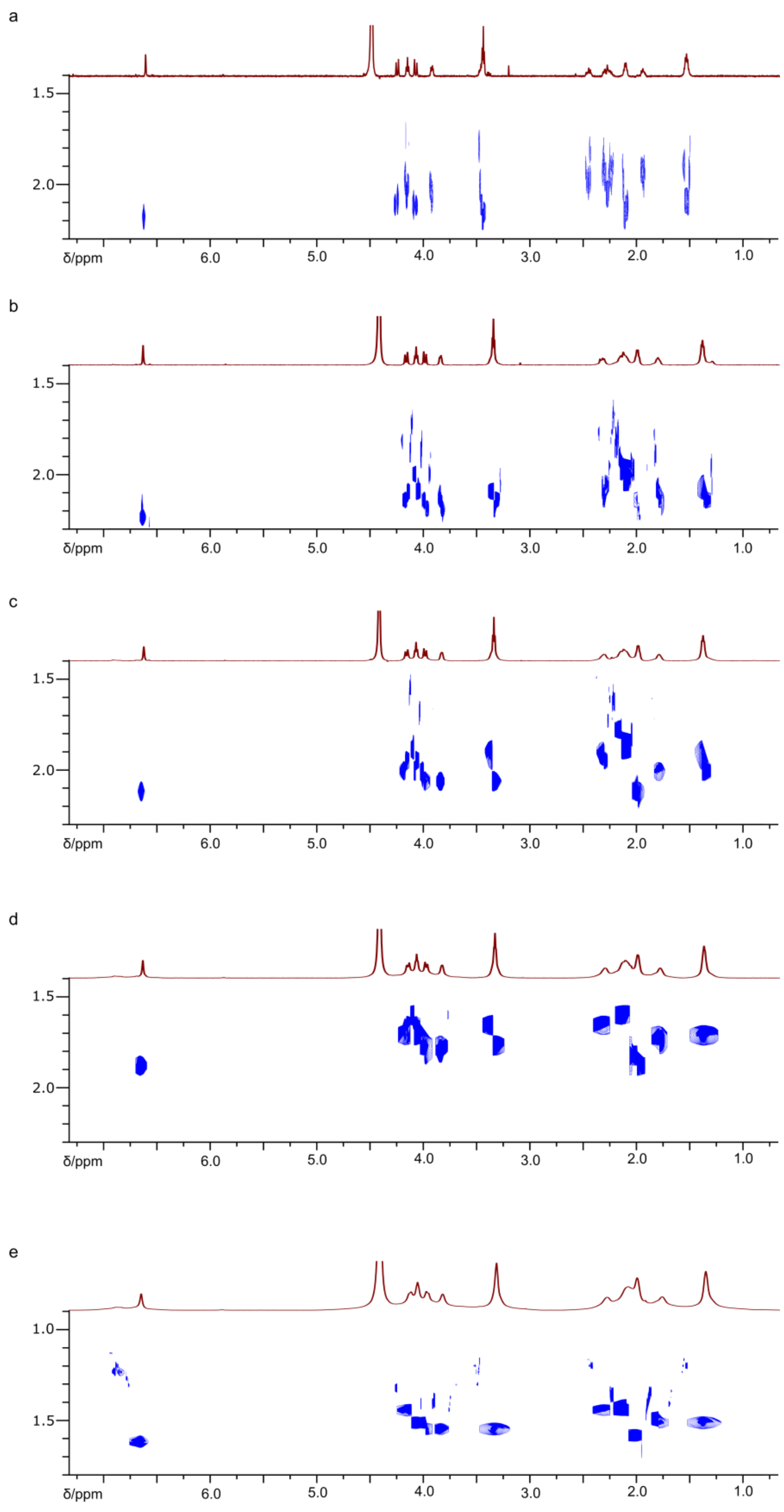

Figure S20. ^{1}H NMR and DOSY spectra of (L-**GluR**)$_2$ at pD 5.0 at different concentration a) 0.37 mM b) 1.8 mM c) 3.7 mM d) 10 mM e) 14.8 mM (600 MHz, 298 K, D_2O, NaOD / DCl).

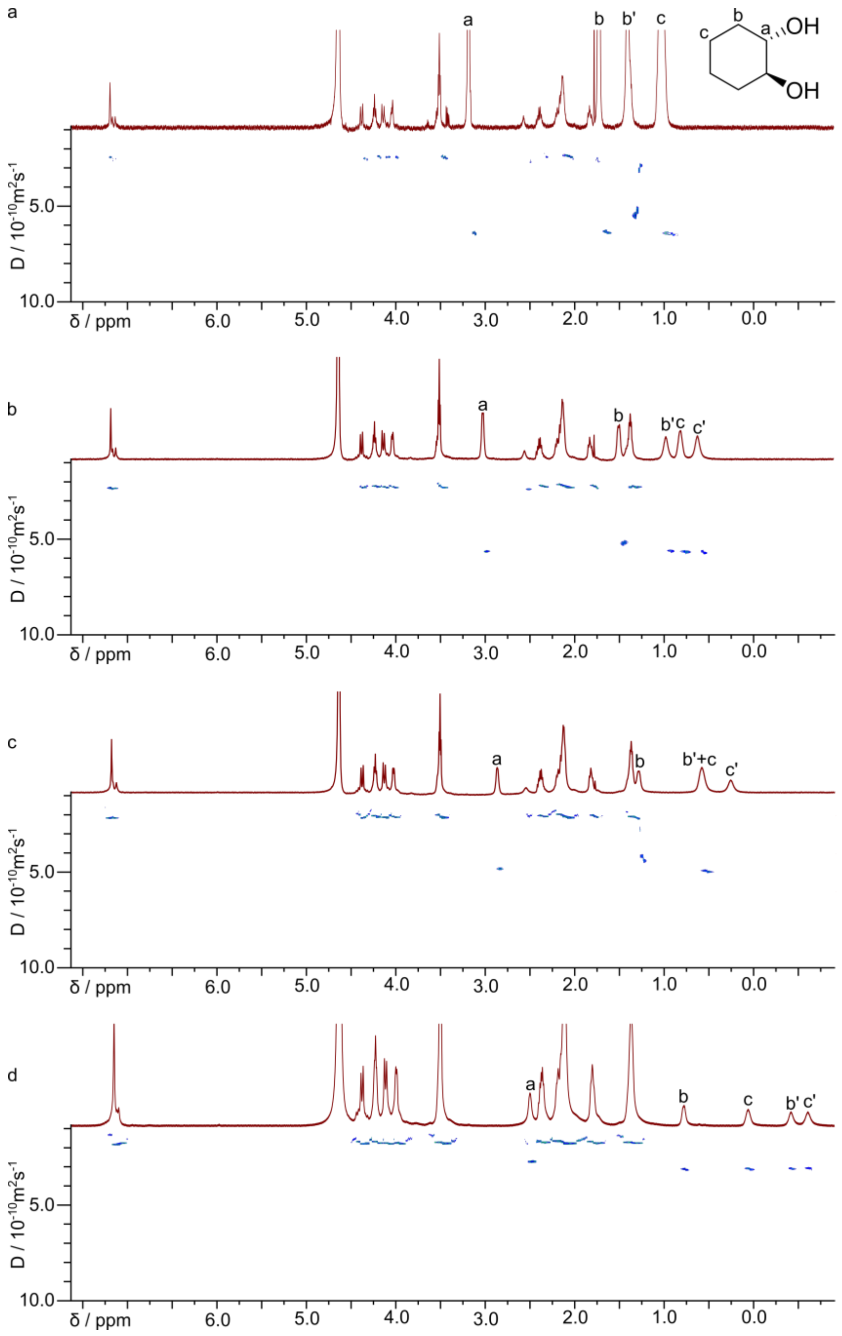

Figure S21. ^{1}H NMR and DOSY spectra for complexation of (1S,2S)-*trans*-1,2-cyclohexanediol **1,** (C = 10 mM) in (L-**GluR**)$_2$ at pD 9.0 at different concentration a) 0.37 mM b) 1.9 mM c) 3.7 mM d) 10 mM (600 MHz, 298 K, D_2O, NaOD / DCl).

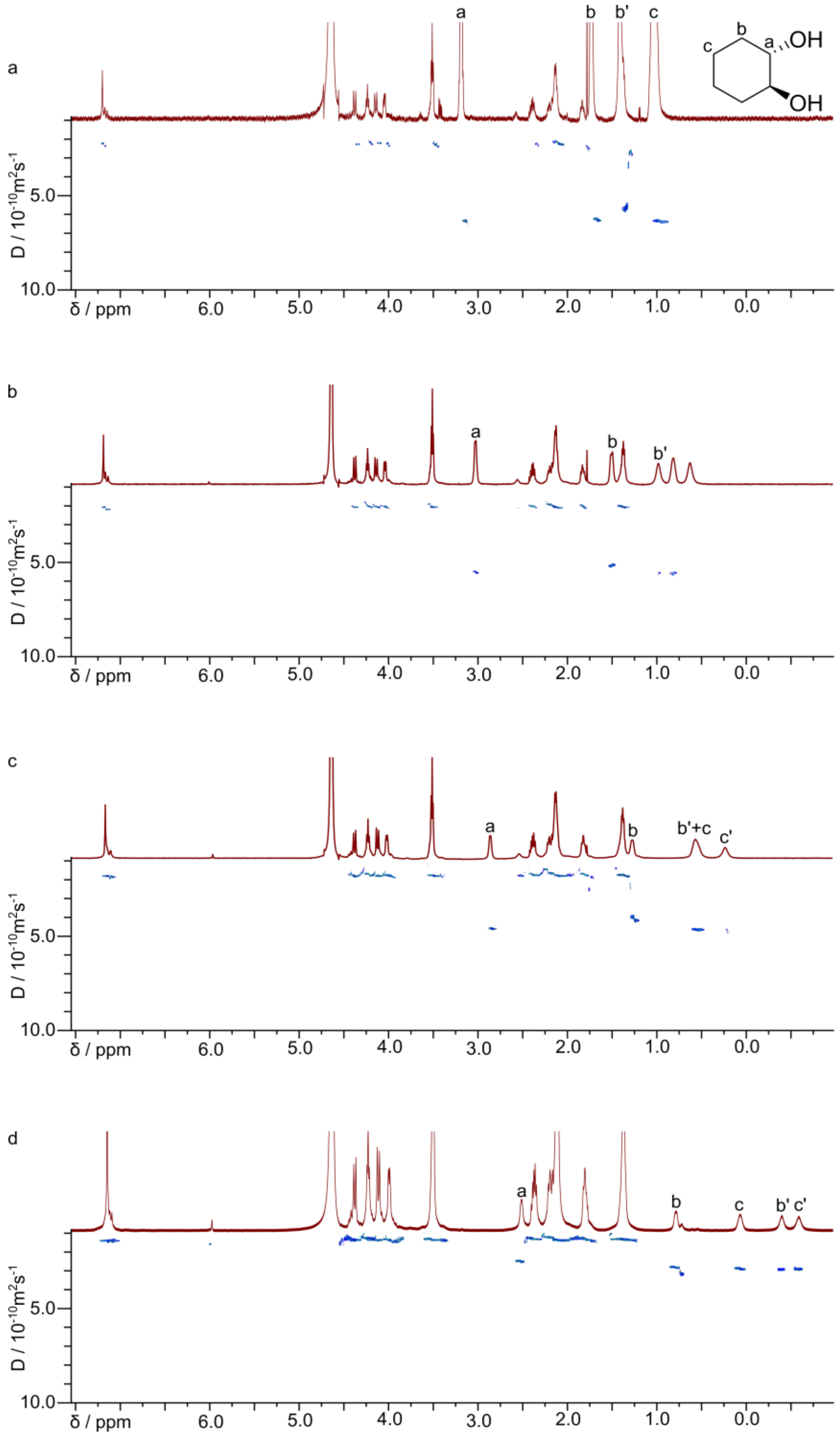

Figure S22. ^{1}H NMR and DOSY spectra for complexation of (1S,2S)-*trans*-1,2-cyclohexanediol **1**, (C = 10 mM) in (D-**GluR**)$_2$ at pD 9.0 at different concentration in a) 0.37 mM b) 1.9 mM c) 3.7 mM d) 10 mM (600 MHz, 298 K, D_2O, NaOD / DCl).

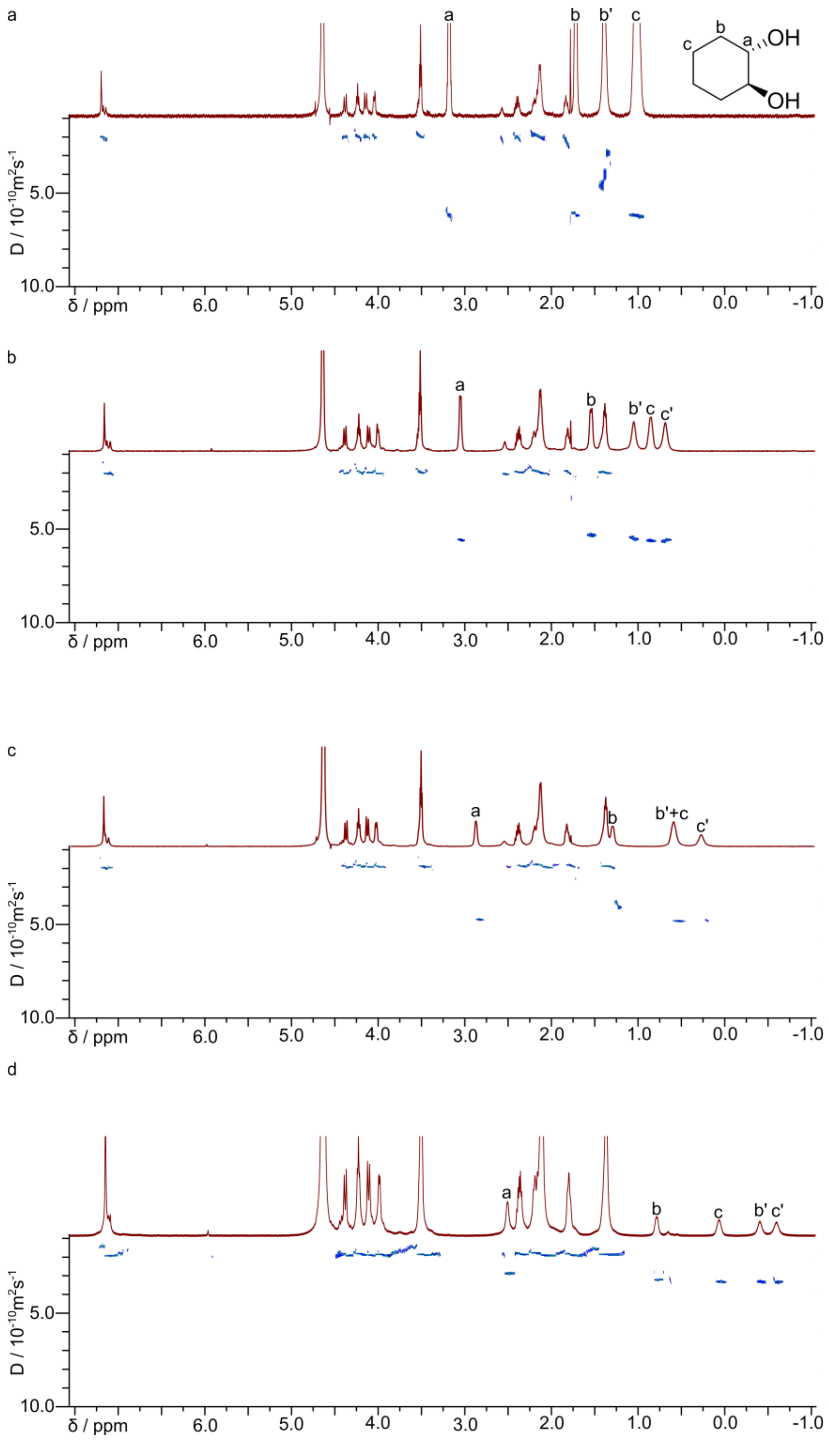

Figure S23. ^{1}H NMR and DOSY spectra for complexation of (1S,2S)-*trans*-1,2-cyclohexanediol **1**, (C = 10 mM) in D-**GluR**-L-**GluR** at pD 9.0 at different concentration a) 0.37 mM b) 1.9 mM c) 3.7 mM d) 10 mM (600 MHz, 298 K, D_2O, NaOD / DCl).

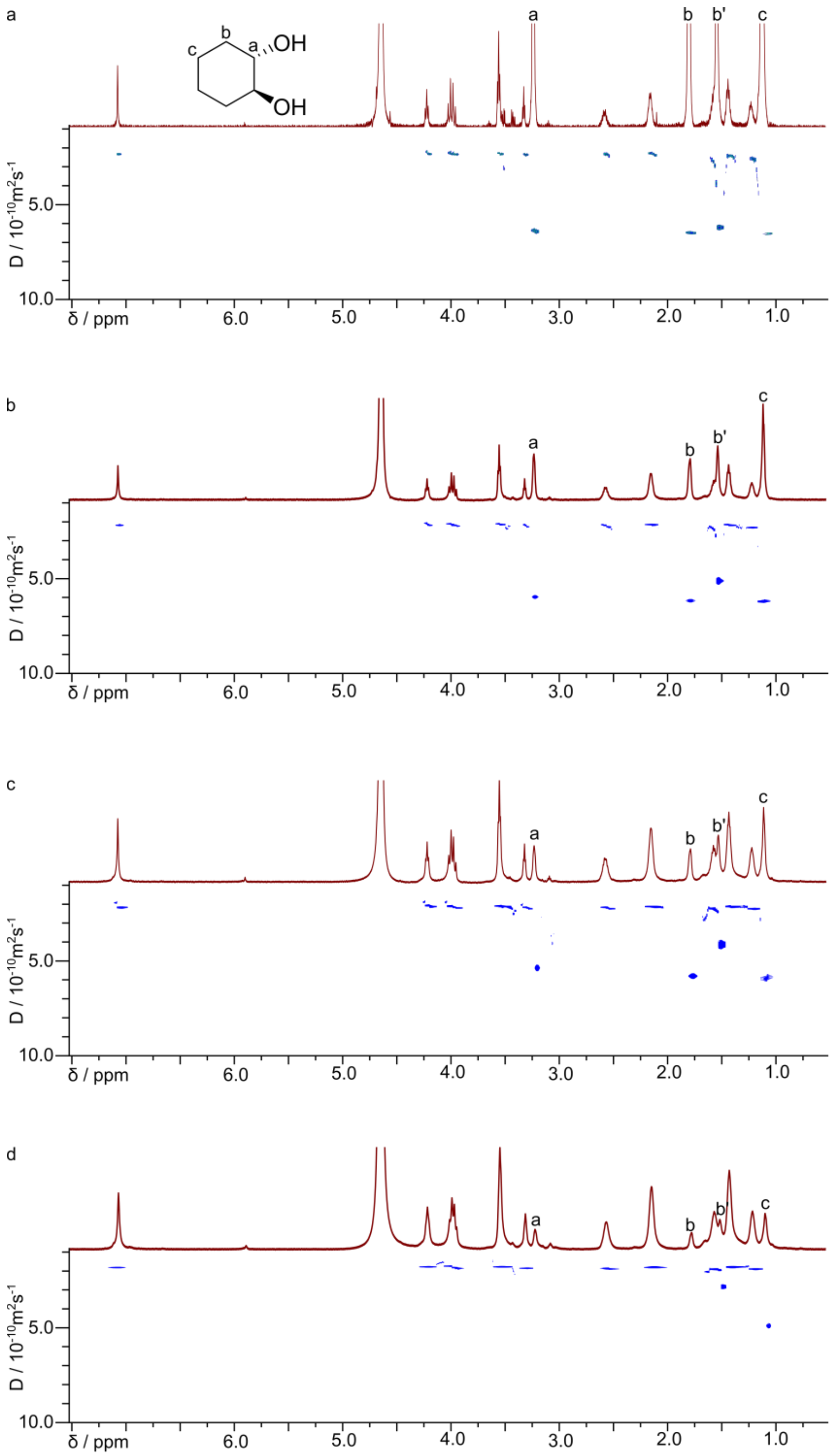

Figure S24. ^{1}H NMR and DOSY spectra for complexation of (1S,2S)-*trans*-1,2-cyclohexanediol **1**, (C = 10 mM) in (L-**ArgR**)$_2$ at pD 9.0 at different concentration a) 0.37 mM b) 1.9 mM c) 3.7 mM d) 10 mM (600 MHz, 298 K, D_2O, NaOD / DCl).

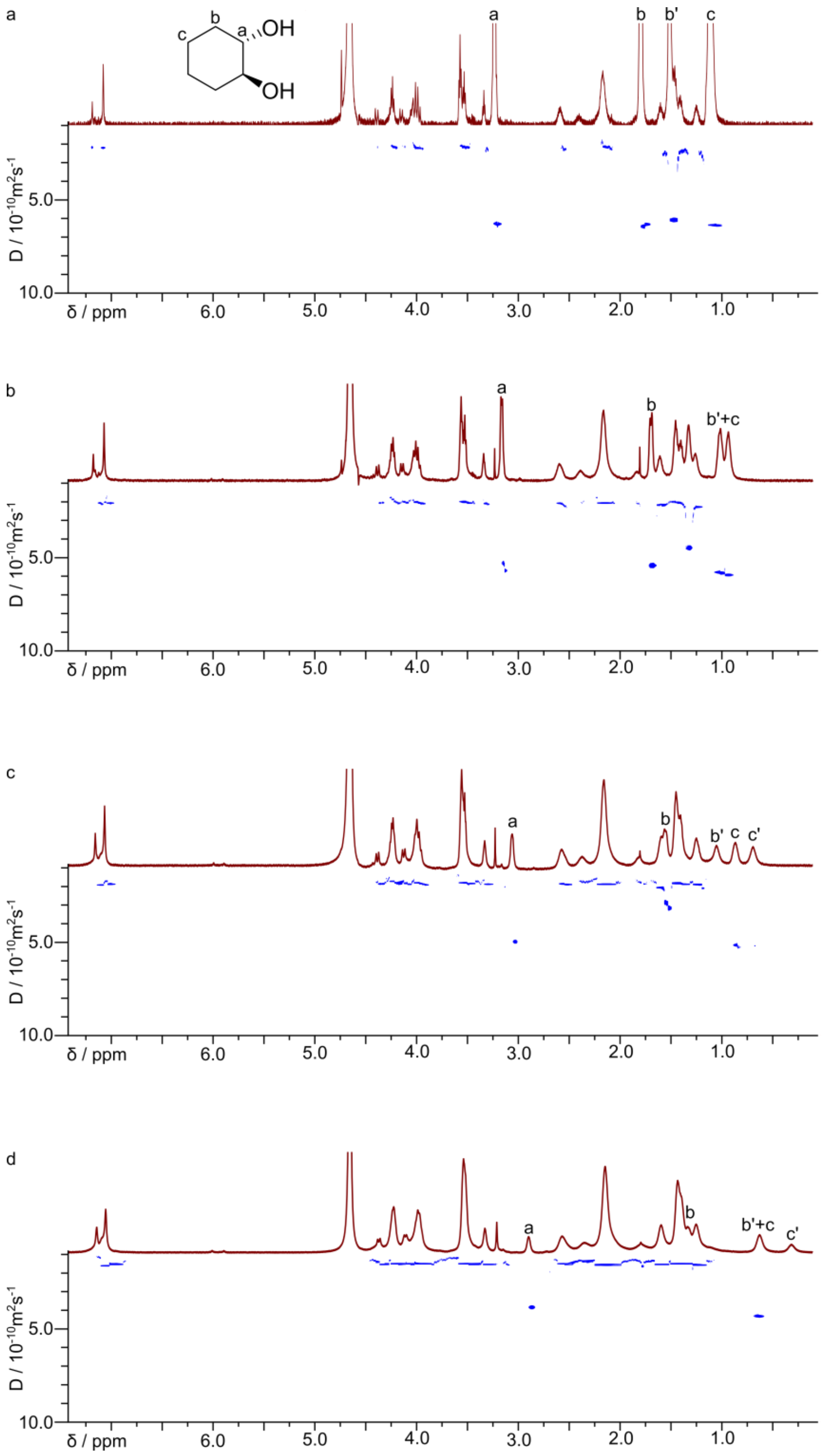

Figure S25. ^{1}H NMR and DOSY spectra for complexation of (1S,2S)-*trans*-1,2-cyclohexanediol **1,** (C = 10 mM) in D-**GluR**-L-**ArgR** at pD 9.0 at different concentration a) 0.37 mM b) 1.9 mM c) 3.7 mM d) 10 mM (600 MHz, 298 K, D_2O, NaOD / DCl).

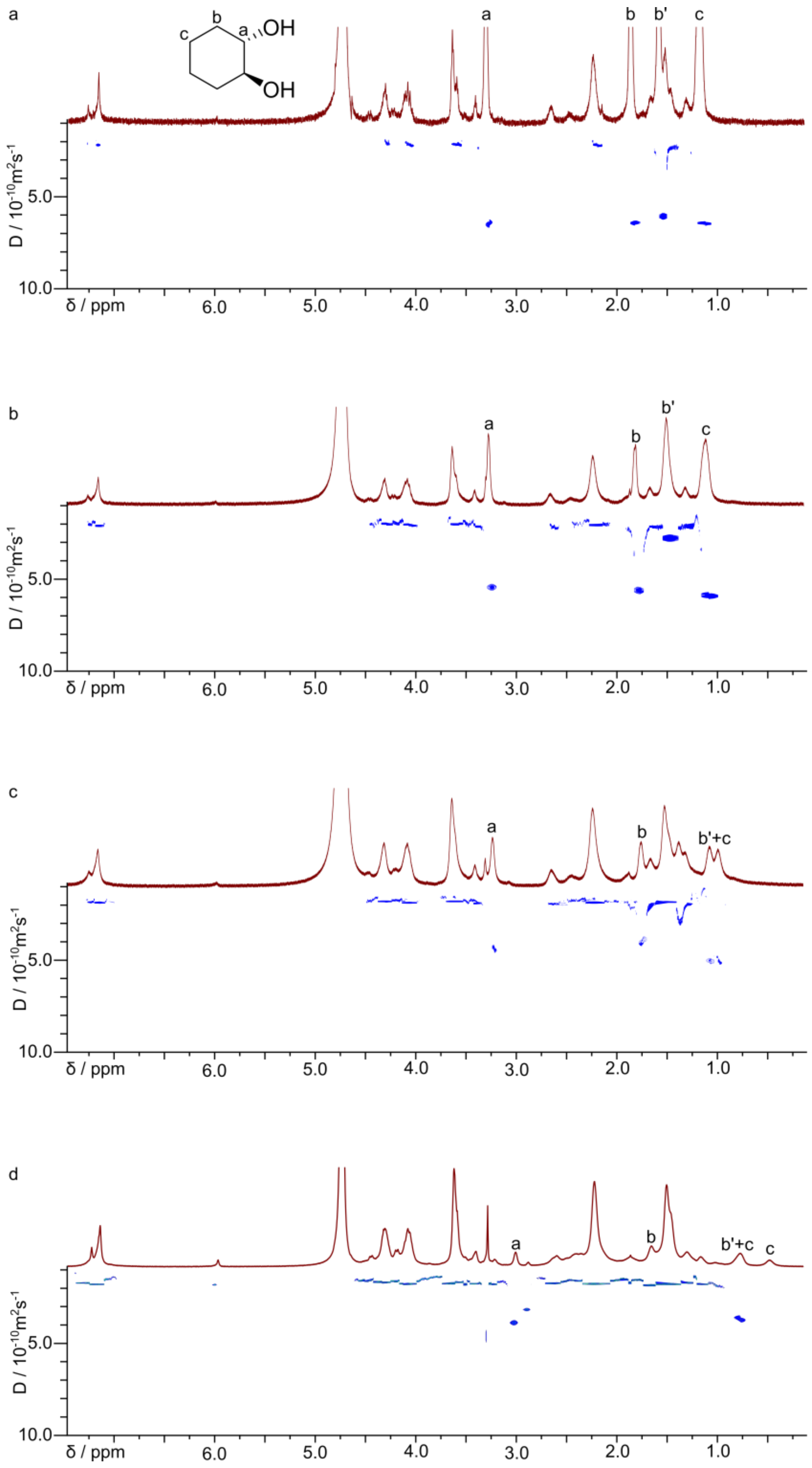

Figure S26. ^{1}H NMR and DOSY spectra for complexation of (1S,2S)-*trans*-1,2-cyclohexanediol **1**, (C = 10 mM) in L-**GluR**-L-**ArgR** at pD 9.0 at different concentration a) 0.37 mM b) 1.9 mM c) 3.7 mM d) 10 mM (600 MHz, 298 K, D_2O, NaOD / DCl).

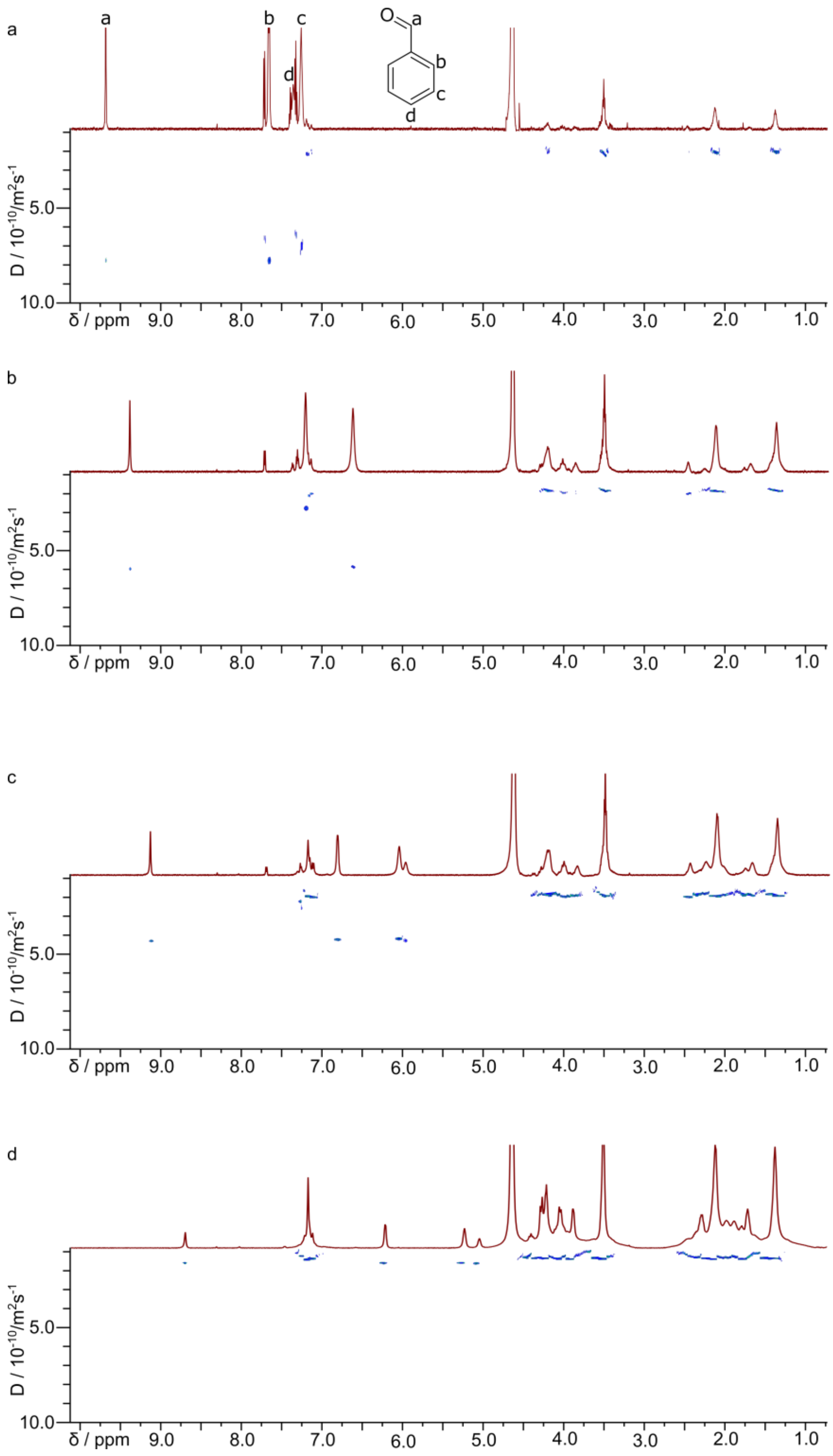

Figure S27. ^{1}H NMR and DOSY spectra for complexation of benzaldehyde **2** (saturation) in D_2O at pD 9.0 at different concentrations of (L-**GluR**)$_2$: a) 0.37 mM (integral-based ratio **2** : (L-**GluR**)$_2$ 1 : 0.05); b) 1.9 mM (integral-based ratio **2** : (L-**GluR**)$_2$ 1 : 0.1); c) 3.7 mM (integral-based ratio **2** : (L-**GluR**)$_2$ 1 : 0.2); d) 10.0 mM (integral-based ratio **2** : (L-**GluR**)$_2$ 1 : 0.6) (600 MHz, 298 K, D_2O, NaOD / DCl).

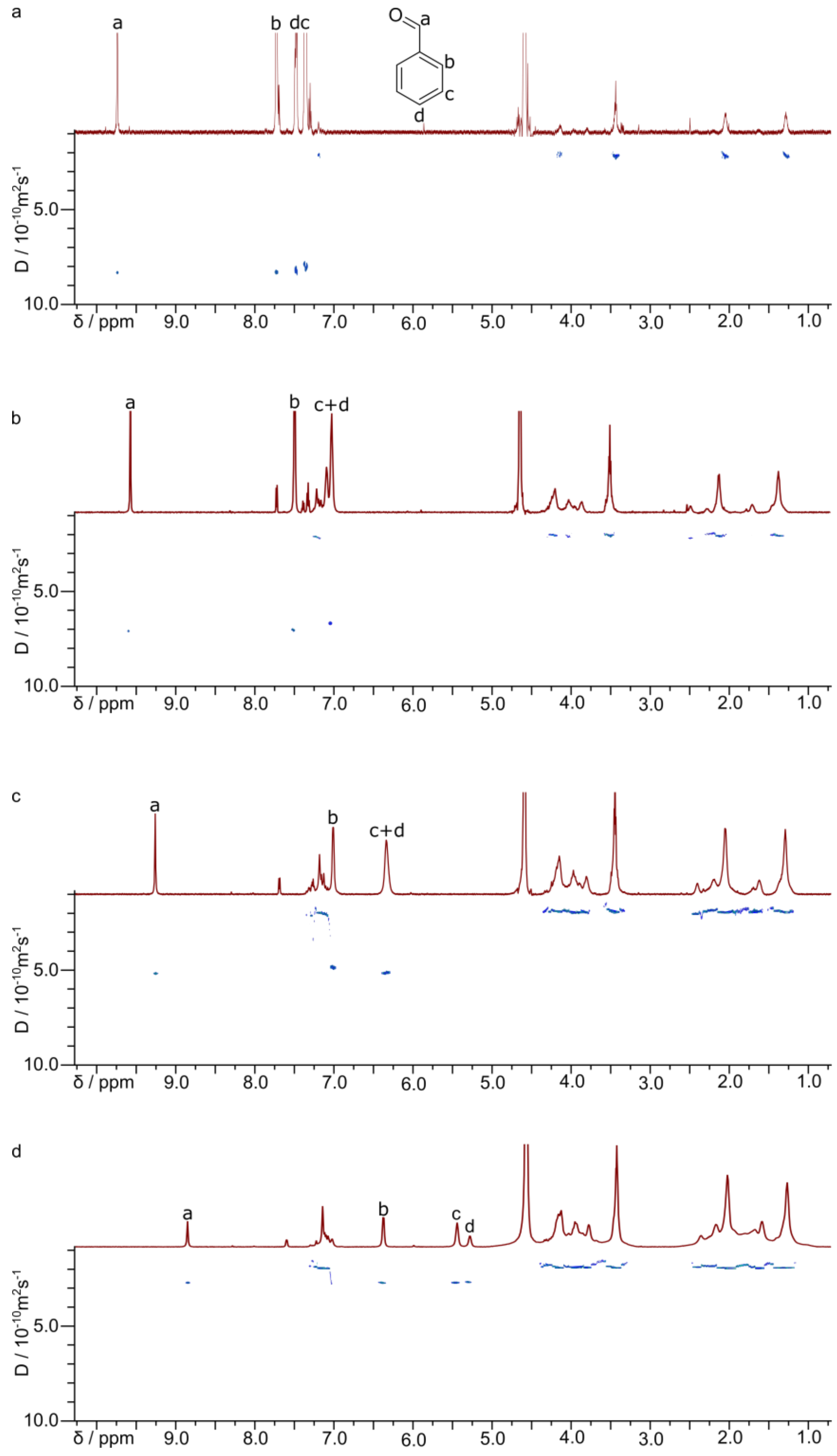

Figure S28. ^{1}H NMR and DOSY spectra for complexation of benzaldehyde, **2**, (saturation) in D_2O at pD 9.0 at different concentrations of D-**GluR**-L-**GluR**: a) 0.37 mM (integral-based ratio **2** : D-**GluR**-L-**GluR** 1 : 0.03); b) 1.9 mM (integral-based ratio **2** : D-**GluR**-L-**GluR** 1 : 0.1); c) 3.7 mM (integral-based ratio **2** : D-**GluR**-L-**GluR** 1 : 0.3); d) 10.0 mM (integral-based ratio **2** : D-**GluR**-L-**GluR** 1 : 0.6) (600 MHz, 298 K, D_2O, NaOD / DCl).

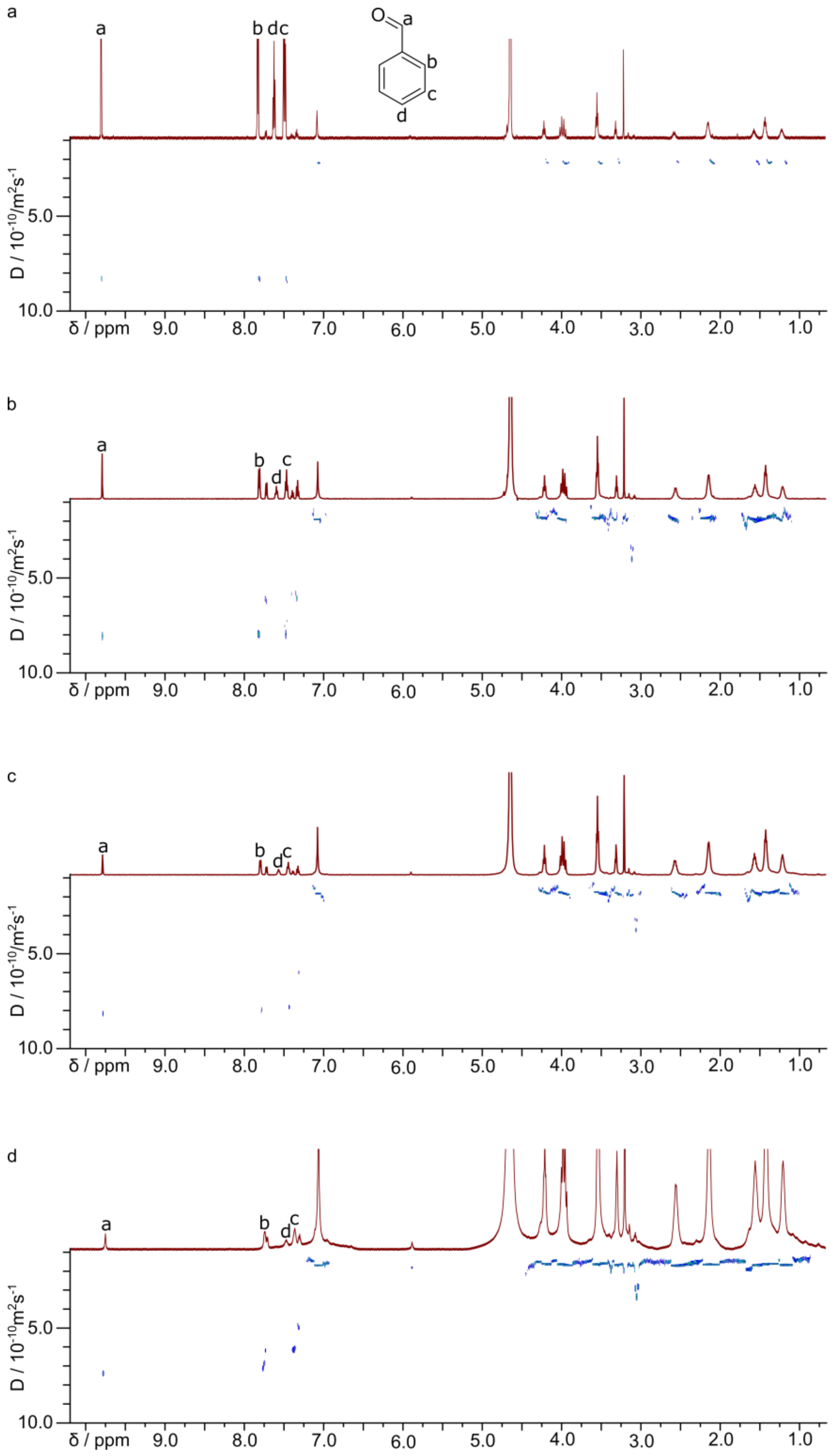

Figure S29. ^{1}H NMR and DOSY spectra for complexation of benzaldehyde, **2**, (saturation) in D_2O at pD 9.0 at different concentrations of (L-**ArgR**)$_2$: a) 0.33 mM (integral-based ratio **2** : (L-**ArgR**)$_2$ 1 : 0.04); b) 1.6 mM (integral-based ratio **2** : (L-**ArgR**)$_2$ 1 : 0.4); c) 3.3 mM (integral-based ratio **2 0** : (L-**ArgR**)$_2$ 1 : 0.7); d) 9.0 mM (integral-based ratio **2** : (L-**ArgR**)$_2$ 1 : 2.0) (600 MHz, 298 K, D_2O, NaOD / DCl).

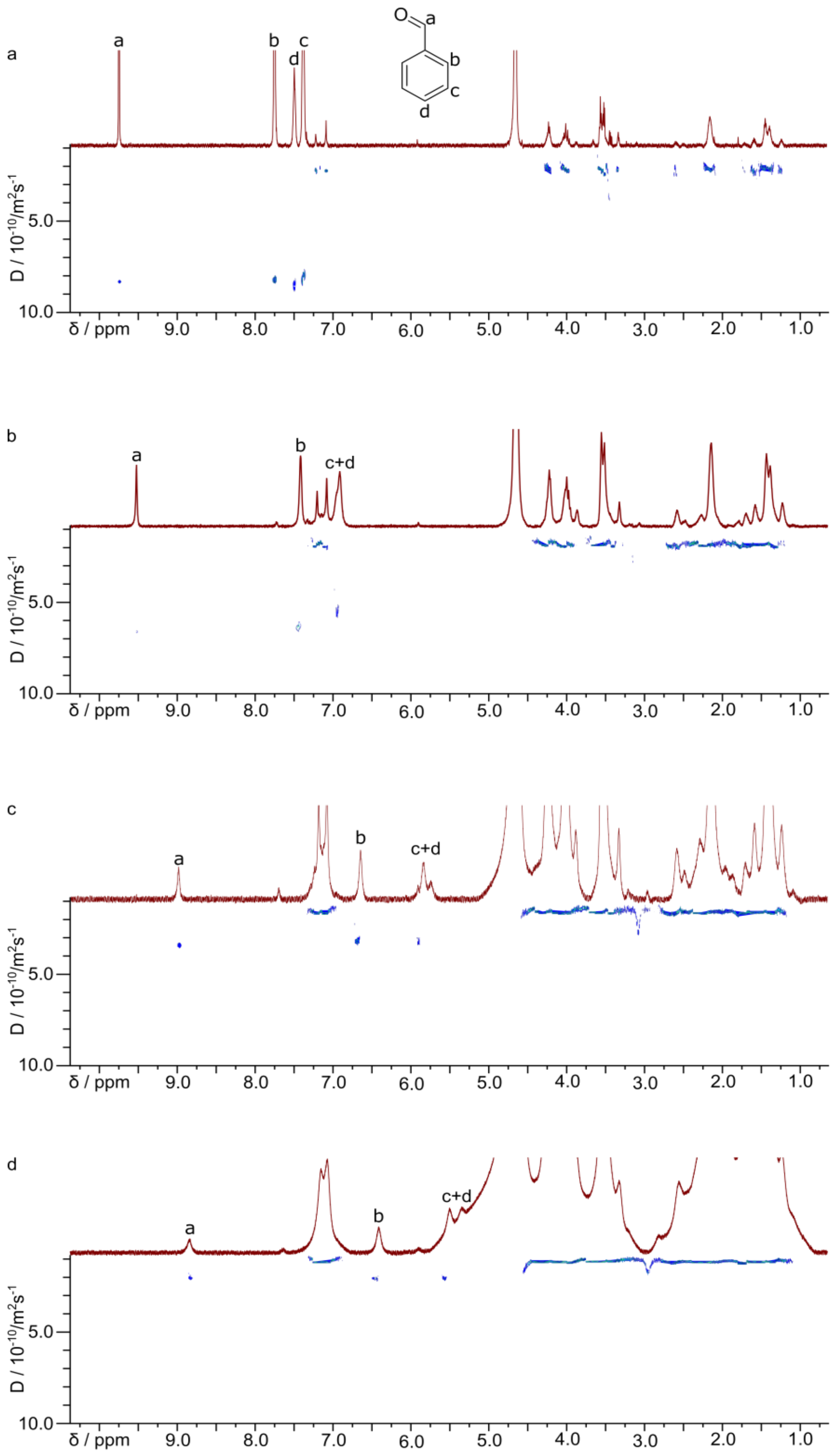

Figure S30. ^{1}H NMR and DOSY spectra for complexation of benzaldehyde **2** (saturation) in D_2O at pD 9.0 at different concentrations of D-**GluR**-L-**ArgR**: a) 0.35 mM (integral-based ratio **2** : D-**GluR**-L-**ArgR** 1:0.04); b) 1.7 mM (integral-based ratio **2** : D-**GluR**-L-**ArgR** 1 : 0.3); c) 3.5 mM (integral-based ratio **2** : L-**ArgR**-D-**GluR** 1 : 1.4); d) 9.6 mM (integral-based ratio **2** : D-**GluR**-L-**ArgR** 1 : 2.5) (600 MHz, 298 K, D_2O, NaOD / DCl).

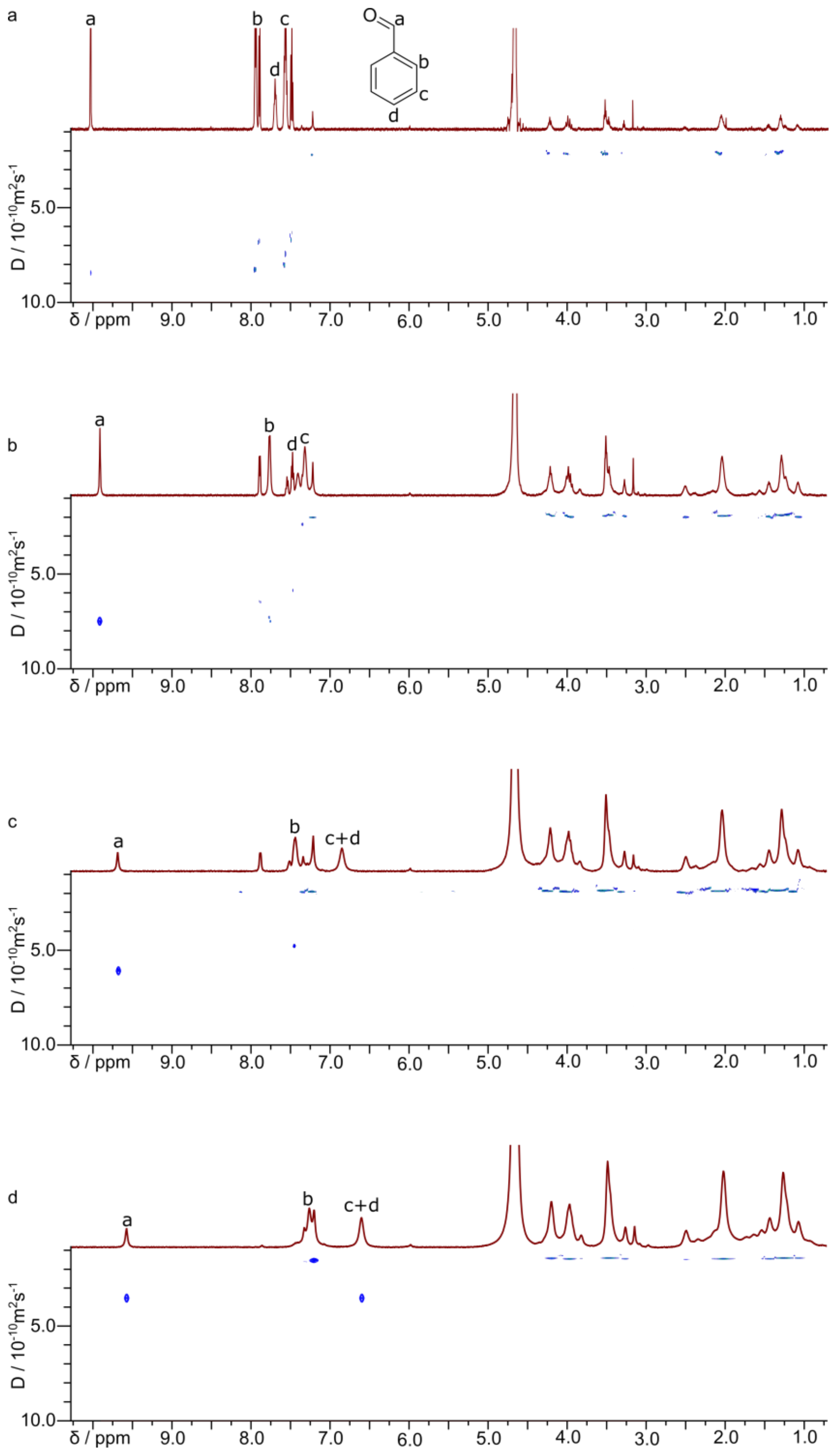

Figure S31. ^{1}H NMR and DOSY spectra for complexation of benzaldehyde, **2**, (saturation) in D_2O at pD 9.0 at different concentrations of L-**GluR**-L-**ArgR**: a) 0.35 mM (integral-based ratio **2** : L-**ArgR**-L-**GluR** 1 : 0.04); b) 1.8 mM (integral-based ratio **2** : L-**GluR**-L-**ArgR** 1 : 0.2); c) 3.5 mM (integral-based ratio **2** : L-**GluR**-L-**ArgR** 1 : 0.5); d) 9.5 mM (integral-based ratio **2** : L-**GluR**-L-**ArgR** 1 : 1.1) (600 MHz, 298 K, D_2O, NaOD / DCl).

2.3. Complexation of guests

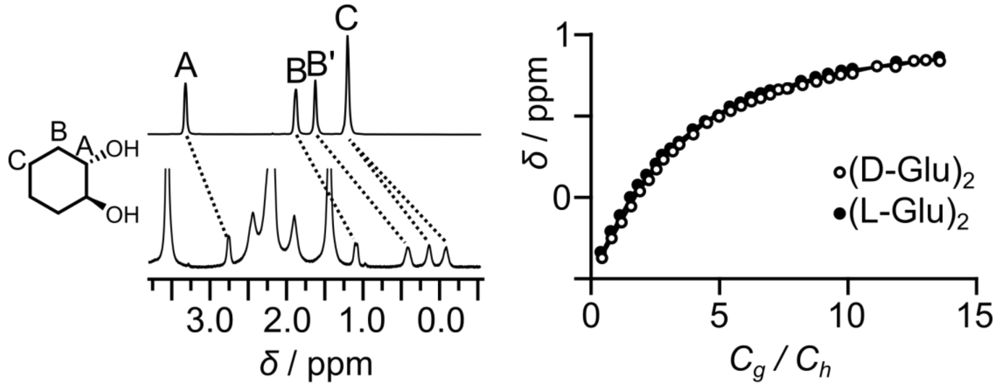

$$(\mathbf{GluR})_2 + \mathbf{1} \underset{}{\overset{K_1}{\rightleftharpoons}} (\mathbf{GluR})_2\mathbf{1}$$

$$(\mathbf{GluR})_2\mathbf{1} + \mathbf{1} \underset{}{\overset{K_2}{\rightleftharpoons}} (\mathbf{GluR})_2(\mathbf{1})_2$$

	K_1	σ_1	K_2	σ_2
(D-**GluR**)$_2$+**1**	4.84	0.03	1.85	0.03
(L-**GluR**)$_2$+**1**	4.81	0.04	1.73	0.03

Figure S32. ^{1}H NMR spectra for complexation of (1S,2S)-*trans*-1,2-cyclohexanediol **1** in (**GluR**)$_2$ (1 : 1 guest : host ratio) and the titration curves (400 MHz, 303 K, pD 4.8, D_2O, NaOD / DCl).

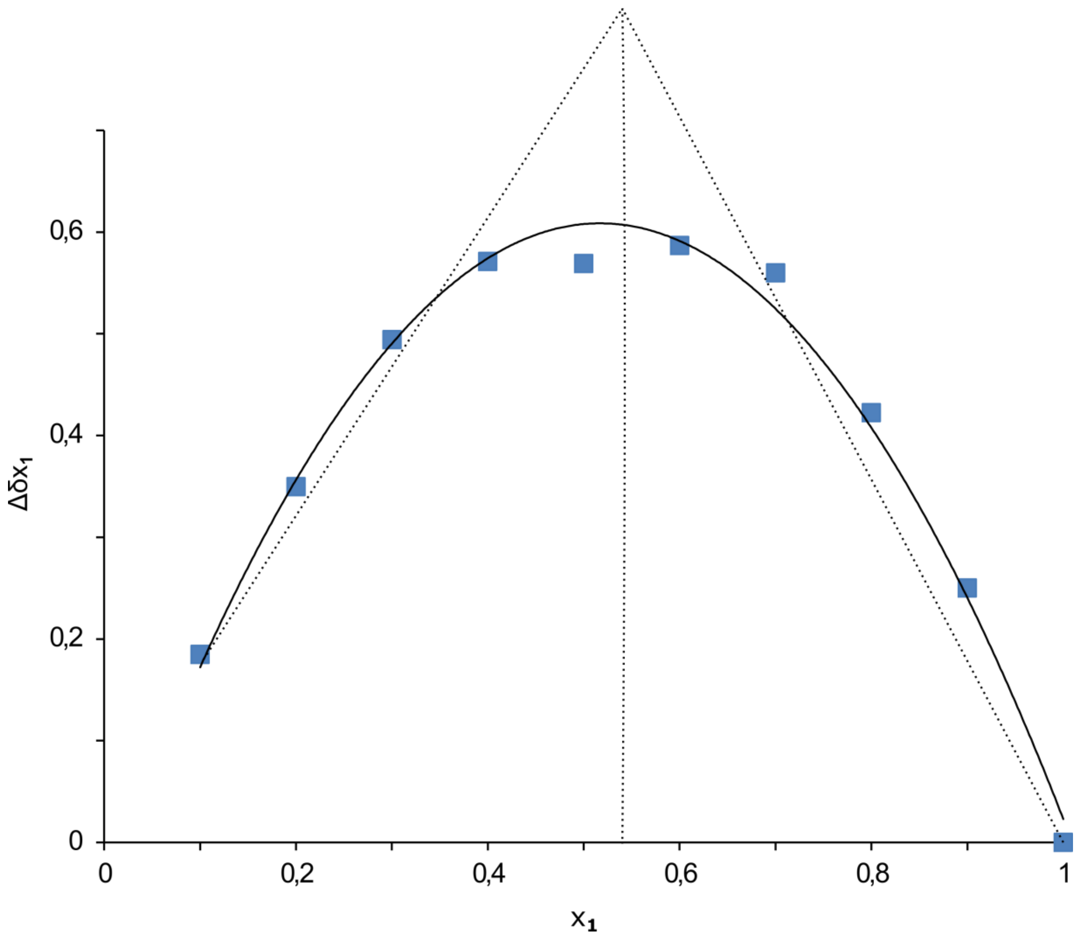

Figure S33. Job plot of (L-**GluR**)$_2$ and **1** (400 MHz, 303 K, pD 4.8, D_2O, NaOD / DCl).

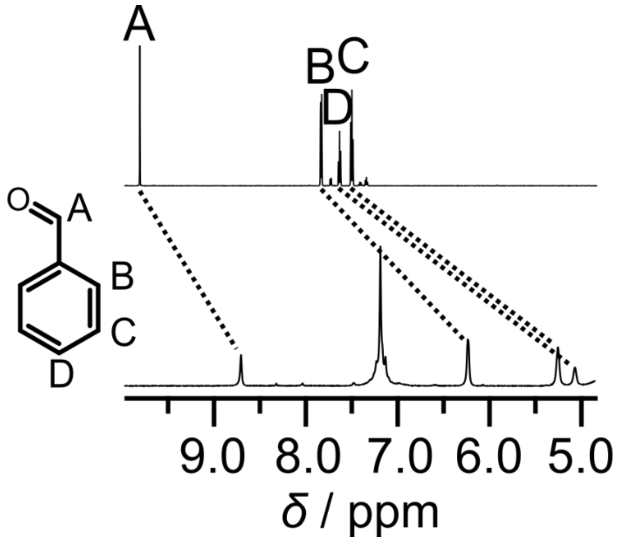

Figure S34. ^{1}H NMR spectra for complexation of benzaldehyde **2** in (L-**GluR**)$_2$ (1 : 1 guest : host ratio, 400 MHz, 303 K, pD 4.8, D_2O, NaOD / DCl).

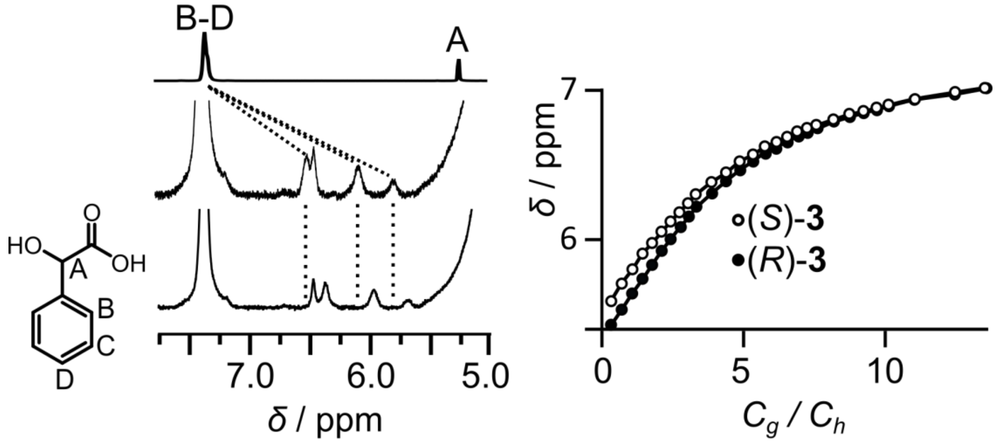

$$(\text{L-}\mathbf{ArgR})_2 + \mathbf{3} \underset{}{\overset{K_1}{\rightleftharpoons}} (\text{L-}\mathbf{ArgR})_2\mathbf{3}$$

$$(\text{L-}\mathbf{ArgR})_2\mathbf{3} + \mathbf{3} \underset{}{\overset{K_2}{\rightleftharpoons}} (\text{L-}\mathbf{ArgR})_2(\mathbf{3})_2$$

	K_1	σ_1	K_2	σ_2
(L-**ArgR**)+(*R*)-**3**	4.78	0.02	1.85	0.02
(L-**ArgR**)+(*S*)-**3**	4.34	0.03	1.77	0.03

Figure S35. ^{1}H NMR spectra for complexation of mandelic acid **3** in (L-**ArgR**)$_2$ (1 : 1 guest : host ratio, (*S*)-**3** middle, (*R*)-**3** bottom) and the titration curves (400 MHz, 303 K, pD 4.8, D_2O, NaOD / DCl).

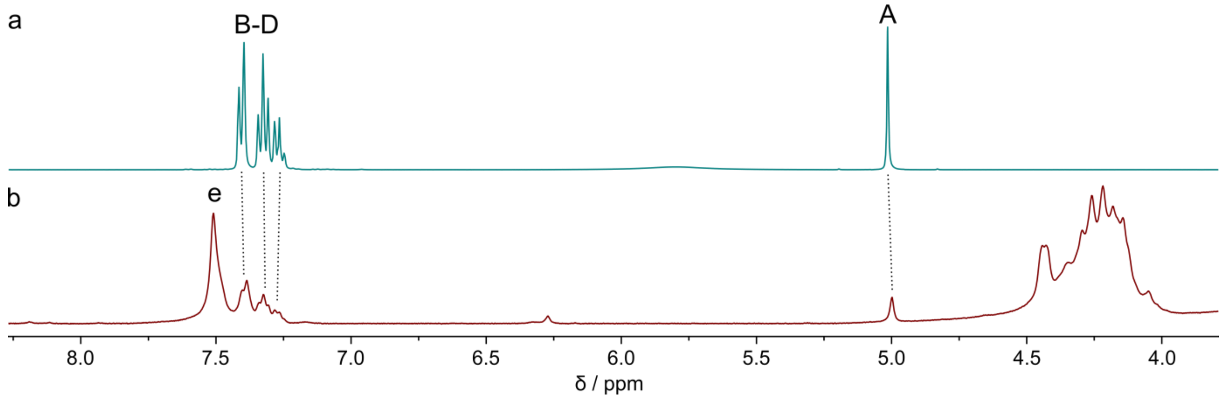

Figure S36. ^{1}H NMR spectra of a) mandelic acid **3** b) mandelic acid **3** in L-**GluR** (1 : 1 guest : host ratio) (400 MHz, 303 K, DMSO).

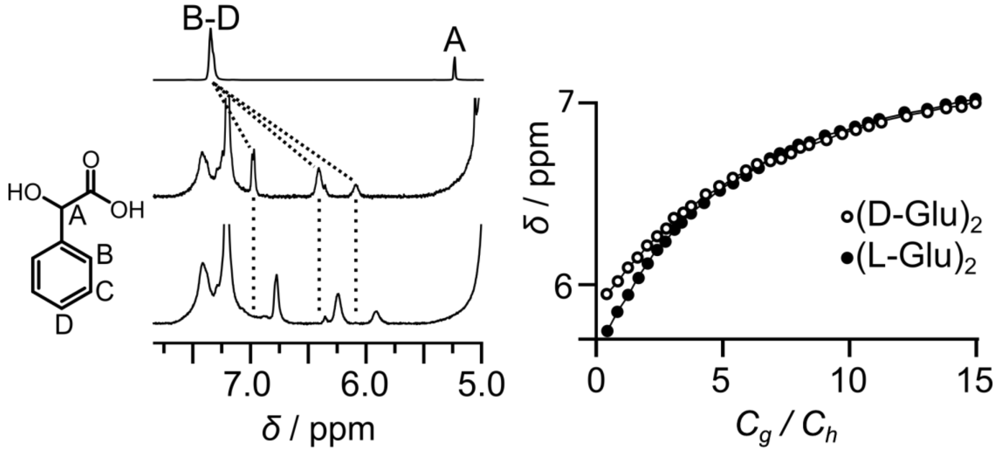

$$(\mathbf{GluR})_2 + \mathbf{3} \underset{}{\overset{K_1}{\rightleftharpoons}} (\mathbf{GluR})_2\mathbf{3}$$

$$(\mathbf{GluR})_2\mathbf{3} + \mathbf{3} \underset{}{\overset{K_2}{\rightleftharpoons}} (\mathbf{GluR})_2(\mathbf{3})_2$$

	K_1	σ_1	K_2	σ_2
(D-**GluR**)$_2$+(*R*)-**3**	4.85	0.02	1.25	0.02
(L-**GluR**)$_2$+(*R*)-**3**	4.20	0.03	1.80	0.03

Figure S37. ^{1}H NMR spectra for complexation of (*R*)-mandelic acid (*R*)-**3** in (**GluR**)$_2$ (1 : 1 guest : host ratio, (D-**GluR**)$_2$ middle, (L-**GluR**)$_2$ bottom) and the titration curves (400 MHz, 303 K, D_2O, pD 4.8, NaOD / DCl).

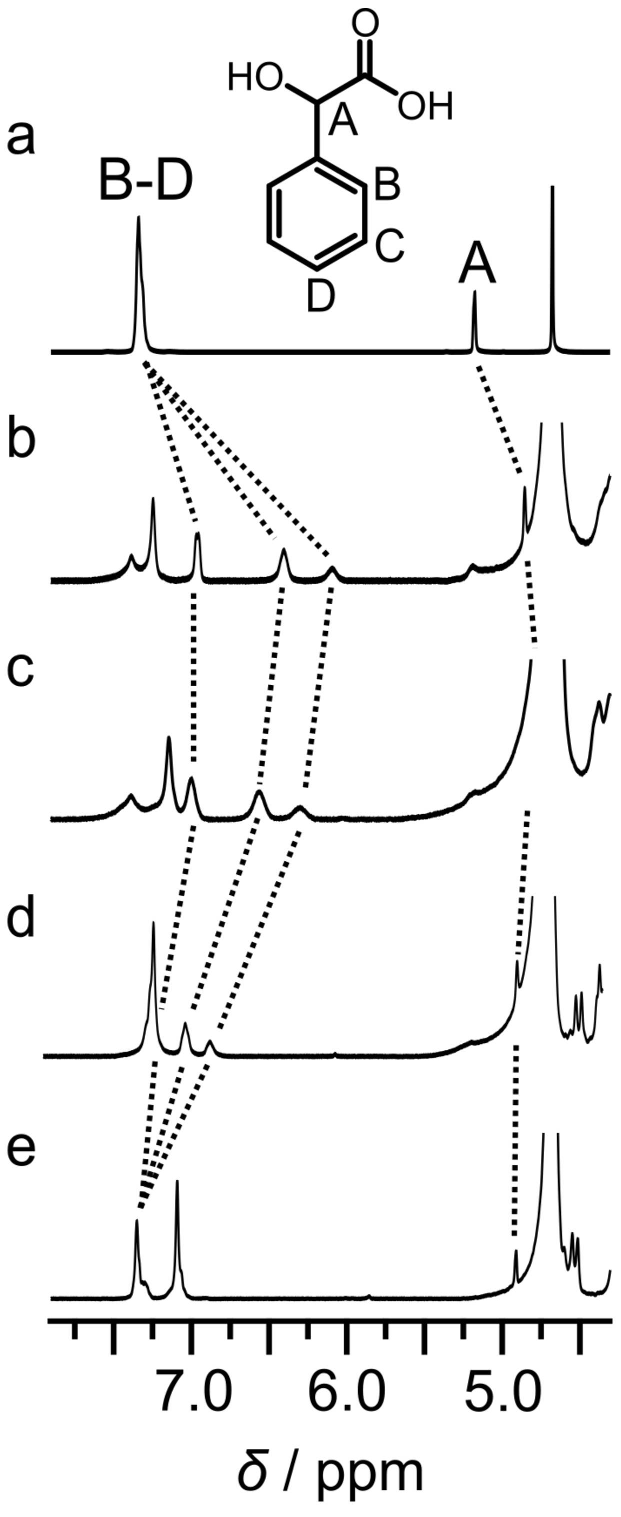

Figure S38. ^{1}H NMR spectra for complexation of (*R*)-mandelic acid (*R*)-**3** in (L-**GluR**)$_2$ (1 : 1 guest : host ratio) at various pD: a) (*R*)-**3** b) (L-**GluR**)$_2$ + (*R*)-**3** pD 4.5 c) (L-**GluR**)$_2$ + (*R*)-**3** pD 5.4 d) (L-**GluR**)$_2$ + (*R*)-**3** pD 9.1 e) (L-**GluR**)$_2$ + (*R*)-**3** pD 12.0 (400 MHz, 303 K, D_2O, NaOD / DCl).

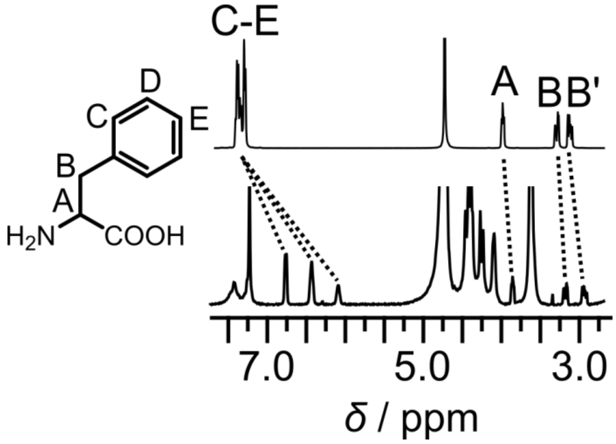

Figure S39. ^{1}H NMR spectra for complexation of phenylalanine **4** in (L-**GluR**)$_2$ (1 : 1 guest : host ratio, 400 MHz, 303 K, pD 4.8, D_2O, NaOD / DCl).

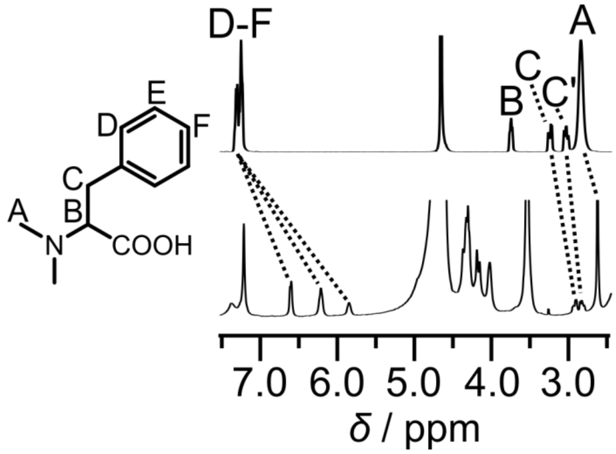

Figure S40. ^{1}H NMR spectra for complexation of *N*,*N*-dimethylphenylalanine **5** in (L-**GluR**)$_2$ (1 : 1 guest : host ratio, 400 MHz, 303 K, pD 4.8, D_2O, NaOD / DCl).

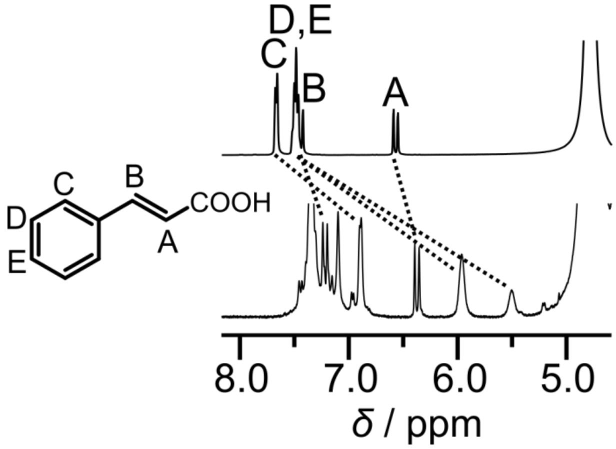

Figure S41. ^{1}H NMR spectra for complexation of cinnamic acid **6** in (L-**GluR**)$_2$ (1 : 1 guest : host ratio, 400 MHz, 303 K, pD 4.8, D_2O, NaOD / DCl).

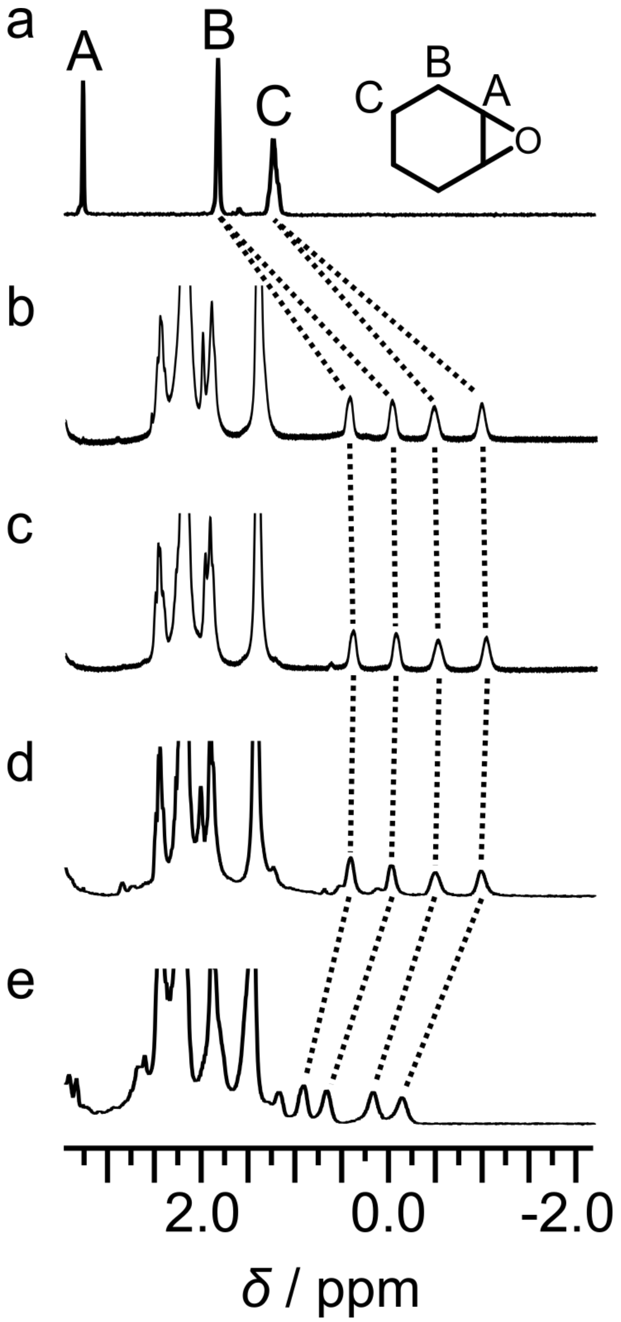

Figure S42. ^{1}H NMR spectra for complexation of epoxycyclohexane **7** in (L-**GluR**)$_2$: a) epoxycyclohexane; b) epoxycyclohexane + (L-**GluR**)$_2$ at pD 4.7 c) epoxycyclohexane + (L-**GluR**)$_2$ at pD 7.0 d) epoxycyclohexane + (L-**GluR**)$_2$ at pD 8.5 e) epoxycyclohexane + (L-**GluR**)$_2$ at pD 12.0 (1:1 guest : host ratio, 400 MHz, 303 K, D_2O, NaOD / DCl).

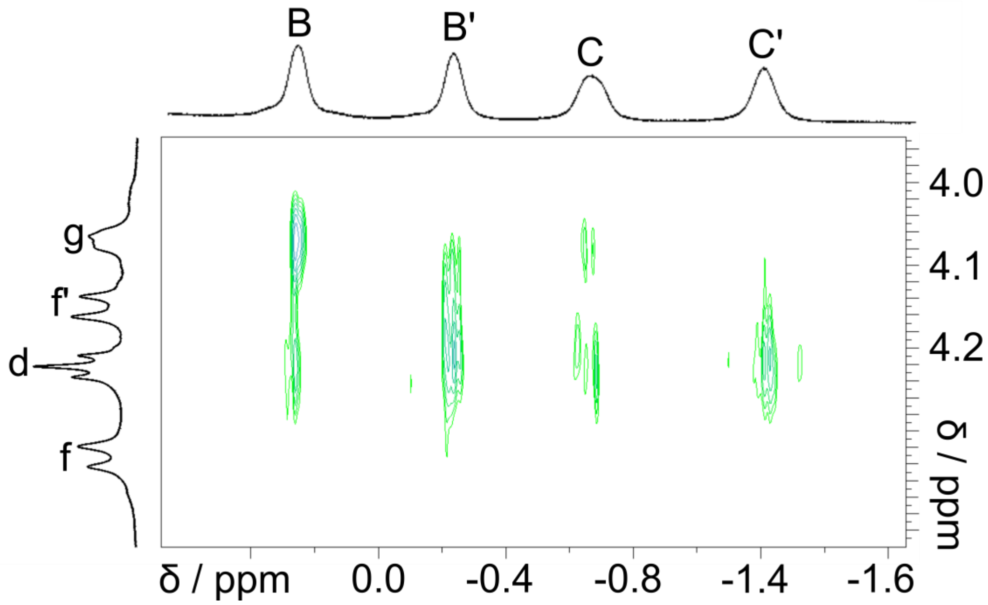

Figure S43. Partial ROESY spectrum of epoxycyclohexane in (L-**GluR**)$_2$ (600 MHz, 298 K, pD 4.8, D_2O, NaOD / DCl).

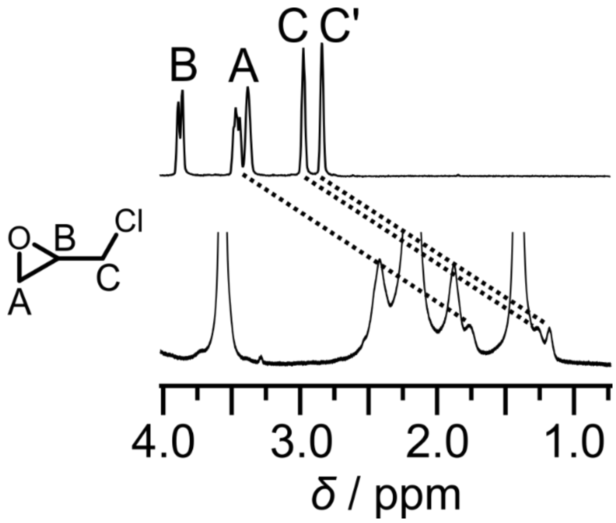

Figure S44. ^{1}H NMR spectra for complexation of epichlorohydrin **8** in (L-**GluR**)$_2$ (1 : 1 guest : host ratio, 400 MHz, 303 K, pD 4.8, D_2O, NaOD / DCl).

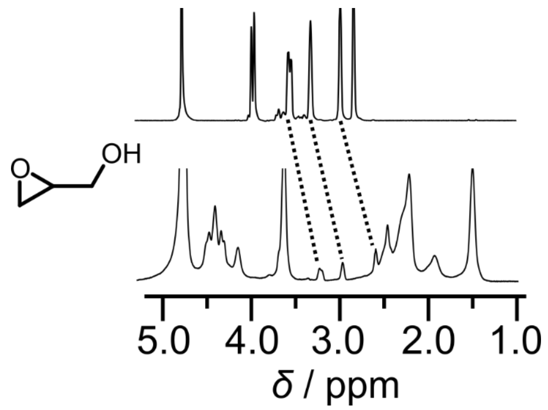

Figure S45. ^{1}H NMR spectra for complexation of glycidiol **9** in (L-**GluR**)$_2$ (1 : 1 guest : host ratio, 400 MHz, 303 K, pD 4.8, D_2O, NaOD / DCl).

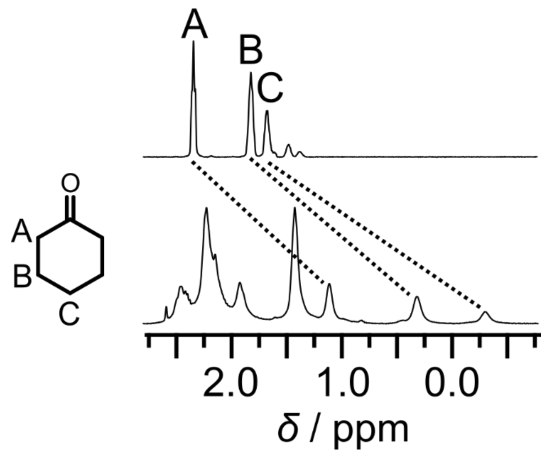

Figure S46. ^{1}H NMR spectra for complexation of cyclohexanone **10** in (L-**GluR**)$_2$ (1 : 1 guest : host ratio, 400 MHz, 303 K, pD 4.8, D_2O, NaOD / DCl).

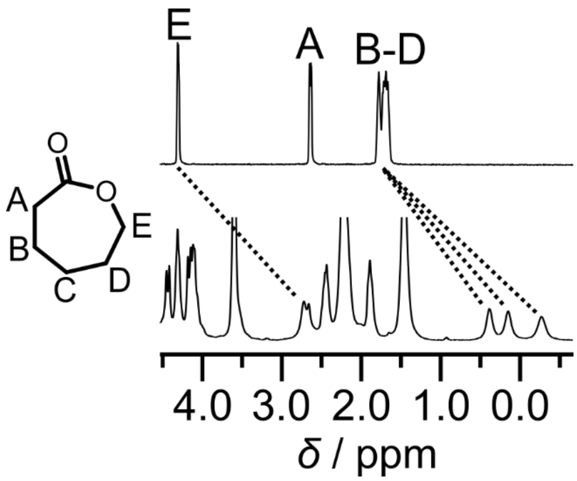

Figure S47. ^{1}H NMR spectra for complexation of caprolactone **11** in (L-**GluR**)$_2$ (1 : 1 guest : host ratio, 400 MHz, 303 K, pD 4.8, D_2O, NaOD / DCl).

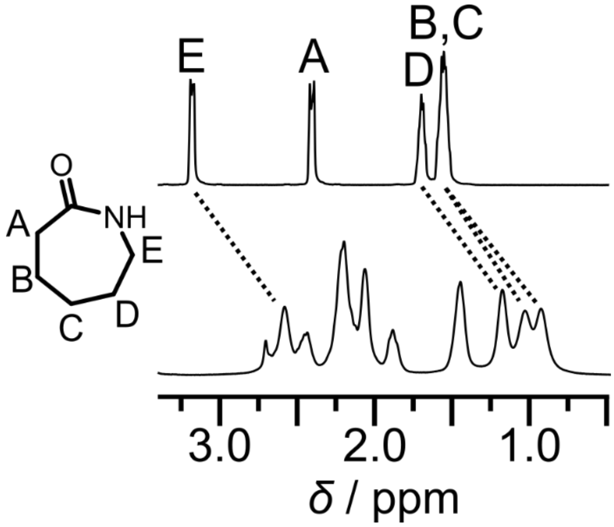

Figure S48. ^{1}H NMR spectra for complexation of caprolactam **12** in (L-**GluR**)$_2$ (1 : 1 guest : host ratio, 400 MHz, 303 K, pD 4.8, D_2O, NaOD / DCl).

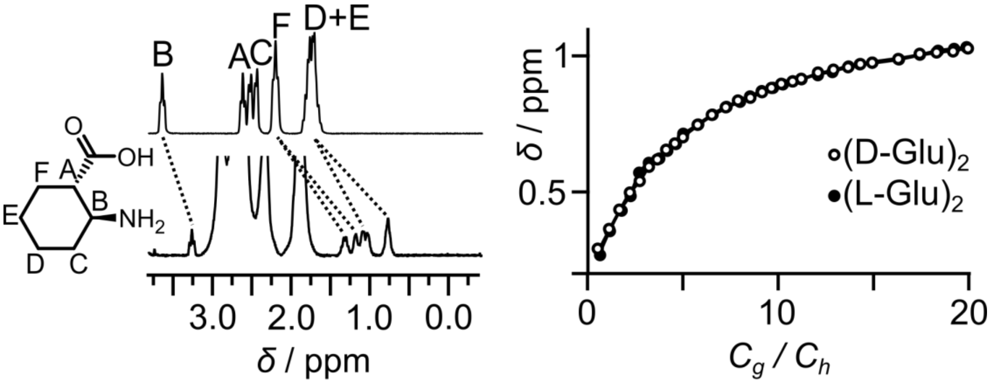

$$(\mathbf{GluR})_2 + \mathbf{13} \underset{}{\overset{K_1}{\rightleftharpoons}} (\mathbf{GluR})_2\mathbf{13}$$

$$(\mathbf{GluR})_2\mathbf{13} + \mathbf{13} \underset{}{\overset{K_2}{\rightleftharpoons}} (\mathbf{GluR})_2(\mathbf{13})_2$$

	K_1	σ_1	K_2	σ_2
(D-**GluR**)$_2$+(*R*)-**13**	4.80	0.02	1.46	0.02
(L-**GluR**)$_2$+(*R*)-**13**	4.80	0.05	1.32	0.05

Figure S49. ^{1}H NMR spectra for complexation of (1*S*,2*S*)-2-aminocyclohexanecarboxylic acid **13** in (**GluR**)$_2$ (1 : 1 guest : host ratio) and the titration curves (400 MHz, 303 K, pD 4.8, D_2O).

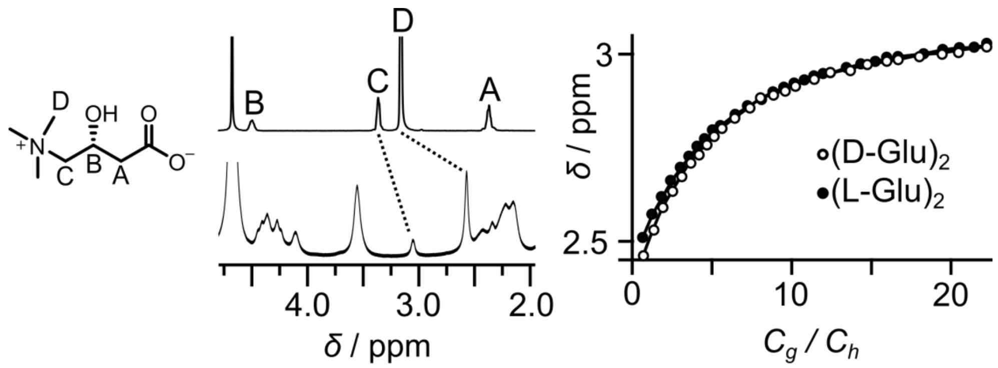

$$(\mathbf{GluR})_2 + \mathbf{14} \underset{}{\overset{K_1}{\rightleftharpoons}} (\mathbf{GluR})_2\mathbf{14}$$

$$(\mathbf{GluR})_2\mathbf{14} + \mathbf{14} \underset{}{\overset{K_2}{\rightleftharpoons}} (\mathbf{GluR})_2(\mathbf{14})_2$$

	K_1	σ_1	K_2	σ_2
(D-**GluR**)$_2$+(*R*)-**14**	4.77	0.03	1.54	0.03
(L-**GluR**)$_2$+(*R*)-**14**	4.99	0.02	1.43	0.02

Figure S50. ^{1}H NMR spectra for complexation of L-carnitine **14** in (**GluR**)$_2$ (1 : 1 guest : host ratio) and the titration curves (400 MHz, 303 K, pD 4.8, D_2O).

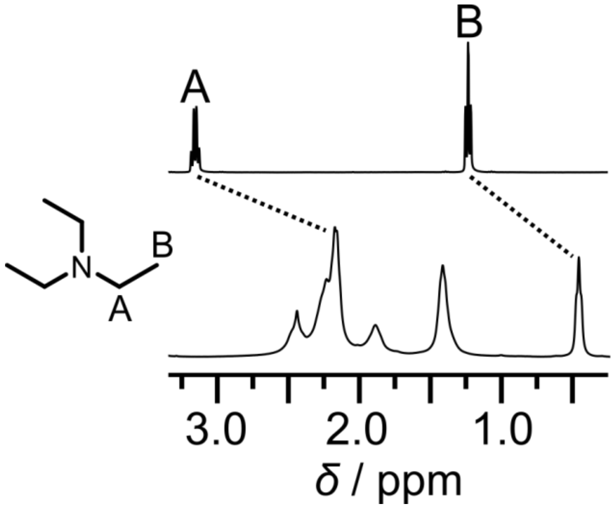

Figure S51. ^{1}H NMR spectra for complexation of trimethylamine **15** in (L-**GluR**)$_2$ (1 : 1 guest : host ratio, 400 MHz, 303 K, pD 4.8, D_2O, NaOD / DCl).

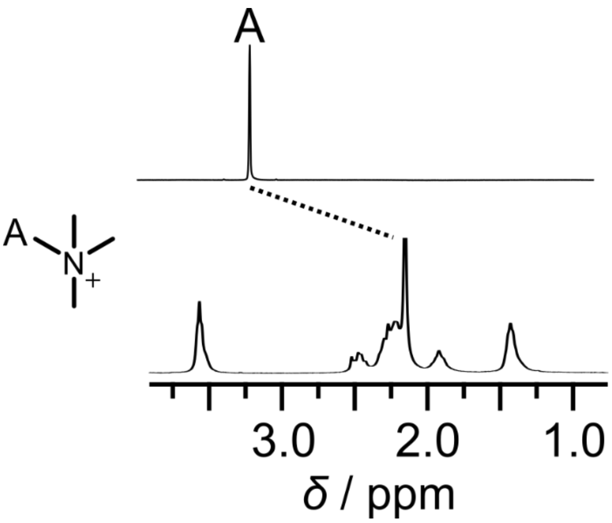

Figure S52. ^{1}H NMR spectra for complexation of tertamethylammonium salt **16** in (L-**GluR**)$_2$ (1 : 1 guest : host ratio, 400 MHz, 303 K, pD 4.8, D_2O, NaOD / DCl).

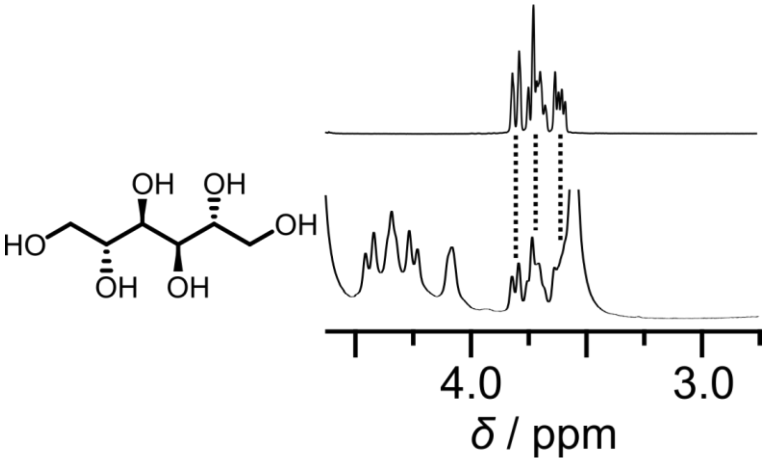

Figure S53. ^{1}H NMR spectra for complexation of mannitol in (L-**GluR**)$_2$ (1 : 1 guest : host ratio, 400 MHz, 303 K, pD 4.8, D_2O, NaOD / DCl).

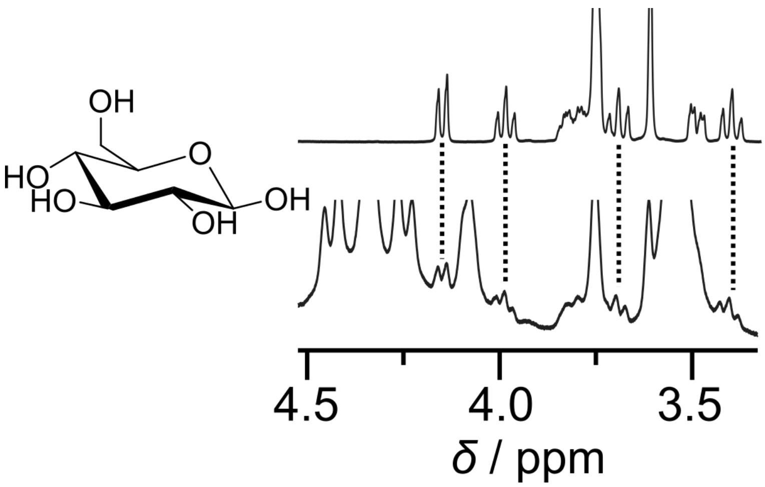

Figure S54. ^{1}H NMR spectra for complexation of glucose in (L-**GluR**)$_2$ (1 : 1 guest : host ratio, 400 MHz, 303 K, pD 4.8, D_2O, NaOD / DCl).

3. UV and ECD spectra

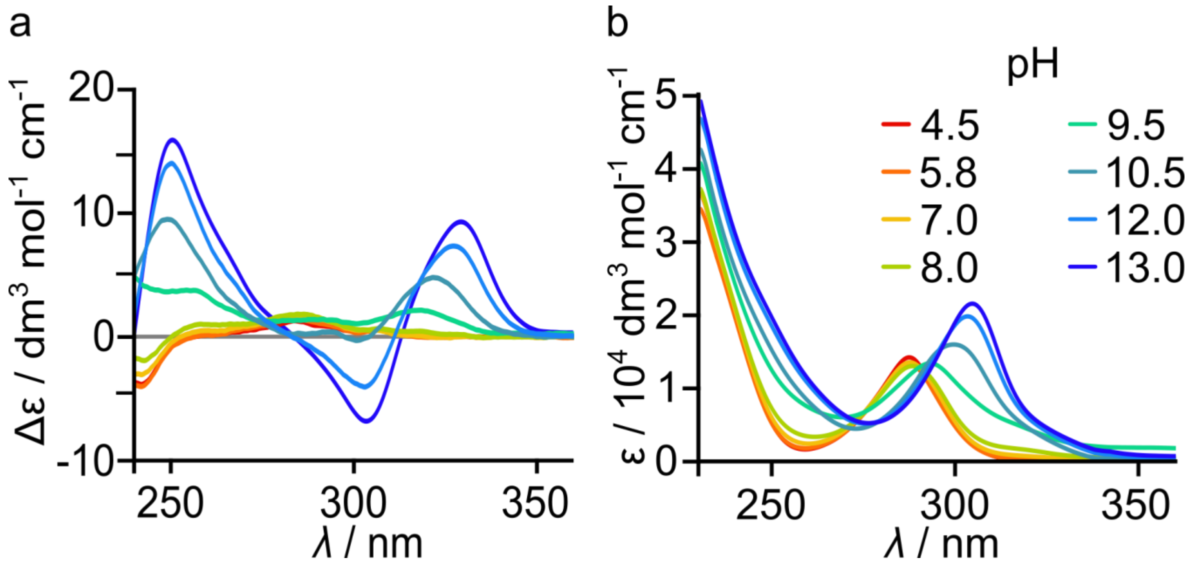

,

Figure S55. pH-dependent a) ECD and b) UV spectra of L-**GluR** (C = 3.1 mM concentration calculated per cavitand, water, pH set by NaOH / HCl).

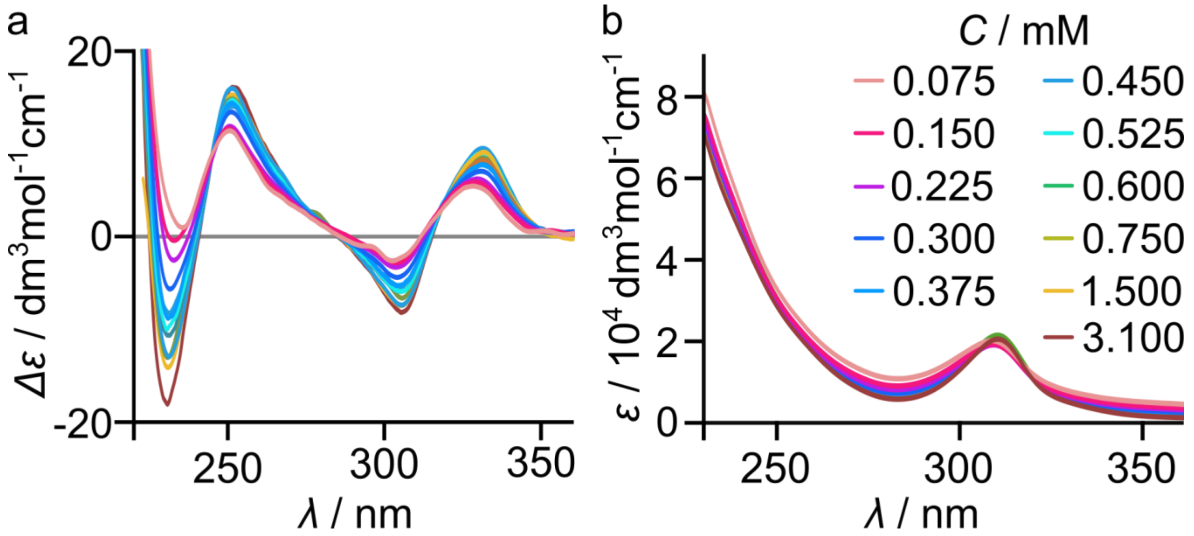

Figure S56. a) ECD and b) UV spectra of titration of L-**GluR** (concentration calculated per cavitand, water, pH = 11.90 pH set by NaOH / HCl).

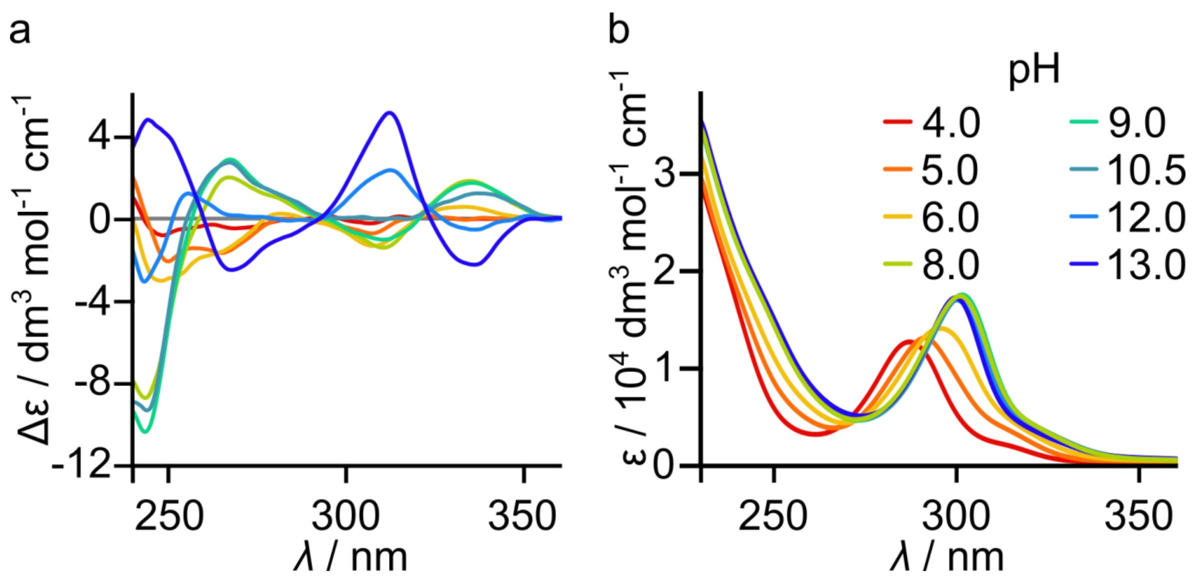

Figure S57. pH-dependent a) ECD and b) UV spectra of L-**ArgR** (C = 3.1 mM concentration calculated per cavitand, water, pH set by NaOH / HCl).

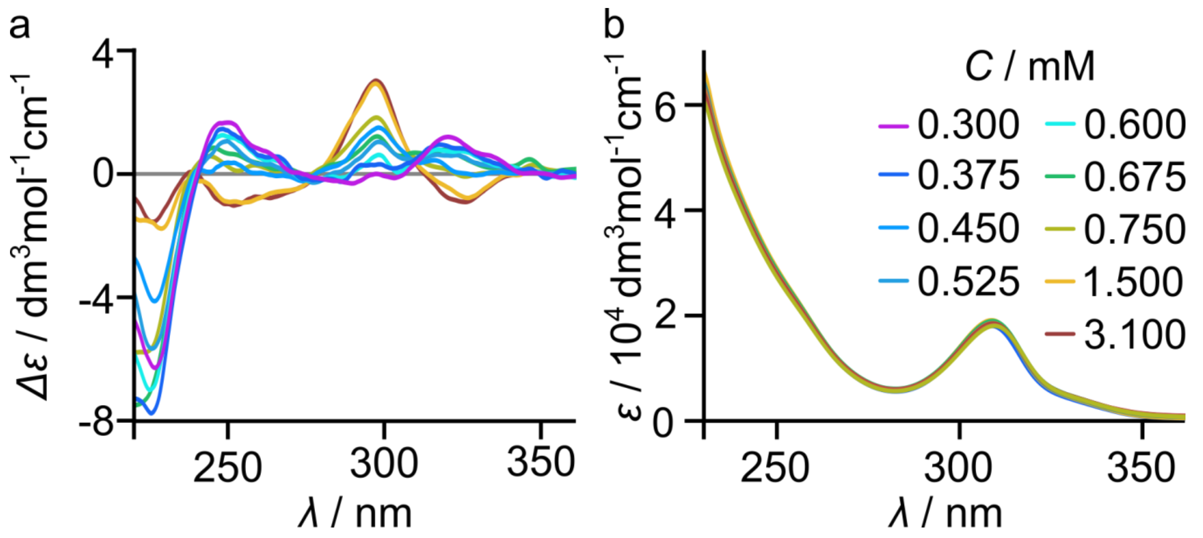

Figure S58. a) ECD and b) UV spectra of titration of L-**ArgR** (concentration calculated per cavitand, water, pH = 11.90 pH set by NaOH / HCl).

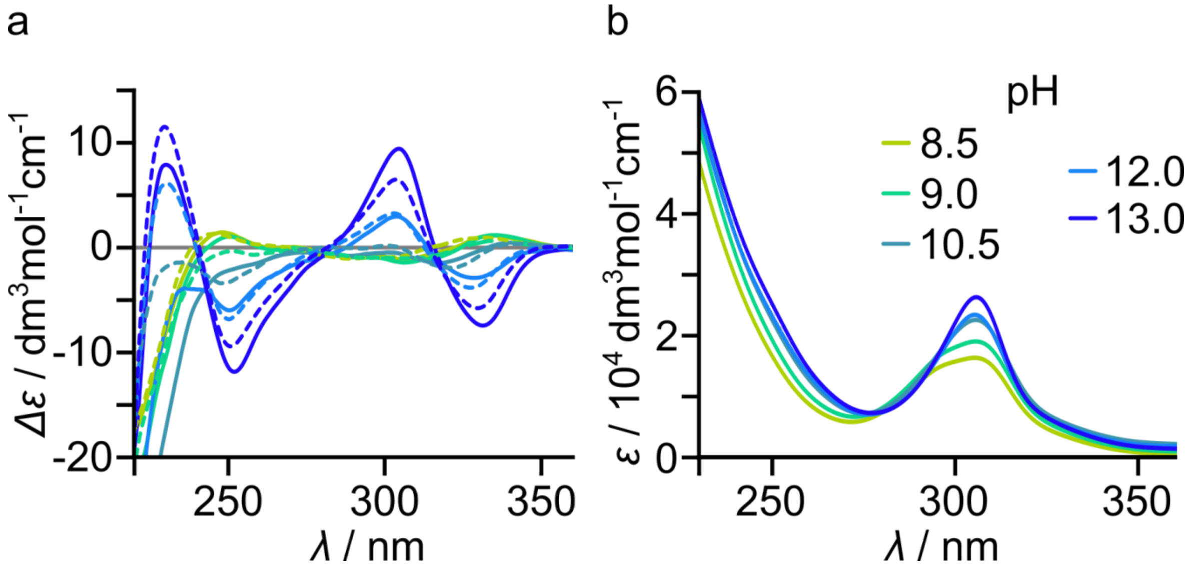

Figure S59. pH-dependent a) ECD and b) UV spectra of D-**GluR**-D-**ArgR** (solid lines – experimental spectra, dashed lines – a weighted mathematical sum of the components at given pH) ($C_{(\text{L-}\mathbf{ArgR})}$ = 1.85 mM, $C_{(\text{D-}\mathbf{GluR})}$ = 1.85 mM, water, pH set by NaOH / HCl).

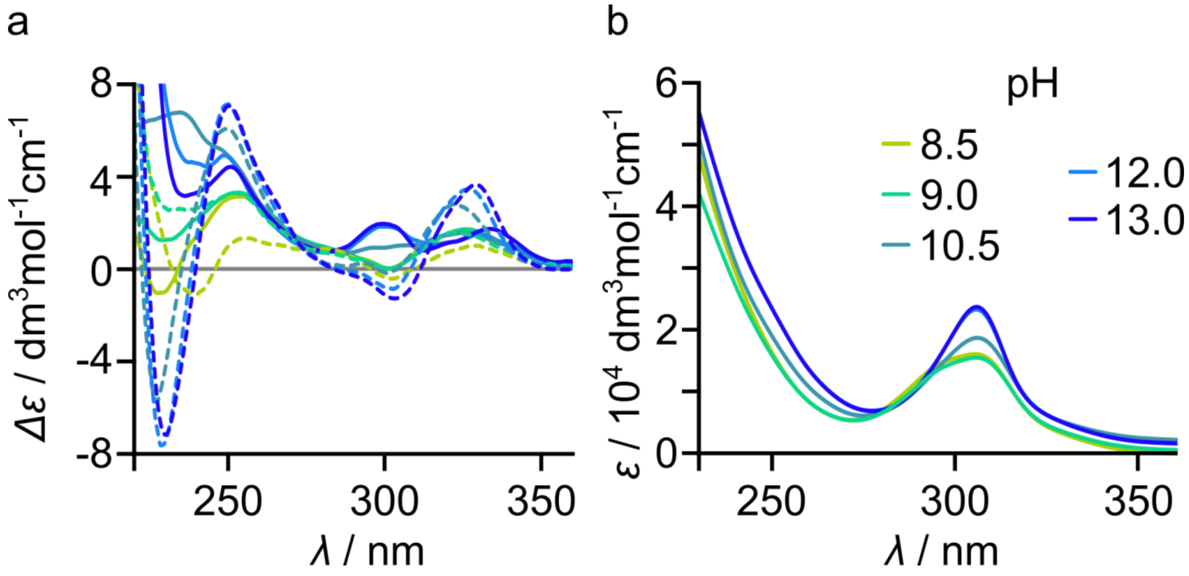

Figure S60. pH-dependent a) ECD and b) UV spectra of L-**GluR**-L-**ArgR** (solid lines – experimental spectra, dashed lines – a weighted mathematical sum of the components at given pH) ($C_{(\text{L-}\mathbf{ArgR})}$ = 1.85 mM, $C_{(\text{L-}\mathbf{GluR})}$ = 1.85 mM, water, pH set by NaOH / HCl).

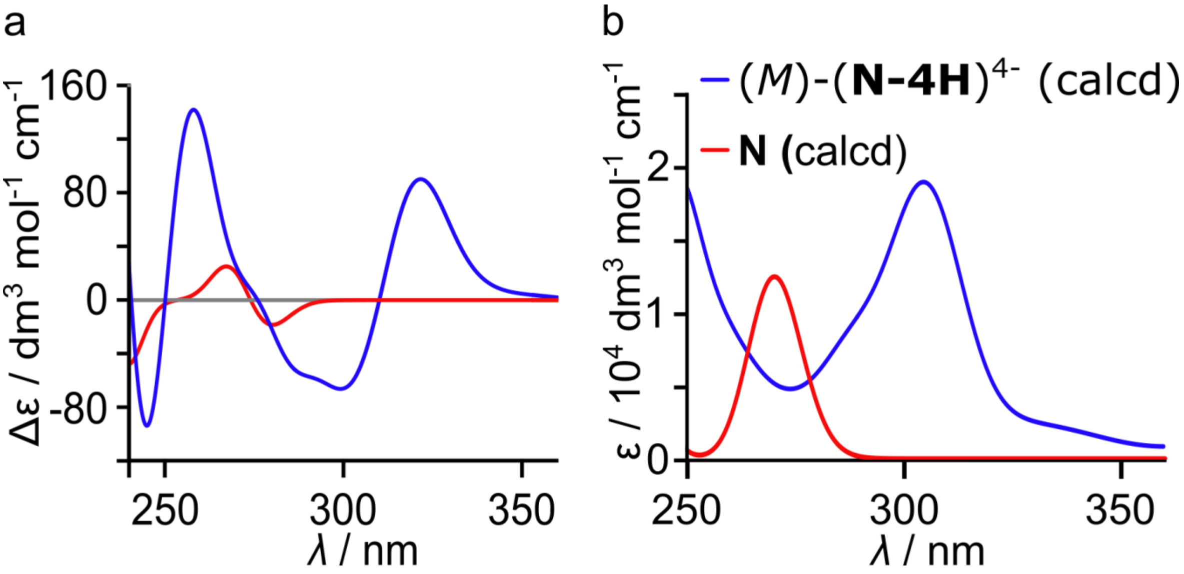

Figure S61. Calculated a) ECD and b) UV spectra of (*M*)-(**N-4H**)$^{4-}$ and **N**.

4. IR spectra

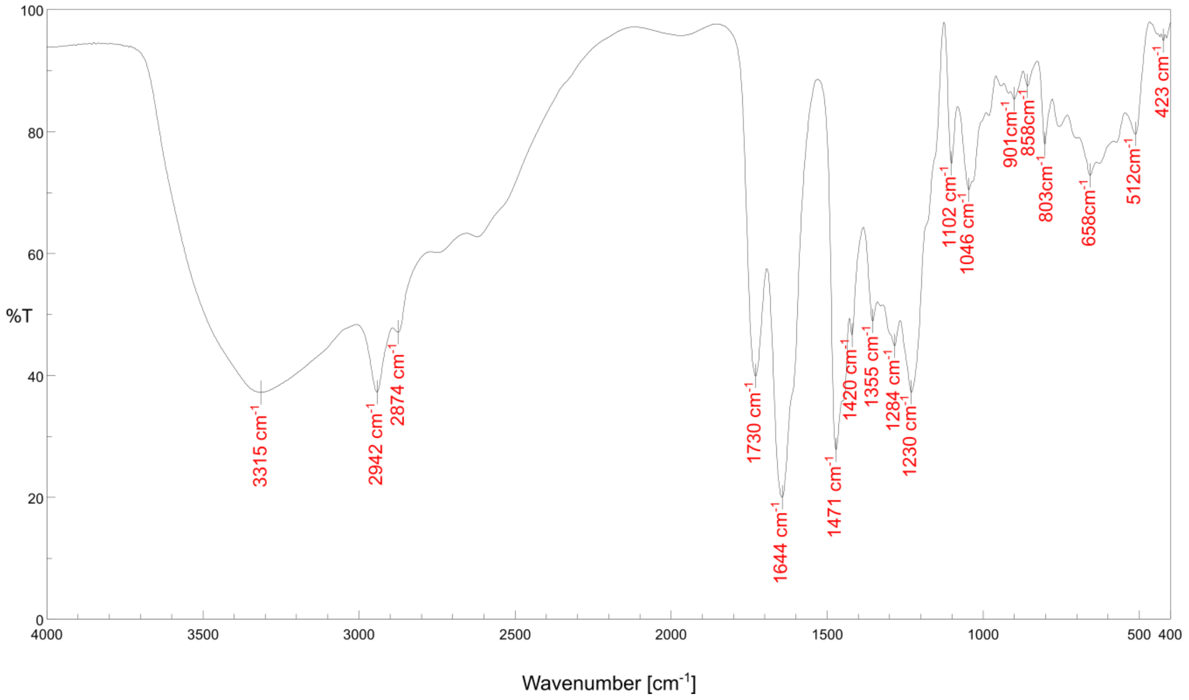

Figure S62. IR spectrum of L-**GluR** (KBr).

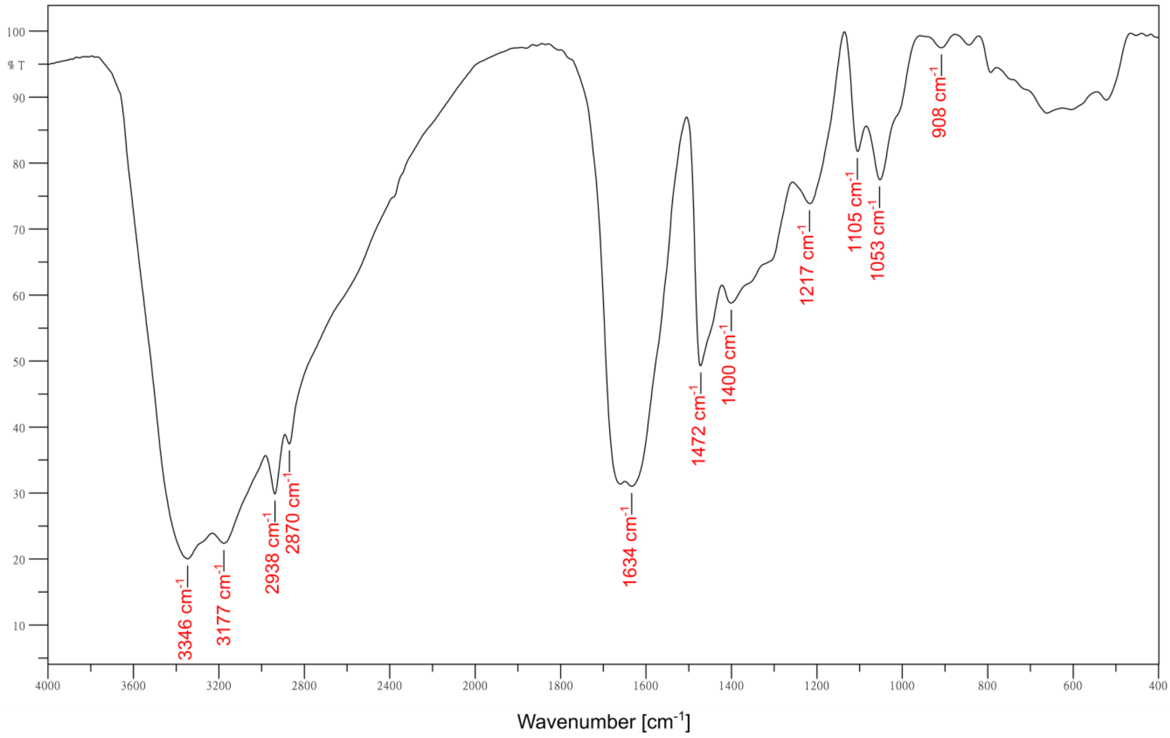

Figure S63. IR spectrum of L-**ArgR** (KBr).

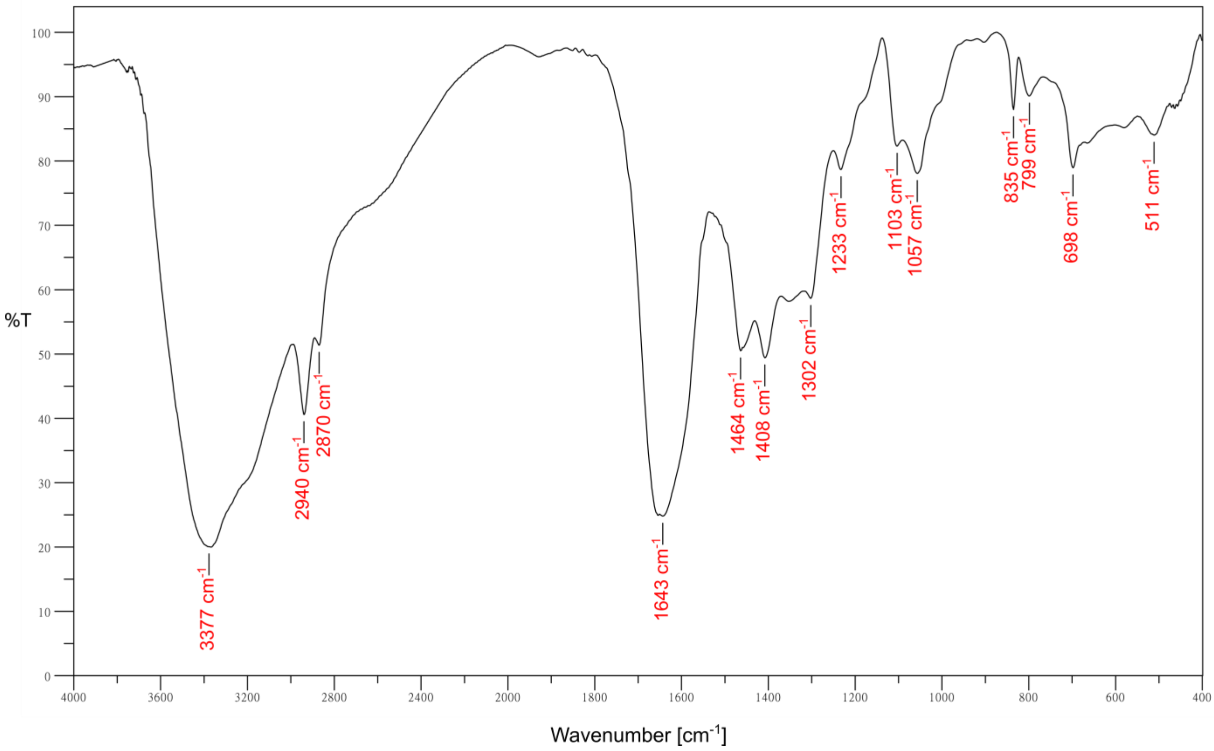

Figure S64. IR spectrum of D-**GluR**-L-**ArgR** (KBr).

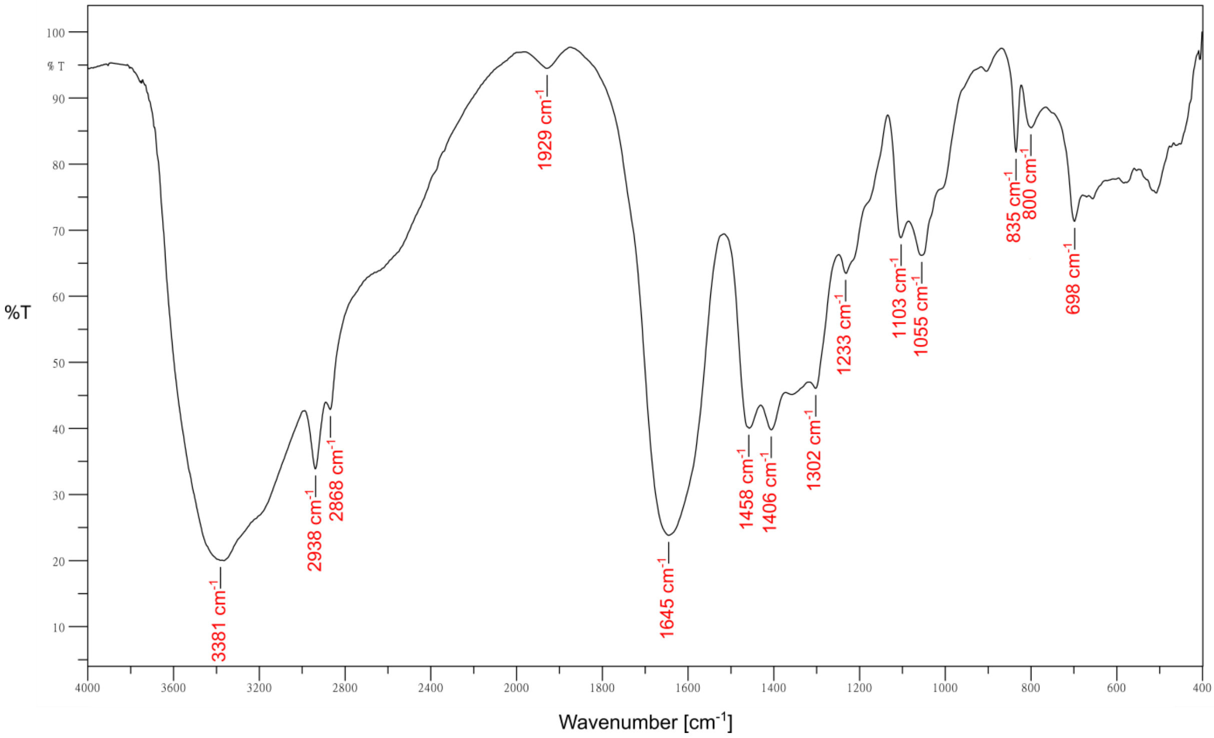

Figure S65. IR spectrum of L-**GluR**-L-**ArgR** (KBr).

5. MS spectra

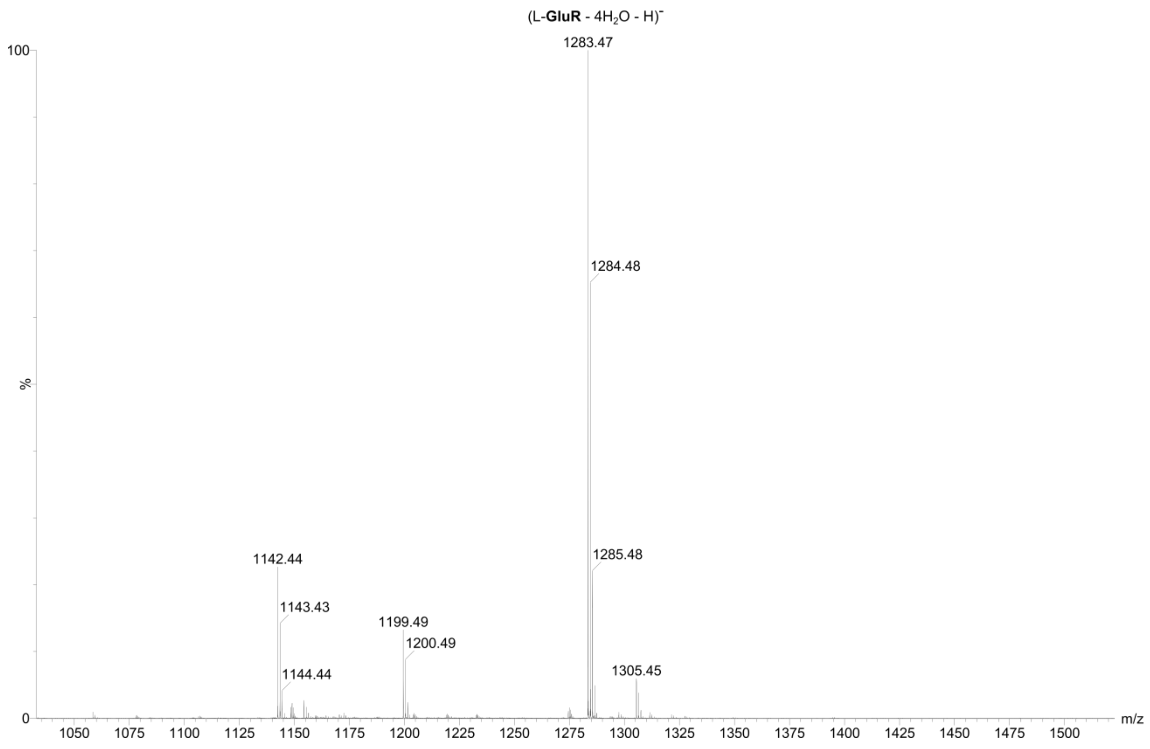

Figure S66 TOF-ESI of L-**GluR**.

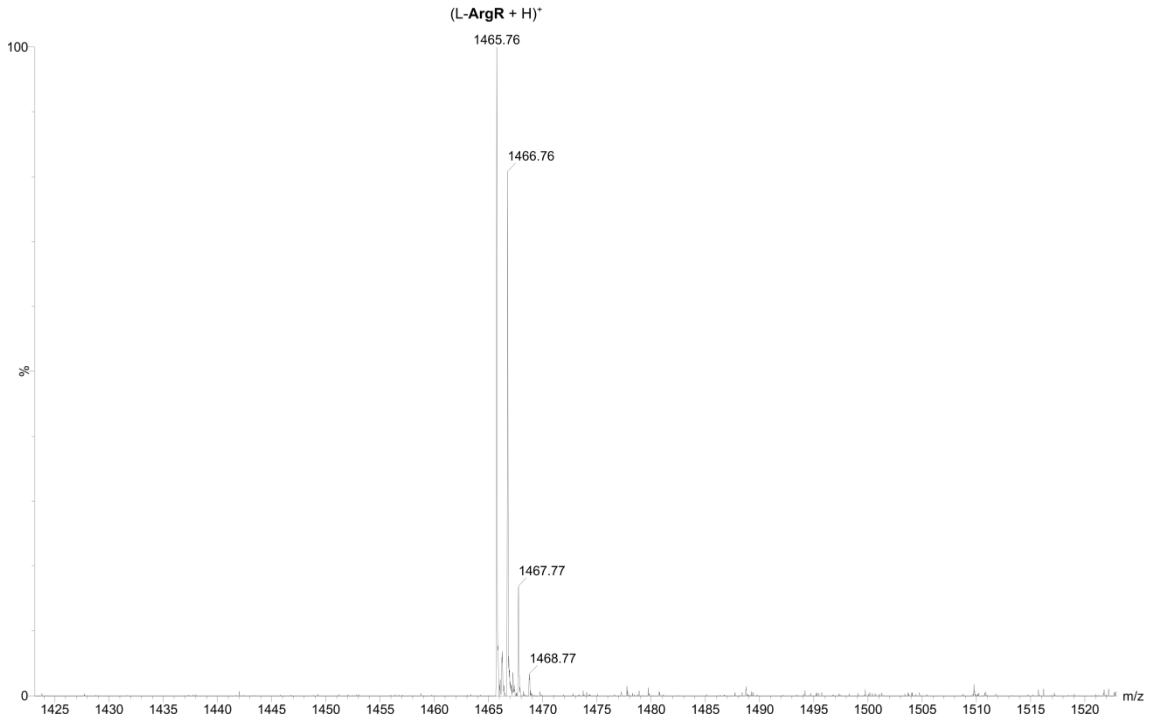

Figure S67. TOF-ESI of L-**ArgR**.

6. Calculations of the size of the capsule

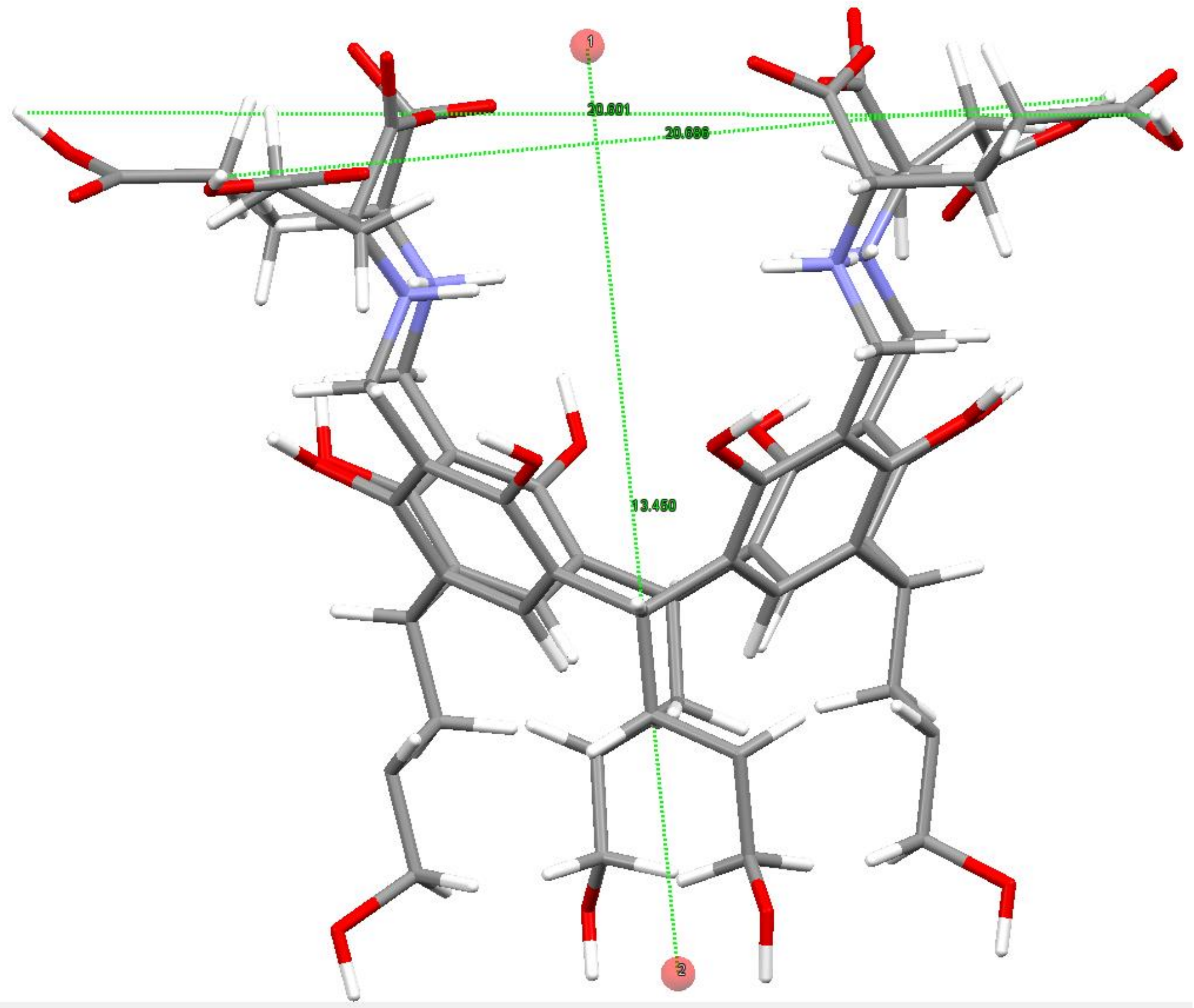

$$\bar{x}_r = \frac{20.601 + 20.686 + 13.450}{6} = 9.12\ \text{Å}$$

Figure S68. Approximation of the average size of L-**GluR**.

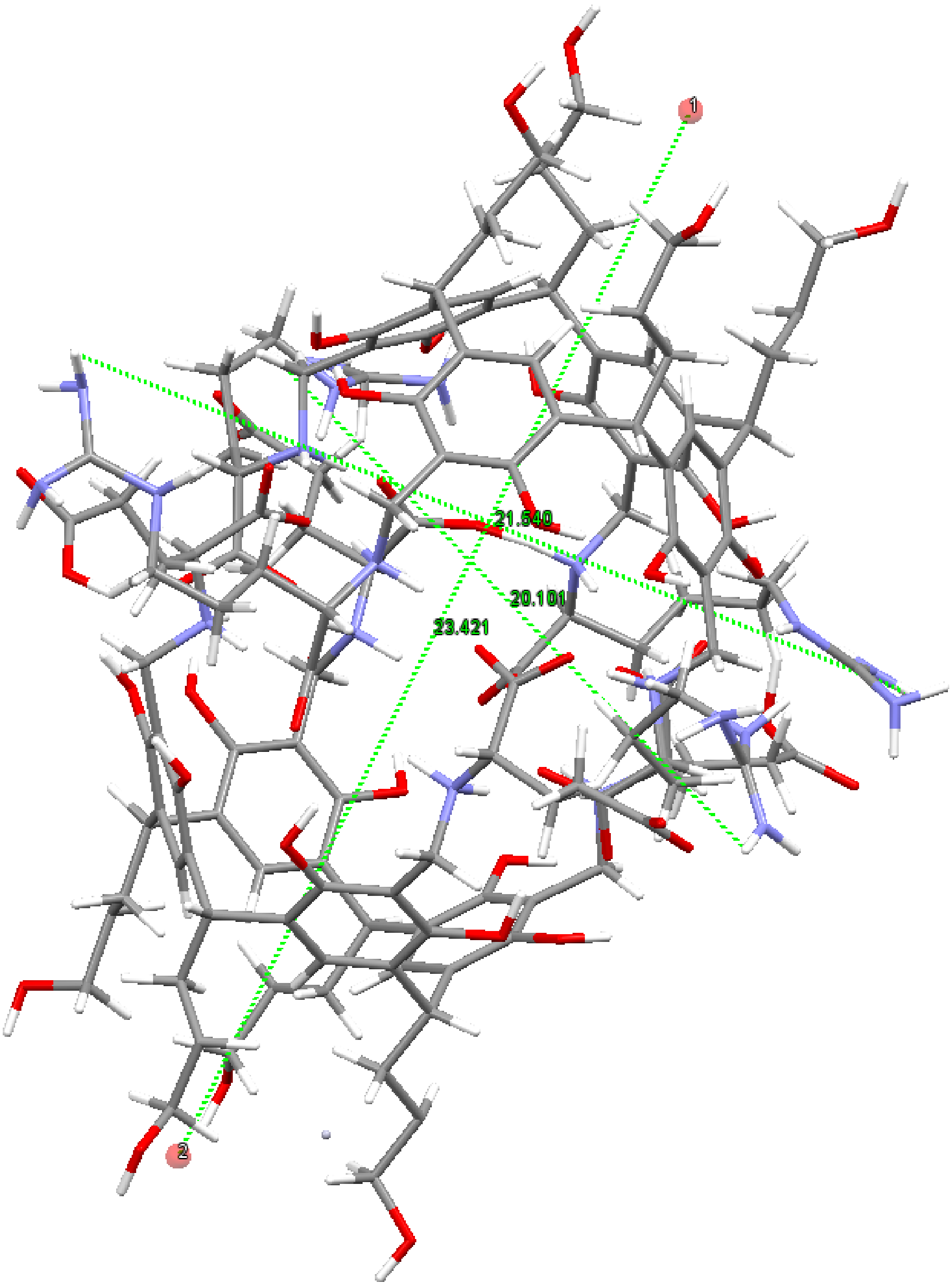

$$\bar{x}_r = \frac{23.421 + 20.101 + 21.540}{6} = 10.8 \text{ Å}$$

Figure S69. Approximation of the average size of L-**GluR**-L-**ArgR**.

---

[1] Gibb, B. C., Chapman, R. G., and Sherman, J. C. (1996). Synthesis of Hydroxyl-Footed Cavitands. *J. Org. Chem.*, 61, 1505-1509. doi.org/10.1021/jo951633c
